# Tracking regional brain growth up to age 13 in children born term and very preterm

**DOI:** 10.1038/s41467-020-14334-9

**Published:** 2020-02-04

**Authors:** Deanne K. Thompson, Lillian G. Matthews, Bonnie Alexander, Katherine J. Lee, Claire E. Kelly, Chris L. Adamson, Rod W. Hunt, Jeanie L. Y. Cheong, Megan Spencer-Smith, Jeffrey J. Neil, Marc L. Seal, Terrie E. Inder, Lex W. Doyle, Peter J. Anderson

**Affiliations:** 1 Victorian Infant Brain Study (VIBeS), Murdoch Children’s Research Institute, 50 Flemington Road, Parkville, VIC 3052 Australia; 2 Developmental Imaging, Murdoch Children’s Research Institute, 50 Flemington Road, Parkville, VIC 3052 Australia; 3 Department of Paediatrics, University of Melbourne, Grattan Street, Parkville, VIC 3010 Australia; 4 Florey Institute of Neuroscience and Mental Health, 30 Royal Parade, Parkville, VIC 3052 Australia; 5 Department of Pediatric Newborn Medicine, Brigham and Women’s Hospital, Harvard Medical School, 75 Francis St, Boston, MA 02115 USA; 6 Clinical Epidemiology and Biostatistics Unit, Murdoch Children’s Research Institute, 50 Flemington Road, Parkville, VIC 3052 Australia; 7 Neonatal Medicine, Royal Children’s Hospital, 50 Flemington Road, Parkville, VIC 3052 Australia; 8 Royal Women’s Hospital, 20 Flemington Road, Parkville, VIC 3052 Australia; 9 Department of Obstetrics and Gynaecology, University of Melbourne, Grattan Street, Parkville, VIC 3010 Australia; 10 Turner Institute for Brain and Mental Health, Monash University, Wellington Road, Clayton, VIC 3800 Australia; 11 Department of Pediatric Neurology, Washington University School of Medicine, 660 South Euclid Avenue, St. Louis, MO 63110 USA

**Keywords:** Developmental biology, Development of the nervous system, Paediatric research, Neurology

## Abstract

Serial regional brain growth from the newborn period to adolescence has not been described. Here, we measured regional brain growth in 216 very preterm (VP) and 45 full-term (FT) children. Brain MRI was performed at term-equivalent age, 7 and 13 years in 82 regions. Brain volumes increased between term-equivalent and 7 years, with faster growth in the FT than VP group. Perinatal brain abnormality was associated with less increase in brain volume between term-equivalent and 7 years in the VP group. Between 7 and 13 years, volumes were relatively stable, with some subcortical and cortical regions increasing while others reduced. Notably, VP infants continued to lag, with overall brain size generally less than that of FT peers at 13 years. Parieto–frontal growth, mainly between 7 and 13 years in FT children, was associated with higher intelligence at 13 years. This study improves understanding of typical and atypical regional brain growth.

## Abstract (web summary)

In this longitudinal study, the authors tracked the course of brain development from birth to adolescence (age 13 years) and examined the effects of very preterm birth. Very preterm children showed slower brain growth from age 0 (term equivalent) to age 7.

## Introduction

Brain development is most rapid and dynamic over the first 2 years of life^1^ and is known to occur in a posterior-to-anterior sequence. While it is well established that brain development continues into early adulthood^2^, and that individual brain regions and structures possess different maturational trajectories^3^, with possible sex differences^4^, until now there has been no longitudinal study tracking *in vivo* brain development from infancy to adolescence in the same individuals. Current knowledge regarding brain development is derived largely from post-mortem and cross-sectional magnetic resonance imaging (MRI) studies^5,6^, and previous longitudinal studies restricted to limited age windows^7,8^. This is partly because, until recently, there have been no available MRI analysis methods to accurately derive infant brain volumes in an age-appropriate way that match those typically obtained in older children and adults using the Desikan-Killiany cortical atlas and subcortical segmentation in FreeSurfer software^9^. With the release of the Melbourne Children’s Regional Infant Brain (M-CRIB) atlas^10^, we can now track typical and atypical growth of equivalent brain regions from infancy through to adolescence in finer anatomical detail than previously possible.

Infants born very preterm (VP; <32 weeks’ gestation) represent a unique population for studying brain development, as VP birth may disrupt the typical sequence of brain development^11^. MRI investigations at term-equivalent age have demonstrated (1) smaller brain volume in VP infants compared with full-term (FT) peers, (2) regional vulnerabilities to injury or disrupted development in VP infants^12,13^ and (3) evidence that preterm brain volume differences persist to early childhood^14,15^. While we would expect later maturing brain regions, including secondary and tertiary gyri that emerge later in foetal development or postnatally^3^, would be more likely developmentally disrupted by VP birth, regional brain maturational vulnerabilities have not been explicitly investigated from infancy to adolescence in the VP population.

Studying VP populations enables investigation into how and why brain developmental deficits arise. Some of the factors that have been shown to influence overall brain development include gestational age at birth^12^, sex^4^, brain injury, including white matter abnormality^16^, bronchopulmonary dysplasia^17^, prenatal growth (being born small for gestational age)^18^ and socio-economic status^19^. Although many of these factors have been previously associated with smaller brain volumes in VP populations^12,20^, their associations with regional brain development remain unclear. These factors may play a part in the differing vulnerabilities of particular brain regions to prematurity^12,13^.

Furthermore, the neurodevelopmental significance of early growth alterations in specific brain regions remains to be elucidated. Many studies have associated brain volumes with intelligence quotient (IQ) in both FT and preterm populations at various cross-sectional time-points^21,22^. Some studies have associated longitudinal growth of specific brain regions with IQ^14,15^. However, no study has yet investigated associations between IQ and longitudinal growth of multiple cortical and subcortical regions from infancy to adolescence in VP and FT children. While parieto–frontal regions have been implicated in IQ^23^, it is likely that the development of many cortical and subcortical brain regions contributes to intellectual functioning, given IQ is dependent on the integration of widespread brain networks^24^. Thus, a more comprehensive survey of the relationships between IQ and childhood regional brain growth is warranted.

Our longitudinal cohort consists of FT and VP infants with structural MRI at term-equivalent, 7 and 13 years of age, thereby providing a unique opportunity to study typical and atypical brain development, alongside associated risk factors and outcomes. The specific aims of this study were to: (1) describe the trajectory of regional brain growth in both FT and VP children from term-equivalent to 7, and from 7 to 13 years, and determine whether these trajectories differed between VP and FT groups and between the sexes, (2) determine whether higher social risk or perinatal factors, including brain injury, bronchopulmonary dysplasia, or small for gestational age, are associated with regional brain growth trajectories in VP children and (3) explore volumetric growth rate associations with IQ at 13 years, and whether these associations differed between VP and FT groups. We hypothesised that growth trajectories would be regionally specific, may demonstrate sex-specificity, and that VP brain growth trajectories would be delayed compared with the FT group, particularly in later maturing regions, such as the secondary and tertiary sulci and gyri. We expected adverse perinatal events and higher social risk to relate to poorer brain growth in a region-specific manner. Furthermore, we hypothesised that growth of widespread brain regions would be associated with higher IQ in both VP and FT groups, particularly within parieto–frontal regions.

We show that brain volumes increase between term-equivalent and 7 years, with faster regional brain development in the FT than the VP group. Perinatal brain abnormality influences growth over the first 7 years of life in VP children. Between 7 and 13 years, brain volumes are relatively stable, where some subcortical and cortical regions increase while others reduce. We also demonstrate associations between greater growth of the parieto–frontal network during middle childhood and higher intelligence at 13 years in FT children.

## Results

### Participant characteristics

In all, 193 VP (born <30 weeks’ gestation or very low birthweight, <1250 g) and 34 FT (born ≥37 and ≤41 weeks’ gestation) infants had usable volumetric data at term, 152 VP and 34 FT children had usable data at 7 years, and 140 VP and 26 FT children had usable data at 13 years. In all, 216 VP and 45 FT children had usable data at any time-point, and all these children were included in the analyses. The characteristics of the participants are summarised in Table 1. Raw mean volumes for each brain region by group are presented in Supplementary Table 1 for the term-equivalent (a), 7-year (b) and 13-year (c) time-points.

**Table 1:** Characteristics of the very preterm and full-term participants who contributed any volumetric data at any of the time-points.

	Very preterm, *n* = 216	Full-term, *n* = 45
Gestational age at birth in weeks, mean (SD), min to max	27.5 (1.9), 22–32	39.0 (1.2), 37–41
Birthweight in grams, mean (SD), min to max	959 (223), 414–1425	3307 (485), 2390–4290
Birthweight SD score^a^, mean (SD)	−0.6 (0.9)	0.1 (0.9)
Small for gestational age^a^, *n* (%)	19 (9)	1 (2)
Multiple birth, *n* (%)	91 (42)	2 (4)
Male sex, *n* (%)	112 (52)	24 (53)
Administered postnatal corticosteroids, *n* (%)	21 (10)^b^	0 (0)
Bronchopulmonary dysplasia^c^, *n* (%)	73 (34)	0 (0)
Infection^d^, *n* (%)	79 (37)	0 (0)
Moderate/Severe brain abnormality^e^, *n* (%)	58 (27)^f^	0 (0)
Cystic periventricular leucomalacia^g^, *n* (%)	9 (4)	0 (0)
Intraventricular haemorrhage grade 3 or 4^g^, *n* (%)	8 (4)	0 (0)^h^
Higher social risk at 2-year assessment, *n* (%)	115 (61)^i^	7 (17)^j^
Postmenstrual age at neonatal MRI in weeks, mean (SD), min to max	40.6 (1.0), 38–43^k^	41.1 (0.9), 39–43^l^
Age at 0-year MRI in years, mean (SD), min to max	0.01 (0.03), −0.18–0.05^k^	0.02 (0.02), −0.02–0.05^l^
Weight at neonatal MRI in grams, mean (SD), min to max	3024 (511), 1945–4678^k^	3470 (472), 2530–4500^m^
Age at 7-year MRI in years, mean (SD), min to max	7.5 (0.3), 6.6–8.1^n^	7.6 (0.2), 7.2–8.0^l^
Weight at 7-year MRI in kg, mean (SD), min to max	25 (5), 17–55^o^	27 (5), 20–43^l^
Age at 13-year MRI in years, mean (SD), min to max	13.3 (0.4), 11.8–14.9^p^	13.4 (0.6), 12.4–15.2^q^
Weight at 13-year MRI in kg, mean (SD), min to max	50 (12), 30–89^r^	52 (14), 14–82^s^
IQ at 13 years, mean (SD), min to max	100 (18), 49–138^t^	112 (13), 72–136^l^
Higher social risk at 13-year assessment, *n* (%)	98 (58)^u^	5 (15)^l^

All ages for the very preterm group have been corrected for prematurity.

*SD* standard deviation.

^a^Birthweight SD score was calculated relative to the British Growth Reference dataset. Small for gestational age was defined as birthweight more than two SDs below the mean. ^b^*n* = 215. ^c^Bronchopulmonary dysplasia was defined as requirement for supplemental oxygen at 36 weeks. ^d^Infection was defined as sepsis and/or proven necrotising enterocolitis. ^e^Scored on term-equivalent age MRI using the Kidokoro et al.^25^ qualitative reporting system. ^f^*n* = 214. ^g^Cystic periventricular leucomalacia and intraventricular haemorrhage were recorded from cranial ultrasound; intraventricular haemorrhage was graded according to Papile et al.^64^. ^h^*n* = 44. ^i^*n* = 188. ^j^*n* = 41. ^k^*n* = 193. ^l^*n* = 34. ^m^*n* = 33. ^n^*n* = 152. ^o^*n* = 150. ^p^*n* = 140. ^q^*n* = 26. ^r^*n* = 137. ^s^*n* = 25. ^t^*n* = 175. ^u^*n* = 168.

### Regional brain growth trajectories from term-equivalent to 13 years

Across both birth groups and sexes, all brain regions, except lateral ventricles, increased significantly in volume between term-equivalent and 7 years (Supplementary Fig. 1, Supplementary Tables 2a and 3a). After adjusting for total brain tissue volume (TBV), statistical significance weakened for some regions, but in general cortical and cerebellar volumes still increased, however, total white matter and thalami volumes decreased over this period (Supplementary Fig. 2, Supplementary Tables 2a and 3a). Between 7 and 13 years, regional volumes generally remained stable, although there was evidence that intracranial volume (ICV), cerebrospinal fluid (CSF), brainstem, left and right amygdalae and thalami, left pallidum and the right pericalcarine region in the occipital lobe continued to increase in volume (Supplementary Fig. 1, Supplementary Tables 2b and 3b). In contrast, some cortical volumes decreased from 7 to 13 years, including subregions in the frontal (bilateral frontal pole), lateral temporal (right banks of superior temporal sulcus and left transverse temporal regions) and parietal (left inferior and superior parietal) cortices (Supplementary Fig. 1, Supplementary Tables 2b and 3b). After adjusting for TBV, the brainstem, left and right amygdalae and thalami, left pallidum and right pericalcarine still increased significantly between 7 and 13 years, while bilateral frontal poles, left inferior and superior parietal, left transverse temporal and right banks of superior temporal sulcus reduced in volume (Supplementary Fig. 2, Supplementary Tables 2b and 3b).

### Growth trajectories in very preterm and full-term children

**Fig. 1:**
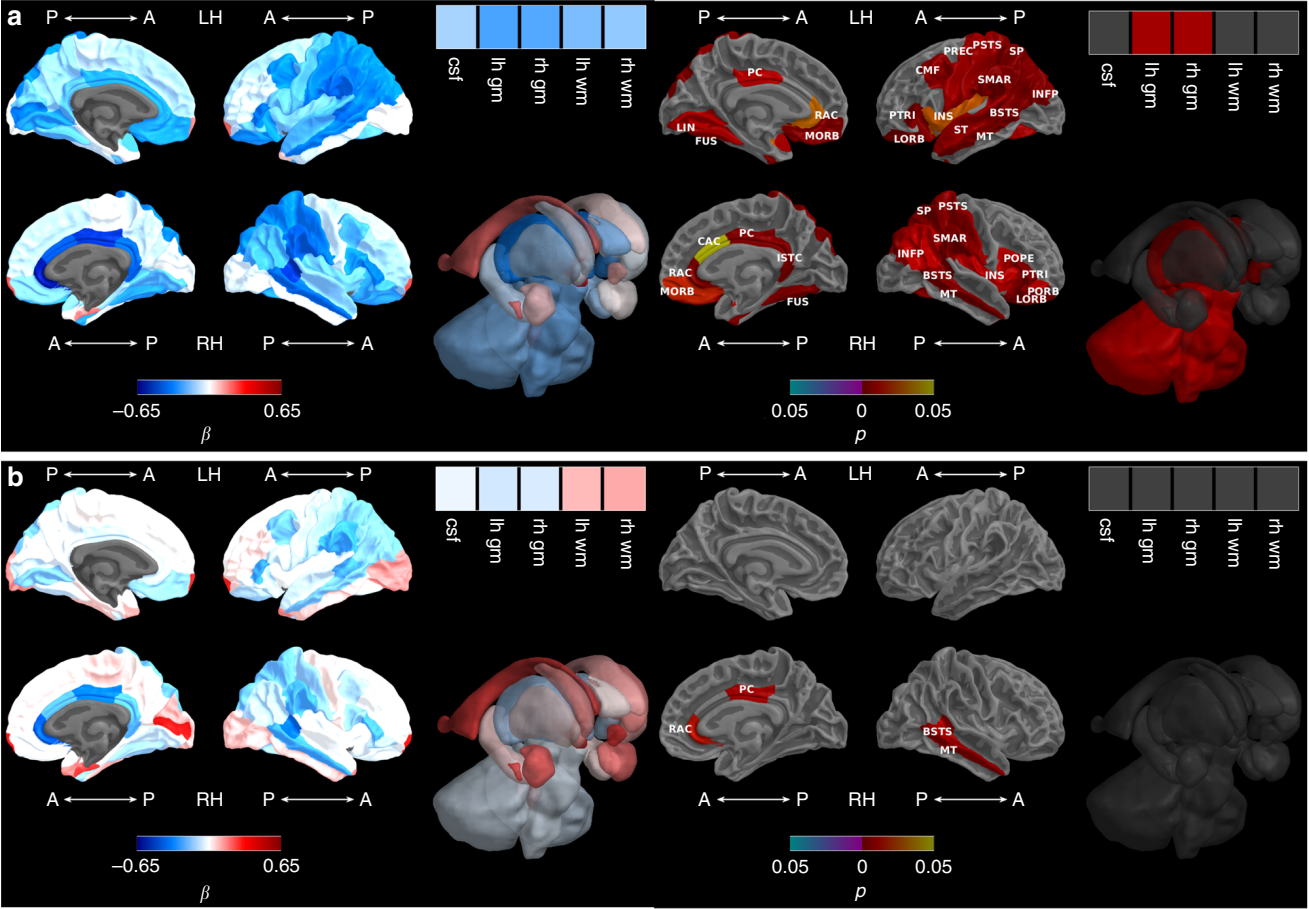
Very preterm versus full-term brain growth from term-equivalent to 7 years. **a** Before and **b** after adjusting for total brain tissue volume. Note: Group-by-time regression coefficients from linear mixed effects models (β) in blue refer to negative associations (slower growth in the very preterm compared with the full-term group) and in red refer to positive associations (faster growth in the very preterm compared with the full-term group), with darker coloured regression coefficients indicating stronger associations; Interaction *p* values are false discovery rate corrected and darker coloured *p* values indicate stronger significance; Boxes in the top right corner of the figures represent global brain volumes. A = anterior, P = posterior, LH = left hemisphere, RH = right hemisphere, csf = cerebrospinal fluid, gm = grey matter, wm = white matter, BSTS = banks of superior temporal sulcus, CAC = caudal anterior cingulate, CMF = caudal middle frontal, FUS = fusiform, INFP = inferior parietal, INS = insula, ISTC = isthmus cingulate, LORB = lateral orbitofrontal, LIN = lingual, MORB = medial orbitofrontal, MT = middle temporal, POPE = pars opercularis, PORB = pars orbitalis, PTRI = pars triangularis, PSTS = postcentral, PC = posterior cingulate, PREC = precentral, RAC = rostral anterior cingulate, SP = superior parietal, ST = superior temporal, SMAR = supramarginal.

Between term-equivalent and 7 years, there was faster growth in FT compared with VP children in ICV, TBV and bilateral whole cortex, basal ganglia and thalamic regions (bilateral pallida and thalami), brainstem, bilateral cerebella and insulae, and some frontal (bilateral pars triangularis, lateral orbitofrontal, medial orbitofrontal, right pars orbitalis and pars opercularis, left precentral and caudal middle frontal), limbic (bilateral rostral anterior cingulate and posterior cingulate, right isthmus cingulate and caudal anterior cingulate), medial temporal (bilateral fusiform), lateral temporal (bilateral middle temporal, banks of superior temporal sulcus, left superior temporal), parietal (bilateral inferior parietal, postcentral, superior parietal, and supramarginal) and occipital (left lingual) cortical regions (Fig. 1a, Supplementary Fig. 1, Supplementary Table 2a). After adjusting for TBV, there remained evidence of less overall growth between term-equivalent and 7 years in the VP compared with the FT group within four regions, located in the limbic structures (right rostral anterior cingulate and posterior cingulate), and lateral temporal lobe (right middle temporal and right banks of superior temporal sulcus) (Fig. 1b, Supplementary Fig. 2, Supplementary Table 2a). Growth from 7 to 13 years did not statistically differ between groups for any of the regions before (Supplementary Fig. 1, Supplementary Table 2b) or after (Supplementary Fig. 2, Supplementary Table 2b) adjusting for TBV. However, it is of note that on average, TBV still appeared lower in VP than FT children at 13 years based on paired *t*-tests [mean (standard deviation (SD)): VP 1213.49 (118.91); FT 1310.72 (111.63); mean difference 97.23 ml, *p* < 0.001); Supplementary Table 1 and Supplementary Fig. 1].

### Growth trajectories in male and female children

There was greater growth from term-equivalent to 7 years in males than females in the left and right cerebral white matter (Supplementary Fig. 1, Supplementary Table 3a), but this did not remain after adjusting for TBV (Supplementary Fig. 2, Supplementary Table 3a). Volume growth from 7 to 13 years did not statistically differ by sex for any of the regions, either before (Supplementary Fig. 1, Supplementary Table 3b) or after adjusting for TBV (Supplementary Fig. 2, Supplementary Table 3b).

### Group-time-sex interaction

Based on the linear mixed effects models, group differences (VP or FT) in growth trajectories did not vary by sex for any regions, before or after adjusting for TBV, either between term-equivalent and 7 years (interaction *p* > 0.5) or 7 and 13 years (interaction *p* > 0.9).

### Perinatal and social factors

**Fig. 2:**
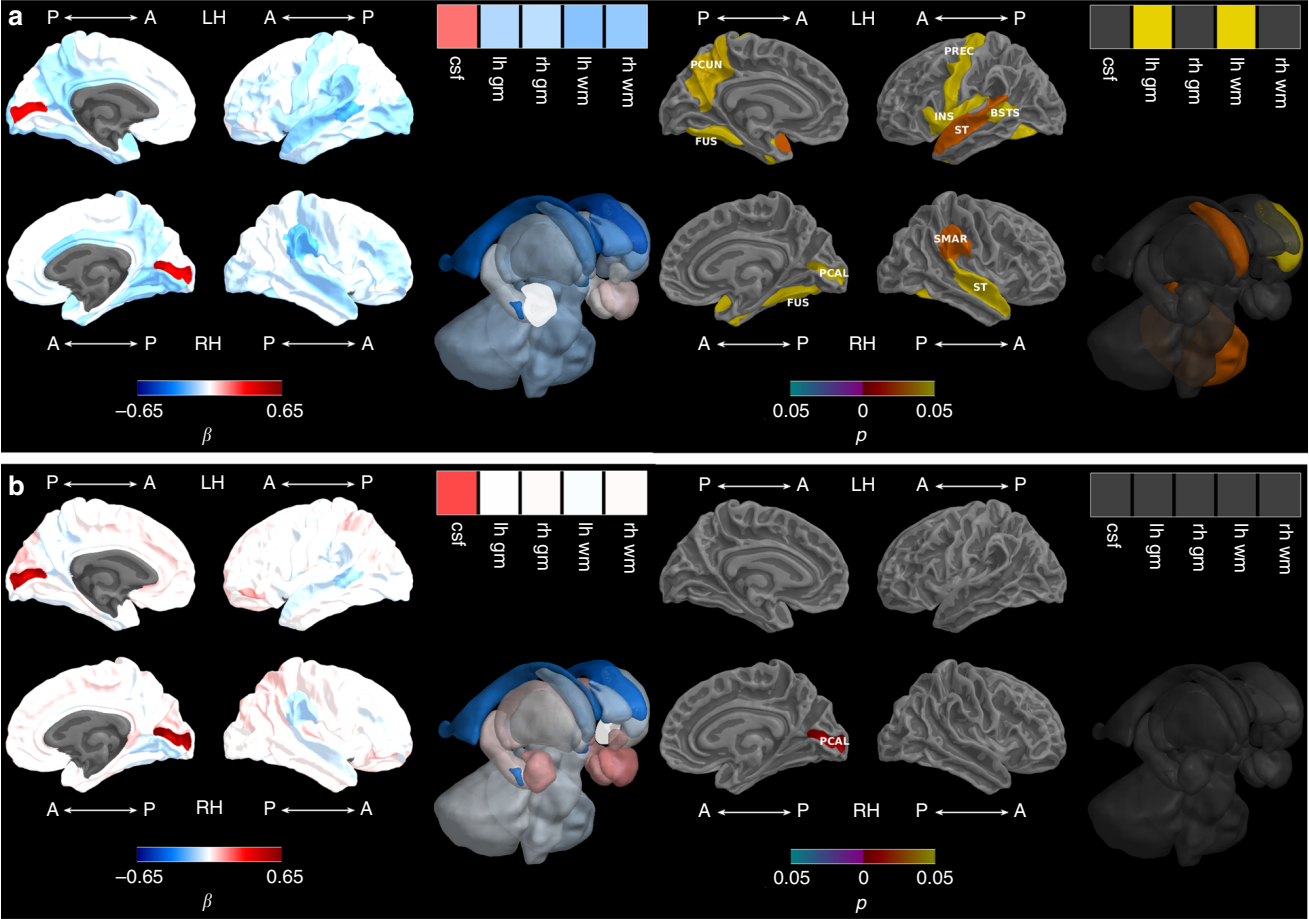
Brain abnormality and brain growth from term-equivalent to 7 years. **a** Before and **b** after adjusting for total brain volume. Note: Brain abnormality-by-time regression coefficients from linear mixed effects models (β) in blue refer to negative associations (slower growth in children with brain abnormality compared with children with no or mild brain abnormality) and in red refer to positive associations (faster growth in children with brain abnormality compared with children with no or mild brain abnormality), with darker coloured regression coefficients indicating stronger associations; Interaction *p* values are false discovery rate corrected and darker coloured *p* values indicate stronger significance; Boxes in the top right corner of the figures represent global brain volumes. A = anterior, P = posterior, LH = left hemisphere, RH = right hemisphere, csf = cerebrospinal fluid, gm = grey matter, wm = white matter, BSTS = banks of superior temporal sulcus, FUS = fusiform, INS = insula, PCAL = pericalcarine, PCUN = precuneus, PREC = precentral, ST = superior temporal, SMAR = supramarginal.

Before adjusting for TBV, there was poorer growth from term-equivalent to 7 years in VP children who had moderate-to-severe perinatal brain abnormality compared with VP children who had no or mild brain abnormality using the Kidokoro scoring system^25^, namely for TBV, left white matter and left cortex volume, left cerebellar hemisphere and insula, left and right caudate, and cortical regions within the lateral temporal lobe (bilateral superior temporal and left banks of superior temporal sulcus), medial temporal lobe (bilateral fusiform), frontal lobe (left precentral), parietal lobe (right supramarginal and left precuneus), and faster growth in the right pericalcarine region of the occipital lobe (Fig. 2a, Supplementary Table 4a). There remained faster growth between term-equivalent and 7 years for the right pericalcarine after adjusting for TBV (Fig. 2b, Supplementary Table 4a). Moderate-to-severe brain abnormality was not associated with growth between 7 and 13 years, either before or after adjusting for TBV (Supplementary Table 4b).

**Fig. 3:**
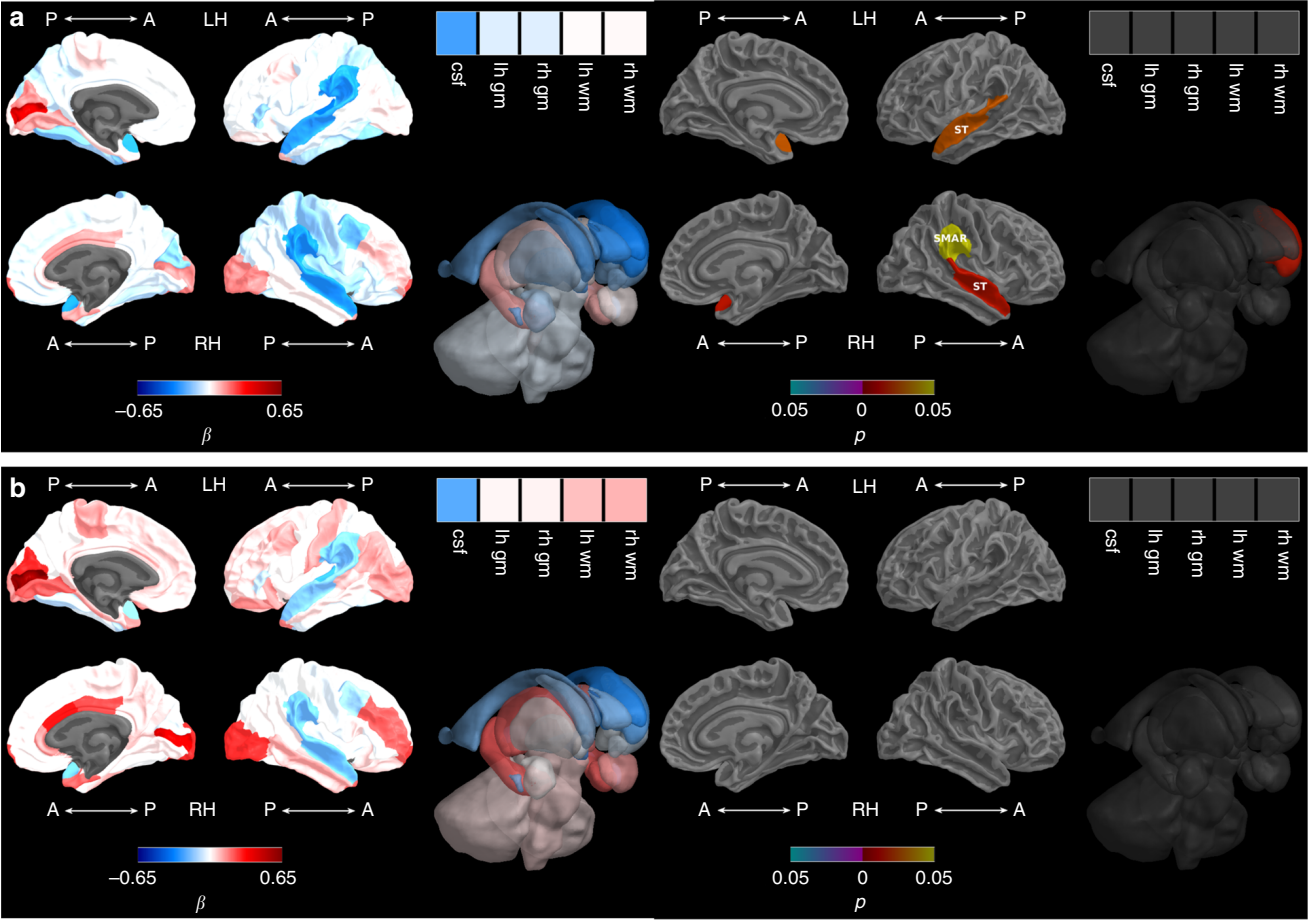
Growth restriction and brain growth from term-equivalent to 7 years. **a** Before and **b** after adjusting for total brain volume. Note: Small for gestational age-by-time regression coefficients from linear mixed effects models (β) in blue refer to negative associations (slower growth in children born small compared with appropriate for gestational age) and in red refer to positive associations (faster growth in children born small compared with appropriate for gestational age), with darker coloured regression coefficients indicating stronger associations; Interaction *p* values are false discovery rate corrected and darker coloured *p* values indicate stronger significance; Boxes in the top right corner of the figures represent global brain volumes. A = anterior, P = posterior, LH = left hemisphere, RH = right hemisphere, csf = cerebrospinal fluid, gm = grey matter, wm = white matter, ST = superior temporal, SMAR = supramarginal.

VP children born small for gestational age had poorer growth of the bilateral superior temporal, right supramarginal (parietal lobe) and left caudate regions between term-equivalent and 7 years (Fig. 3a, Supplementary Table 5a), but this was not statistically significant after adjusting for TBV (Fig. 3b, Supplementary Table 5a). Being born small for gestational age was not associated with volumetric growth from 7 to 13 years in any region before adjusting for TBV, but after adjusting for TBV, poorer left insula growth was associated with being born small for gestational age (Supplementary Table 5b).

Bronchopulmonary dysplasia was not associated with volumetric growth between term-equivalent and 7 years before adjusting for TBV, but was associated with poorer growth in the right superior temporal region after adjusting for TBV (Supplementary Table 6a). Bronchopulmonary dysplasia was not associated with volumetric growth between 7 and 13 years in any region, before or after adjusting for TBV (Supplementary Table 6b).

Higher social risk at 2 years was not associated with volumetric growth from term-equivalent to 7 years before adjusting for TBV, but was associated with poorer growth in the cortex as a whole after adjusting for TBV (Supplementary Table 7a). Higher social risk at 2 years was not associated with volumetric growth between 7 and 13 years in any region, before or after adjusting for TBV (Supplementary Table 7b).

### Association between volumetric growth and intelligence

**Fig. 4:**
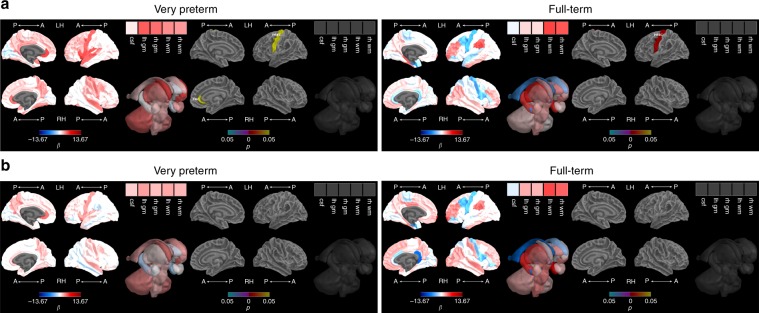
Intelligence and brain growth from term-equivalent to 7 years. **a** Adjusting for sex and age at IQ assessment and **b** additionally adjusting for total brain volume growth and social risk. Note: IQ-by-time regression coefficients from linear regression models (β) in blue refer to negative associations (slower growth associated with higher IQ scores) and in red refer to positive associations (faster growth associated with higher IQ scores), with darker coloured regression coefficients indicating stronger associations; *p* values are false discovery rate corrected and darker coloured *p* values indicate stronger significance; Boxes in the top right corner of the figures represent global brain volumes. A = anterior, P = posterior, LH = left hemisphere, RH = right hemisphere, csf = cerebrospinal fluid, gm = grey matter, wm = white matter, PREC = precentral, RAC = rostral anterior cingulate.

For most brain regions, there were no significant group interactions for associations between term-equivalent to 7-year volumetric change and IQ. Birth groups were combined in the absence of a group interaction, with no significant associations between term-equivalent to 7-year volumetric change and IQ, either before or after adjusting for TBV change and social risk (Supplementary Table 8a). Between term-equivalent and 7 years, volumetric growth associations with IQ differed by birth group for some brain regions (interaction *p* < 0.05). Faster volumetric growth of the left precentral area of the frontal lobe in the VP group, and slower volumetric growth of this region in the FT group, was associated with higher IQ at 13 years (Fig. 4a, Supplementary Table 8a). Furthermore, faster right rostral anterior cingulate (limbic) growth between term-equivalent and 7 years was associated with higher IQ for the VP group only (Supplementary Table 8a). Following adjustment for TBV growth and social risk at 13 years, there were no longer group interactions, and associations between volumetric growth and IQ were non-significant for the combined VP and FT group (Fig. 4b, Supplementary Table 8a).

**Fig. 5:**
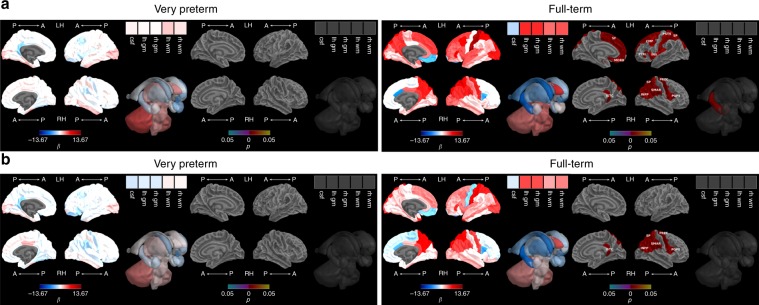
Intelligence and brain growth from 7 to 13 years. **a** Adjusting for sex and age at IQ assessment and **b** additionally adjusting for total brain volume growth and social risk. Note: IQ-by-time regression coefficients from linear regression models (β) in blue refer to negative associations (slower growth associated with higher IQ scores) and in red refer to positive associations (faster growth associated with higher IQ scores), with darker coloured regression coefficients indicating stronger significance; *p* values are false discovery rate corrected and darker coloured *p* values indicate stronger associations; Boxes in the top right corner of the figures represent global brain volumes. A = anterior, P = posterior, LH = left hemisphere, RH = right hemisphere, csf = cerebrospinal fluid, gm = grey matter, wm = white matter, CMF = caudal middle frontal, INFP = inferior parietal, INS = insula, ISTC = isthmus cingulate, MORB = medial orbitofrontal, POPE = pars opercularis, PTRI = pars triangularis, PSTS = postcentral, PREC = precentral, SF = superior frontal, SP = superior parietal, SMAR = supramarginal.

Between 7 and 13 years, volumetric growth associations with IQ differed by birth group in some brain regions (interaction *p* < 0.05). Growth in the FT group was associated with higher IQ for the left insula, regions of the parietal lobe (bilateral superior parietal, left postcentral, right supramarginal and inferior parietal), frontal lobe (right precentral, left superior frontal, caudal middle frontal and pars triangularis), and limbic system (right isthmus cingulate) (Fig. 5a, Supplementary Table 8b). Volumetric decline between 7 and 13 years in the FT group in the right hippocampus (temporal lobe), right pars opercularis and left medial orbitofrontal region (both in the frontal lobe) was associated with higher IQ (Fig. 5a, Supplementary Table 8b). These relationships were not present in the VP group (Fig. 5a, Supplementary Table 8b). Many of the positive associations in the FT group remained after adjusting for TBV growth and social risk, including regions of the parietal lobe (right superior parietal, supramarginal and inferior parietal), frontal lobe (right precentral), and limbic system (right isthmus cingulate). The negative association for the right pars opercularis (frontal) region also remained (Fig. 5b, Supplementary Table 8b). For the remaining brain regions where birth groups were combined in the absence of a group interaction, there were no significant associations between 7 to 13-year volumetric change and IQ, either before or after adjusting for TBV change and social risk (Supplementary Table 8b).

## Discussion

This study presents volumetric trajectories for 68 cortical and 14 subcortical brain regions from infancy to adolescence in a unique serially studied cohort of VP and FT children. There was a general increase in brain volume between term-equivalent and 7 years for all children. Furthermore, there was faster growth in FT compared with VP children over this period, with specific limbic and temporal region involvement independent of TBV. In contrast, regional volumes generally remained stable between 7 and 13 years across all children, although amygdalae, thalami, brainstem, left pallidum and an occipital cortical region (right pericalcarine) continued to grow, while some cortical regions decreased in volume. Interestingly, brain growth between 7 and 13 years did not differ between VP and FT groups, although VP infants continued to lag, with overall brain size generally still less than that of their FT peers at 13 years. White matter grew more rapidly in males than females between term-equivalent and 7 years, but there were no sex differences once accounting for TBV. The main factor associated with poorer brain growth in VP children from term-equivalent to 7 years was perinatal brain abnormality, but interestingly greater growth of the right pericalcarine occipital region between term-equivalent and 7 years was associated with brain abnormality, and this relationship was the only one independent of TBV. Being small for gestational age at birth was associated with slower growth from term-equivalent to 7 years in the superior temporal and supramarginal cortices and caudate nuclei, however these associations were not independent of TBV. The strongest associations with IQ at 13 years were for growth in the parieto–frontal network between 7 and 13 years in FT children.

In the current study, we observed increases in brain volumes from term-equivalent age to 7 years across all children, consistent with reports of rapid brain development during the first years of life^1^. Indeed, this period is characterised by rapid axonal development, myelination, and enhanced neural connectivity, resulting in total cerebral volume increasing from one-third of its adult volume at birth to ~90% by school age^1,26^. Furthermore, the observed maturation timeline appears to be consistent with that in post-mortem studies reporting dramatic neuronal development from 29 weeks’ gestation through to 6–9 years^5,27^.

Our results extend these investigations to adolescence, where brain trajectory differences became more region-specific, as hypothesised. We demonstrate that most brain growth has slowed, ceased or regressed by 13 years, apart from subcortical grey matter structures and the pericalcarine region of the occipital lobe. Our volumetric decline in several cortical regions between 7 and 13 years is consistent with previous studies demonstrating regional grey matter reductions around adolescence, particularly within frontal, temporal and parietal regions^28^. Various biological mechanisms associated with typical maturational processes are proposed to underlie the lack of brain growth over this period, including elimination of synapses, slowed cell growth and reduced dendritic arbor size^5^.

The current study demonstrates reduced brain growth in VP compared with FT children from term-equivalent age to 7 years, although it is possible that growth is disproportionate in VP infants even prior to term-equivalent age. Indeed, the neonatal intensive care environment may exert stressors that expose the VP brain to maturational vulnerability when compared with the *in utero* environment of FT infants^29^. Importantly, the current findings are consistent with earlier reports in this cohort^14,15^, extending this work by demonstrating detailed regional maturational vulnerabilities. Further, our findings of significantly reduced early growth in VP compared with FT children within some limbic (cingulate) and lateral temporal cortical regions even after adjusting for TBV are in line with our hypothesis, and indicate that growth of these later maturing cortical regions may be particularly vulnerable to prematurity, over and above whole brain effects. These regions develop during the VP period, where the middle and superior temporal sulci and gyri emerge between 32–35 weeks’ gestation, and the cingulate emerges between 36–39 weeks^3^. Indeed, cingulate and temporal cortical regions have previously been shown to be thinner in 7–10-year-olds born VP compared with FT children^30^. However, caution is recommended when interpreting these findings, as TBV adjustment may not fully account for total brain size since regions scale differently during development^31^.

Importantly the current study demonstrated similar growth trajectories between VP and FT children from 7 to 13 years, although VP children continued to lag with brain volumes generally smaller in VP compared with FT children at these time-points. These findings suggest a lack of ‘catch-up’ growth in VP infants, potentially reflective of an altered brain developmental course following early maturational aberrancies. In line with these findings, a longitudinal study from 8 to 9 years found that very low birth weight preterm children did not differ from FT children in terms of cortical thickness and surface area trajectories^32^. Another study extending this developmental time frame through 15–20 years also showed no group by time interaction in cortical thickness and surface area trajectories, suggesting that preterm and FT groups do not diverge during adolescence^33^. Indeed, it has been suggested that preterm brain growth beyond the early postnatal years may not compensate for poorer earlier growth^34^. In line with this, recent work has demonstrated persisting brain volume reductions during adulthood in VP infants, particularly global grey matter volume^35^. In contrast to our findings, a longitudinal study from 8 to 12 years found that cerebral grey matter reduced less rapidly, while white matter increased less rapidly in VP compared with control children^36^. Some earlier studies indicate further conflicting evidence of potential brain developmental ‘catch-up’, which may reflect methodological differences between studies, or a different era of VP infants in terms of medical treatment and outcomes^37,38^.

There was a lack of evidence for sex differences in regional brain growth in the current study. Apparent faster growth in the cerebral white matter from term-equivalent to 7 years in males compared with females was no longer evident upon TBV adjustment, suggesting that these differences may be explained by growth in TBV; known to be larger in males^4,39^. These findings provide further insight into a largely unanswered question; how sexual dimorphism contributes to the complexity of human brain maturational trajectories^40^.

The presence of perinatal moderate-to-severe brain abnormality on MRI was associated with slower TBV growth from term-equivalent to 7 years in VP children compared with those who had no or mild brain abnormality, suggesting that the adverse effects of neonatal brain injury commonly reported in preterm infants persist into childhood^12,41,42^. It is likely that brain abnormality contributed to poorer and consequently slower brain growth in VP than FT children between term-equivalent and 7 years. While the underlying mechanisms for these disturbances are yet to be fully elucidated, hypoxic–ischaemic effects are likely implicated^43^ alongside inflammatory processes involving necrosis, apoptosis, astrogliosis, microgliosis and preoligodendrocyte injury. Reduced growth for particular regions from term-equivalent to 7 years in children with brain abnormality was not independent of TBV; contrary to our hypothesis that findings would be region-specific. Importantly, primary white matter injury has been proposed to result in secondary disturbances in grey matter structures^16^, potentially explaining why we found brain abnormality to have a widespread influence on TBV. Of note, we did not find relationships between perinatal brain injury and growth from 7 to 13 years, suggesting that the effects of early brain abnormality may be restricted to the neonatal and early childhood periods.

Interestingly, volumetric *growth* in the pericalcarine region from term-equivalent to 7 years was associated with brain abnormality in VP children, even after adjusting for TBV. Considering this is a traditionally late-developing occipital region, as further suggested by our finding of its persisting growth between 7 and 13 years, it may be that early growth of this brain region reflects a developmental aberration, triggered by brain abnormality. In keeping with this finding, the pericalcarine region has been reported to be larger in those born earlier both at term^13^, and in adulthood^36^. This may be attributed to accelerated brain maturation due to increased sensory input with greater time *ex utero*. Indeed, the bright lighting common in open intensive care units^44^ would be particularly relevant in the case of the pericalcarine region, known to be involved in vision.

In the current study, VP children born small for gestational age had poorer growth of the bilateral superior temporal, right supramarginal and left caudate regions from term-equivalent to 7 years compared with appropriate for gestational age peers, although the evidence for this did not remain following TBV adjustment and was not present from 7 to 13 years. Of note, intrauterine growth restriction, being born small for gestational age, and low birth weight SD score, have all been previously associated with reductions in brain volumes within preterm infants^12,18,45^, including in our own recent work^46^. Thus, while the current study provides limited evidence for a relationship between being small for gestational age and brain regional growth independent of TBV growth, the long-term cerebral effects and associated clinical significance for growth restricted preterm infants warrant continued investigation^47^.

There were inconclusive associations between brain growth and either bronchopulmonary dysplasia or social risk, where no relationships were present both before and after TBV adjustment, despite the reported effects of these factors on brain development^17,19^. Increasing evidence suggests that while several individual risk factors may be important in prematurity, exposure to cumulative risk factors is associated with increased brain structural vulnerability;^48^ the long-term clinical significance of which remains to be fully described.

The current study found evidence of associations between larger brain size and higher IQ, similar to findings of a meta-analysis of >8000 children and adults^21^. We have previously demonstrated associations between IQ at 7 years and volumetric growth from infancy to 7 years in the same cohort^14,15^. Our current findings extend our earlier work to demonstrate associations between IQ at 13 years and more detailed regional brain growth over this protracted age range.

Regional brain growth associations with higher IQ, independent of overall brain growth, were more commonly observed between 7 and 13 years, and for healthy FT children only. These regions included the parietal lobe (superior parietal, supramarginal and inferior parietal), frontal lobe (precentral) and isthmus cingulate in the limbic system. These findings are consistent with the Parieto-Frontal Integration Theory^23^, and with our original hypothesis that parieto–frontal regions in particular would be associated with higher IQ. While it has been suspected that development of this parieto–frontal network is associated with IQ based on cross-sectional studies examining cortical gyrification^49,50^, until now it has not been demonstrated that growth of these regions is associated with IQ in a longitudinal study of brain development from infancy to adolescence. There was also evidence for an unintuitive association in the FT group, between poorer growth of the right pars opercularis region in the frontal lobe from 7 to 13 years and higher IQ at 13 years. This could be a result of the small sample size for the FT group, and potential false positive despite false discovery rate (FDR) correction.

Interestingly, in the current study, VP children’s volumetric growth between 7 and 13 years was not associated with IQ. Furthermore, the only associations between brain regional growth from term-equivalent to 7 years and IQ were discrepant between VP and FT children, and were not independent of TBV growth and social risk. Together these results suggest that the typical pattern of association between parieto–frontal volumetric growth and IQ may be disrupted by the environmental insult of VP birth. It may be that healthy microstructural development of the connective white matter between parieto–frontal regions is more imperative for optimising IQ in VP children than macrostructural volumetric growth. Congruent with this, we have shown that VP white matter connectivity in a network that included parieto–frontal regions was associated with IQ at 7 years in this cohort^51^. It was also interesting to note that for both groups in general, parieto–frontal regions were stable or decreased in volume between 7 and 13 years. Thus, despite the corresponding rapid growth period between term-equivalent and 7 years, the more subtle differences that occur between 7 and 13 years may be more clinically important, whereby individuals experiencing greater growth in particular regions during this period may be at a functional advantage. Research linking environmental and genetic factors with brain structure and cognitive function may shed further light on this complex interplay^52^.

A major strength of the current study is the unique longitudinal follow-up of infants from the early postnatal period through to early adolescence. Further, while the differences in brain tissue structure and contrast from infancy to childhood necessitate different segmentation approaches at term-equivalent and at 7 and 13 years, the brain parcellation scheme utilised for the neonatal time-point is comparable with that used for the 7- and 13-year time-points^9,10^. This study also has some limitations, some of which are inherent to longitudinal MRI investigations. For example, loss to follow-up meant that participants were not identical between time-points and our dataset contained missing data for some measures. Despite this, the mixed models we employed are relatively robust to missing data, which helps to mitigate potential bias due to drop out. Furthermore, technological advances over the course of the study meant that different acquisition software and hardware were used across time-points. Although this introduces variability that could possibly affect the results of this study, volumetric measures appear to be relatively robust to scanner differences^53^. In addition, the limited resolution of our neonatal scans, relative to the thickness of the cortex at this time-point, meant we were unable to investigate cortical thickness and surface area longitudinally. It is possible that group differences and associations may have been less likely to be identified for smaller brain regions due to inherent higher variability in volume measures. Another statistical limitation is that we modelled brain growth linearly from term-equivalent to 7, and 7 to 13 years. Given brain development is complex, growth trajectories are likely best described by nonlinear models^4^. However, our data were very highly clustered around just 3 time-points, therefore it was not appropriate to apply a more complex nonlinear model. Future studies should incorporate extra time-points in-between these critical developmental periods in case the lack of group differences from 7 to 13 years was due to the nonlinear nature of brain development. Moreover, continued research focusing on the earlier years of life when neurodevelopment is most rapid is required to more fully characterise brain growth trajectories during this important period when deviations from typical maturational trajectories may have greater clinical implications.

In summary, this study provides insight into typical regional brain development for 68 cortical and 14 subcortical brain regions in a serial study from infancy to adolescence. We also report the delayed or disrupted growth trajectory of multiple brain regions for VP compared with FT children over the first 7 years of life. Perinatal brain abnormality in VP children was a factor associated with slower growth from term-equivalent to 7 years. Consistent with the rapid brain growth and development known to occur in the first few years of life, the period from infancy to early childhood appeared to be a vulnerable stage for preterm children, reflecting a potentially critical window of opportunity during which clinical interventions may have the greatest potential impact. From 7 to 13 years, most brain growth in both groups ceased or declined in a region-specific manner, apart from subcortical regions and the pericalcarine region in the occipital cortex that continued to increase in volume. Of note, VP infants demonstrated overall reduced brain size relative to their FT peers by 13 years, suggesting a lack of ‘catch-up’ growth and potential long-lasting structural brain alterations. Importantly, this study provided new insights into the functional significance of regional brain growth over this period, describing associations between greater growth of the parieto–frontal network during middle childhood and better IQ at 13 years.

## Methods

### Participants

Participants were recruited during the neonatal period from the Royal Women’s Hospital in Melbourne, Australia, as part of the Victorian Infant Brain Study (VIBeS) prospective longitudinal cohort between 2001 and 2004. The VP group consisted of 224 surviving children born <30 weeks’ gestation or very low birthweight (<1250 g) who did not have genetic or congenital abnormalities. The FT control group comprised 45 infants born ≥37 and ≤41 weeks’ gestation. The study was approved by the Human Research and Ethics Committees of the Royal Women’s Hospital and the Royal Children’s Hospital, Melbourne. Parents gave written informed consent for their child to participate.

At age 7 years corrected for prematurity, 197 VP and 42 FT children were followed up, and at 13 years’ corrected age, 179 VP and 34 FT children were followed up. The main reasons for loss to follow-up included families declining or withdrawing from the study, living in other countries, or being unable to be contacted.

### MRI

All brain MRI scans were acquired at the Royal Children’s Hospital, Melbourne. At term-equivalent age (38–42 weeks’ postmenstrual age), 223 VP and 45 FT infants were scanned without sedation. Structural *T*_2_-weighted (1.7–3.0 mm coronal slices; repetition time 4000 ms; echo time 60/160 ms; flip angle 90°; field of view 220 × 160 mm; matrix 256 × 192, interpolated 512 × 512) images were acquired on a 1.5 Tesla General Electric MRI scanner (Signa LX Echospeed System; General Electric, Milwaukee, WI).

At 7 years’ corrected age, 159 VP and 35 FT children were scanned without sedation using a 3 Tesla Trio Siemens MRI scanner (Siemens, Erlangen, Germany). *T*_1_-weighted (0.85 mm sagittal slices, repetition time 1900 ms, echo time 2.27 ms, flip angle 9°, field of view 210 × 210 mm, matrix 256 × 256) images were obtained.

At 13 years’ corrected age, 141 VP and 29 FT children were similarly scanned on the 3 Tesla Trio Siemens MRI scanner. *T*_1_-weighted sequences (0.9 mm^3^ sagittal slices, repetition time 2530 ms, echo times 1.77, 3.51, 5.32, 7.2 ms, flip angle 7°, field of view 230 × 209 mm, matrix 256 × 230, interpolated 256 × 256) were obtained.

### Image pre-processing and brain volumetry

At term-equivalent age, images were bias-corrected and brain extracted^54^. For labelling, each of the *T*_2_-weighted and segmentation images from the M-CRIB atlas^10^ were registered to each *T*_2_-weighted image in the current sample using ANTS^55^. pSTAPLE^56^ was then used to apply the M-CRIB labels to each brain.

At 7 and 13 years, surface-based brain parcellation was performed on *T*_1_ images using FreeSurfer 6.0^57^, with manual editing according to FreeSurfer guidelines. Brainstem and hippocampal volumes were generated using FreeSurfer’s subfields tools^58,59^. ICV, TBV and CSF volume were obtained using Statistical Parametric Mapping version 12 (http://www.fil.ion.ucl.ac.uk/spm/). Extra-axial CSF volume was generated by subtracting FreeSurfer’s ventricular volumes from the SPM-derived CSF volume.

There were 68 cortical and 14 subcortical brain regional volumes that were comparable across the three time-points, which make up the outcomes of interest.

### Perinatal and social factors

Data collected on perinatal factors included: sex; moderate-to-severe brain abnormality qualitatively assessed on term-equivalent MRI using an established scoring system, which equated moderate-to-severe brain abnormality to patterns of qualitative abnormalities such as bilateral punctate white matter signal abnormalities or unilateral significant parenchymal or cerebellar injury^25^; bronchopulmonary dysplasia, defined as requirement for supplemental oxygen at 36 weeks; and small for gestational age, defined as birthweight more than two SDs below the mean weight relative to that expected for sex and gestational age, according to the British Growth Reference dataset^60^. Social risk was calculated when the child was 2 years of age using a score of 0–2 for family structure, primary caregiver education, primary income earner employment and occupation, language spoken at home and maternal age at birth, with an overall score from 0 to 12^61^. Social risk was categorised as higher for scores ≥2, and lower for scores <2.

### Intelligence at 13 years

As part of the follow-up assessment at age 13 years, the composite standard score from the Kaufman Brief Intelligence Test, Second Edition was administered (mean = 100, SD = 15) as a measure of IQ^62^. Social risk at 13 years was calculated using a score of 0–2 for family structure, primary caregiver education, primary income earner employment and occupation, language spoken at home, and maternal age at birth, with an overall score from 0 to 12^61^. Social risk was categorised as higher for scores ≥2, and lower for scores <2.

### Statistical analyses

Analyses were conducted using Stata 15.0 (StataCorp, TX), and results were displayed on brain surfaces using Matlab (MathWorks, MA). Volumetric variables were standardised relative to the mean and SD of the brain region prior to statistical analyses. Thus, beta coefficients (β) reported in the results are standardised.

For aim 1, separate linear mixed effects models were used to examine brain growth trajectories for each cortical and white matter region of interest, applied to data from all VP and FT children from term-equivalent to 13 years. Group (VP or FT) and sex (male or female) were included as fixed effects with a random intercept to allow for the repeated observations within participants at the different assessment time-points. Time was included as a 3-level variable (0 (term-equivalent), 7 and 13 years). Although age is strictly continuous, age was treated as discrete due to the narrow range of observation times at each follow-up compared with the intervals between the time-points. Interactions between group and time and between sex and time were included to investigate differences in trajectories from term-equivalent to 7 and 7 to 13 years between birth groups (VP or FT) and sexes. A three-way interaction term was included in each model to determine whether any group differences in the growth trajectories varied by sex. All *p* values were corrected for multiple comparisons according to the number of brain regions investigated, using the FDR based on Benjamini and Yekutieli (first method)^63^. As a secondary analysis, all models were repeated adjusted for TBV to account for potential inter-subject variability in head size, and to determine whether growth of particular regions was specifically vulnerable in a particular birth group or sex, over and above global brain growth.

To evaluate the relationship between perinatal factors, social risk and brain growth (aim 2), separate linear mixed effects models were fitted to each brain volume including the variable of interest (moderate-to-severe brain abnormality, bronchopulmonary dysplasia, small for gestational age, and higher social risk at 2 years) and time (0, 7 and 13 years) as fixed effects, as well as an interaction between each variable of interest and time applied to the VP group only. Models included a random intercept to capture correlations between observations within participants at the different assessment time-points. All *p* values were FDR-corrected and secondary analyses were adjusted for TBV.

Finally, associations between brain growth rates and 13-year IQ (aim 3) were assessed using separate linear regression models for each volume growth rate-IQ combination. Growth rate from term-equivalent to 7 years was calculated as: (volume at 7 years, mm^3^—volume at term-equivalent, mm^3^)/(age at 7-year MRI, year—age at term-equivalent MRI, year). Growth rate from 7 to 13 years was calculated as: (volume at 13 years, mm^3^—volume at 7 years, mm^3^)/(age at 13-year MRI, year—age at 7 year MRI, year). A group-by-growth rate interaction term was included to allow the effect of growth rate on IQ to vary by group. If the relationship varied by group (interaction *p* < 0.05) then the relationships are presented separately for each group from the model including the interaction; if the relationship did not vary by group (interaction *p* ≥ 0.05) then the relationships are reported for the combined groups (VP and FT children). All estimates were adjusted for sex and age at assessment of IQ, and secondary analyses adjusted for the growth in total functional brain tissue (TBV growth rate calculated similarly to the regional growth rates) and social risk at 13 years, since these covariates are likely to relate to the dependent variable, IQ at 13 years.

### Reporting summary

Further information on research design is available in the Nature Research Reporting Summary linked to this article.

## Supplementary information

Supplementary Information

Reporting Summary

## Data Availability

The datasets generated during and/or analysed during the current study are available from the corresponding author on reasonable request. The MRI data are not publicly available due to ethical restrictions.
