# Supplementary material for: Tracking regional brain growth up to age 13 in children born term and very preterm
Source: Nat Commun. 2020 Feb 4;11:696. doi: 10.1038/s41467-020-14334-9 (PMC7000691; doi:10.1038/s41467-020-14334-9)
Supplement: Supplementary file 1 — Supplementary Information [file 41467_2020_14334_MOESM1_ESM.pdf]

a

Whole brain, cortex, white matter, and cerebrospinal fluid

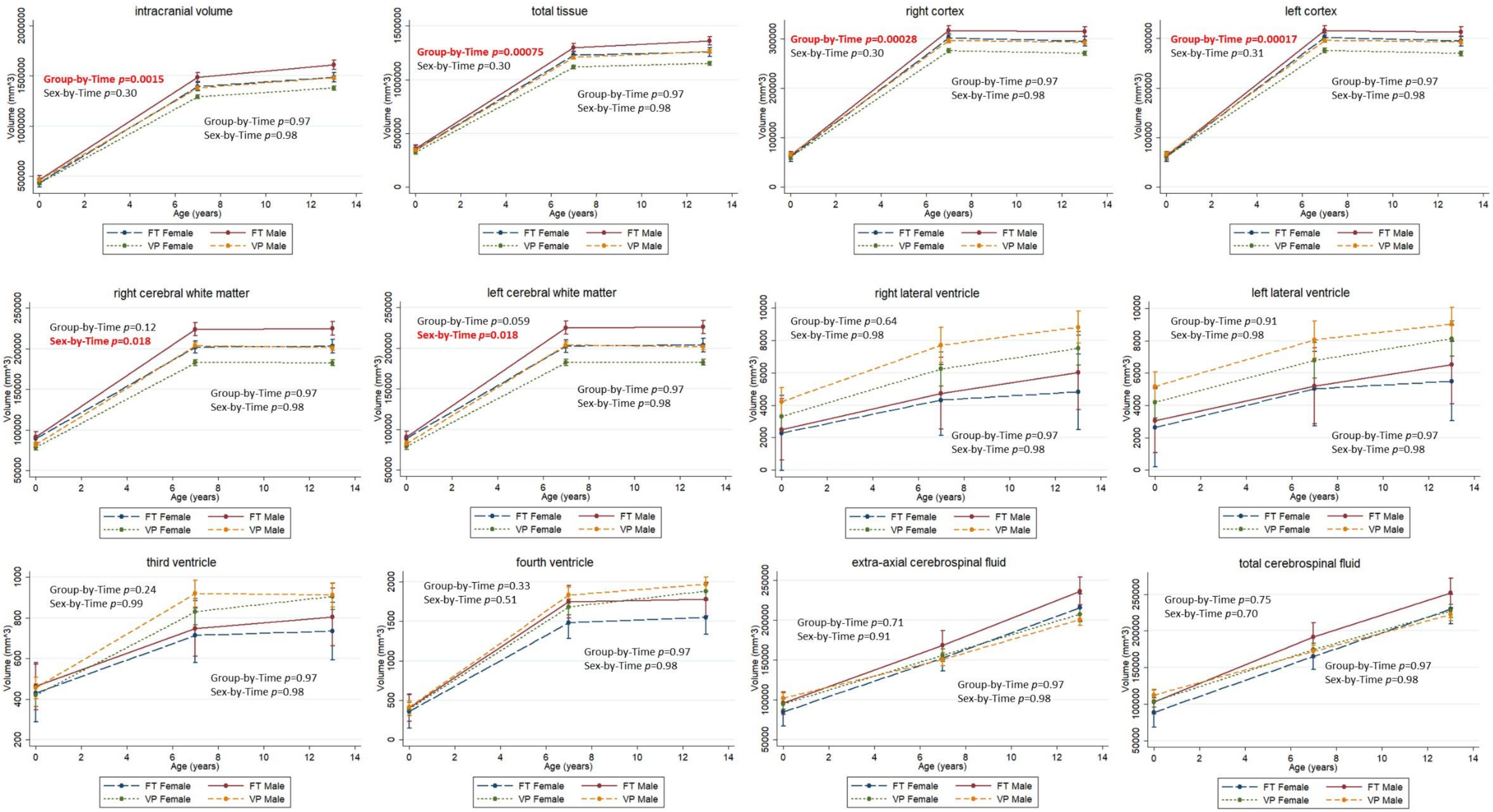

b

Corpus callosum, brainstem, cerebellum and insula

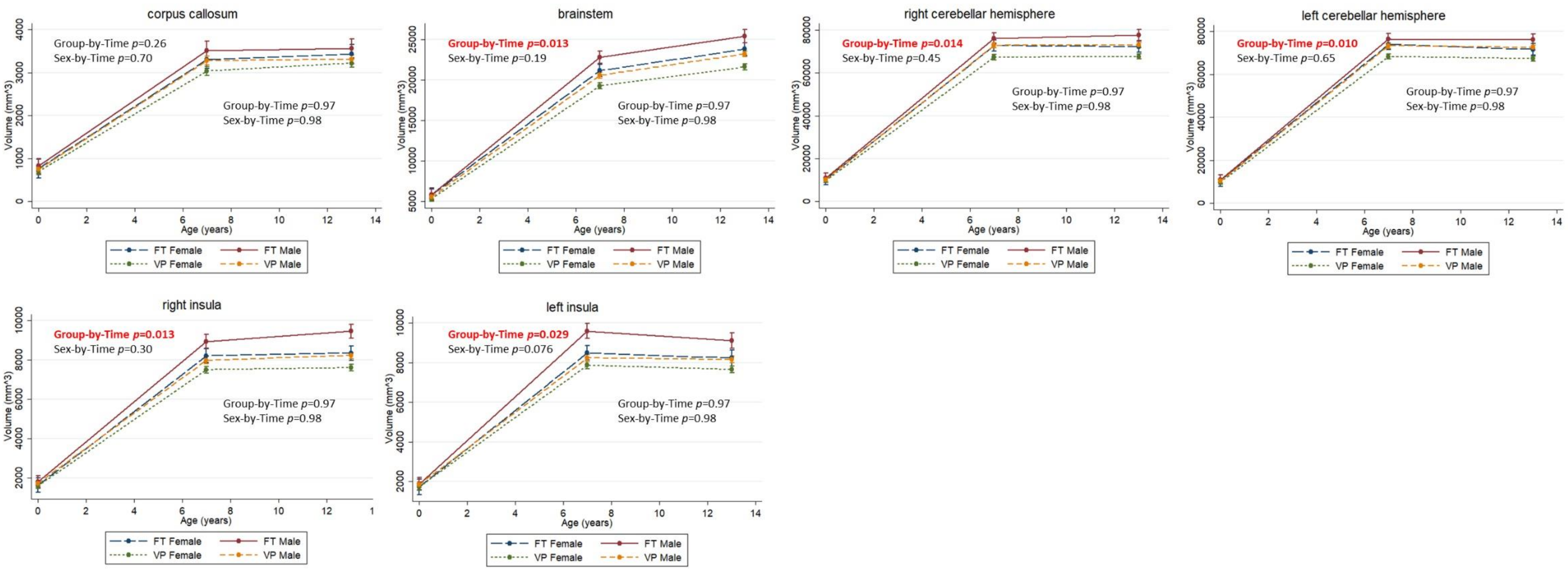

c

Basal Ganglia and thalamus

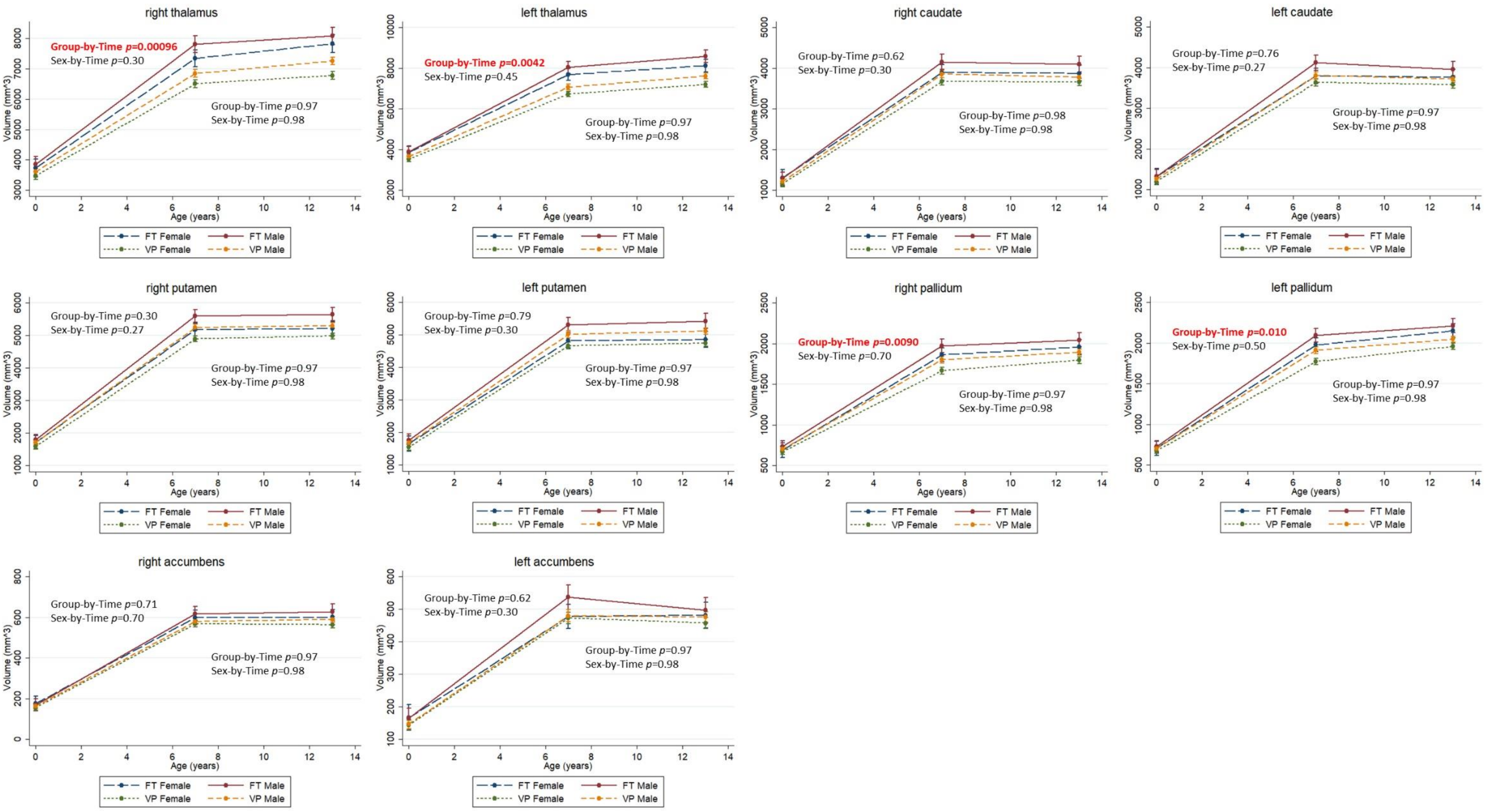

d

Limbic system

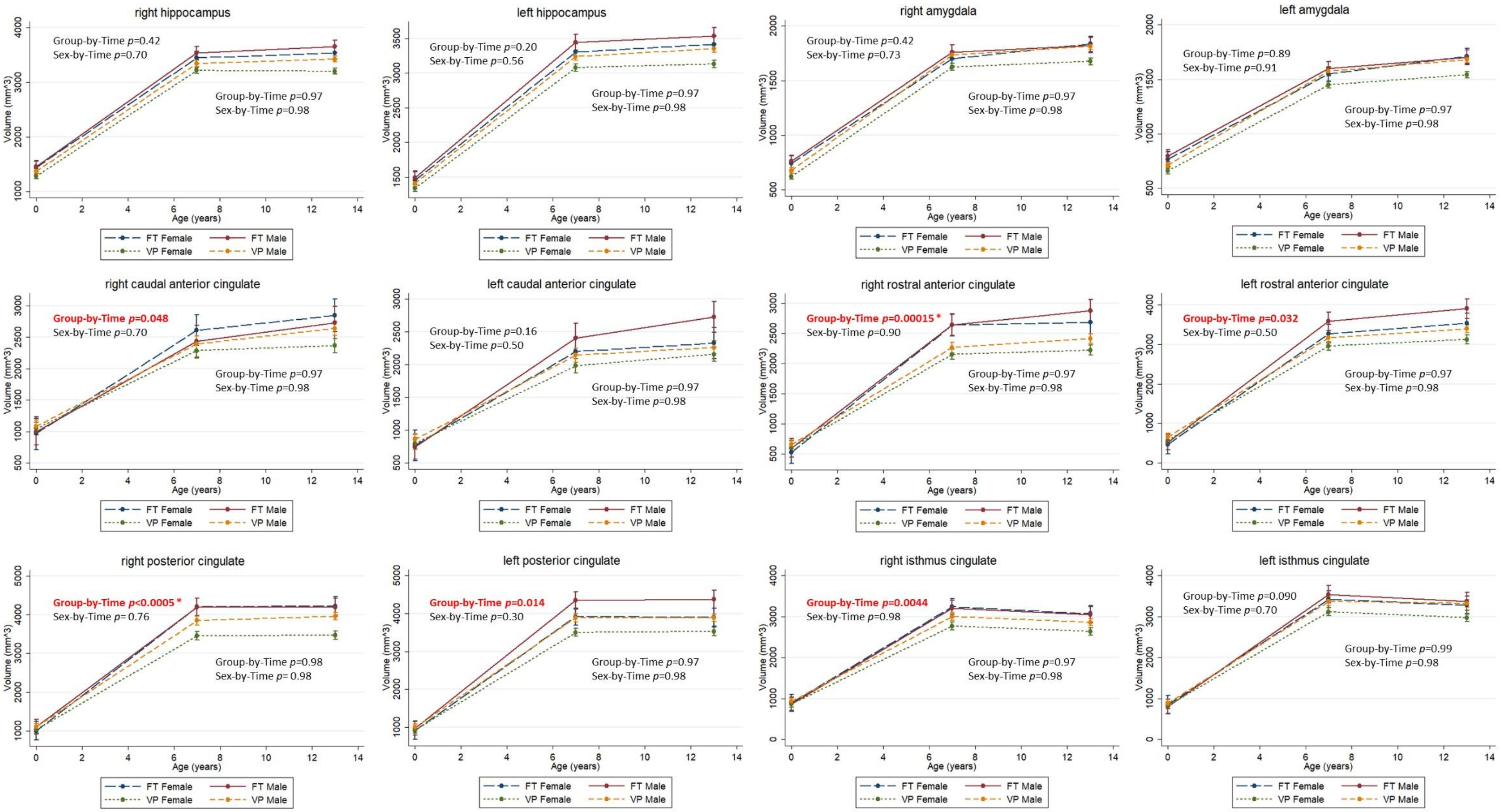

e **Temporal lobe (medial aspect)**

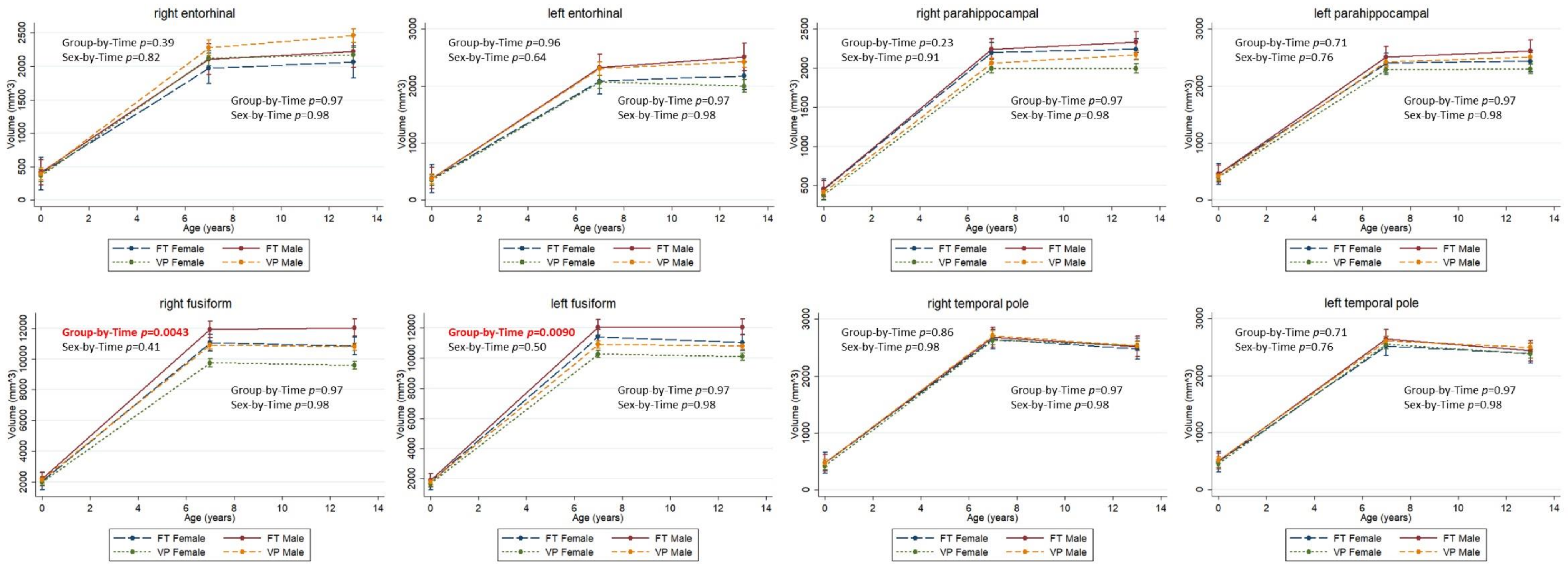

f

Temporal lobe (lateral aspect)

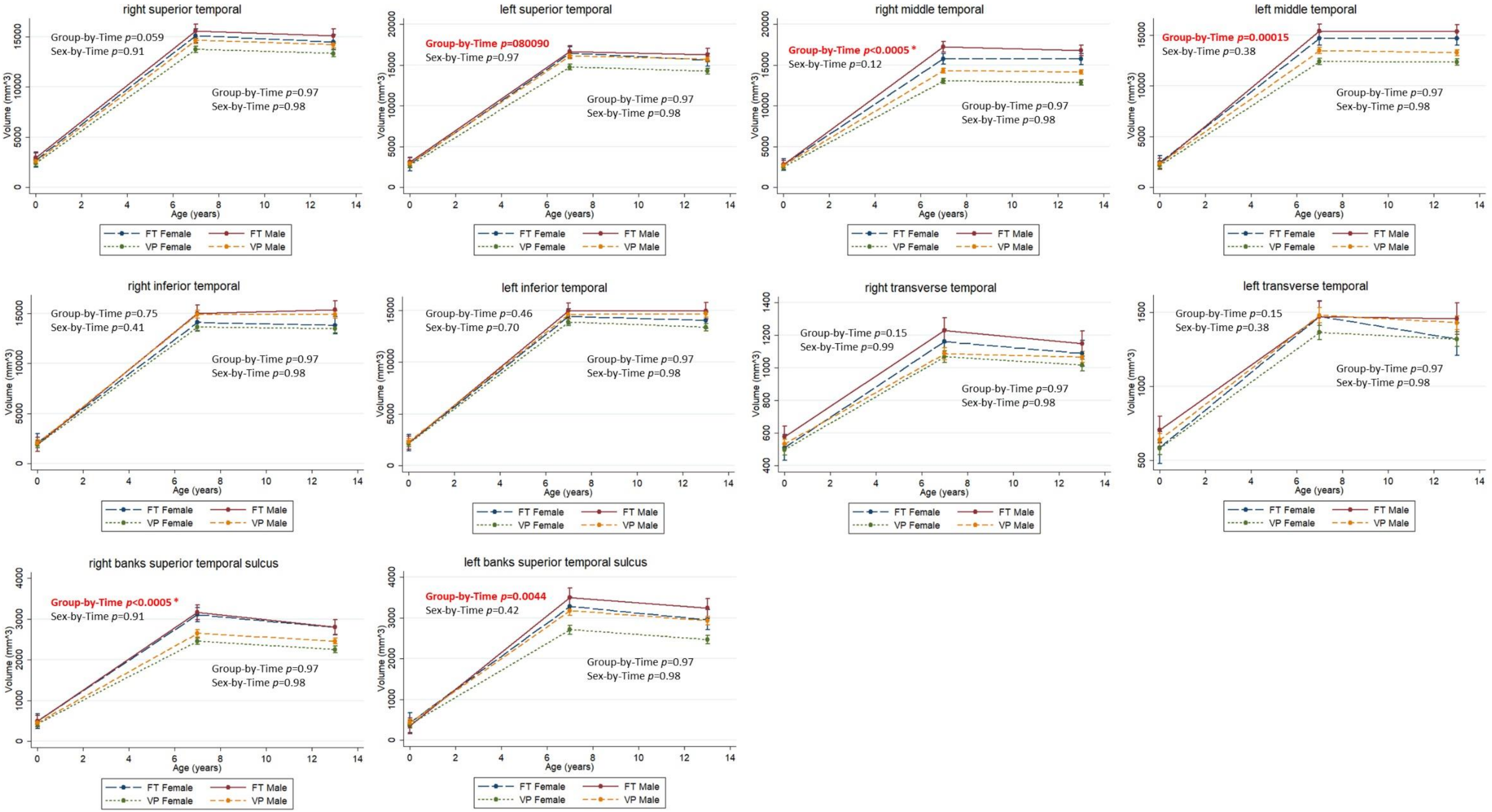

g

Frontal lobe

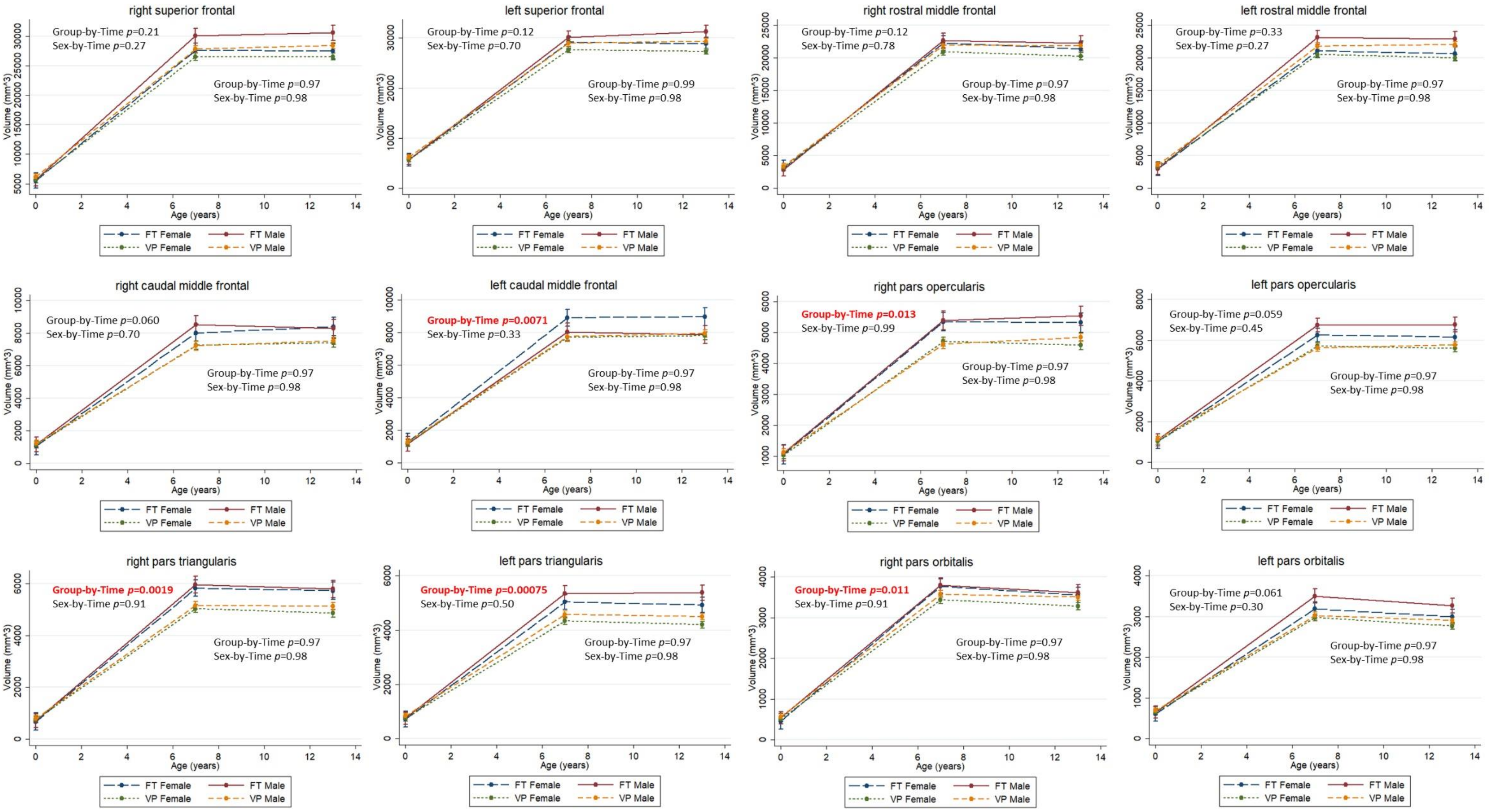

h

Frontal lobe (continued)

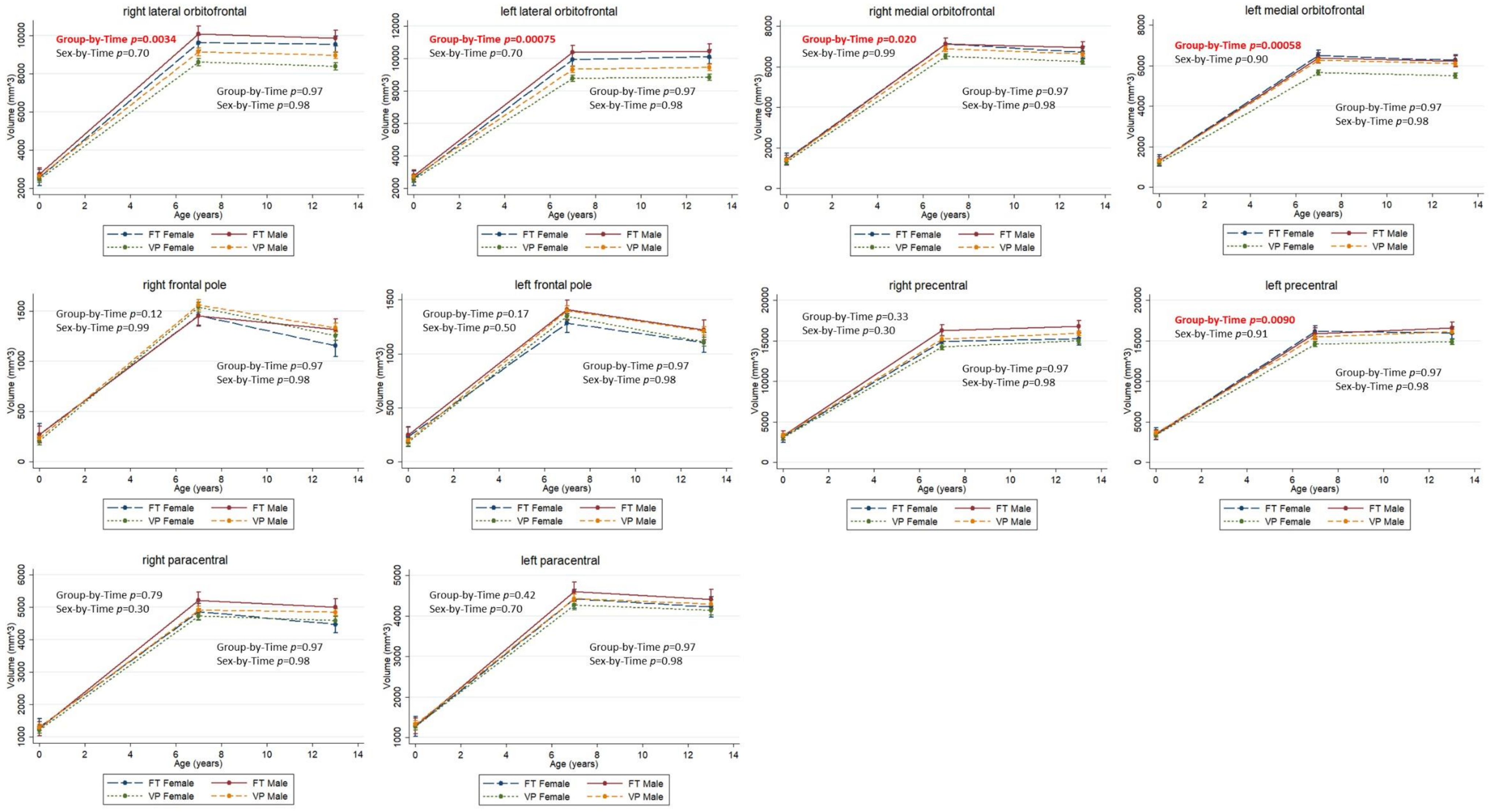

i

Parietal lobe

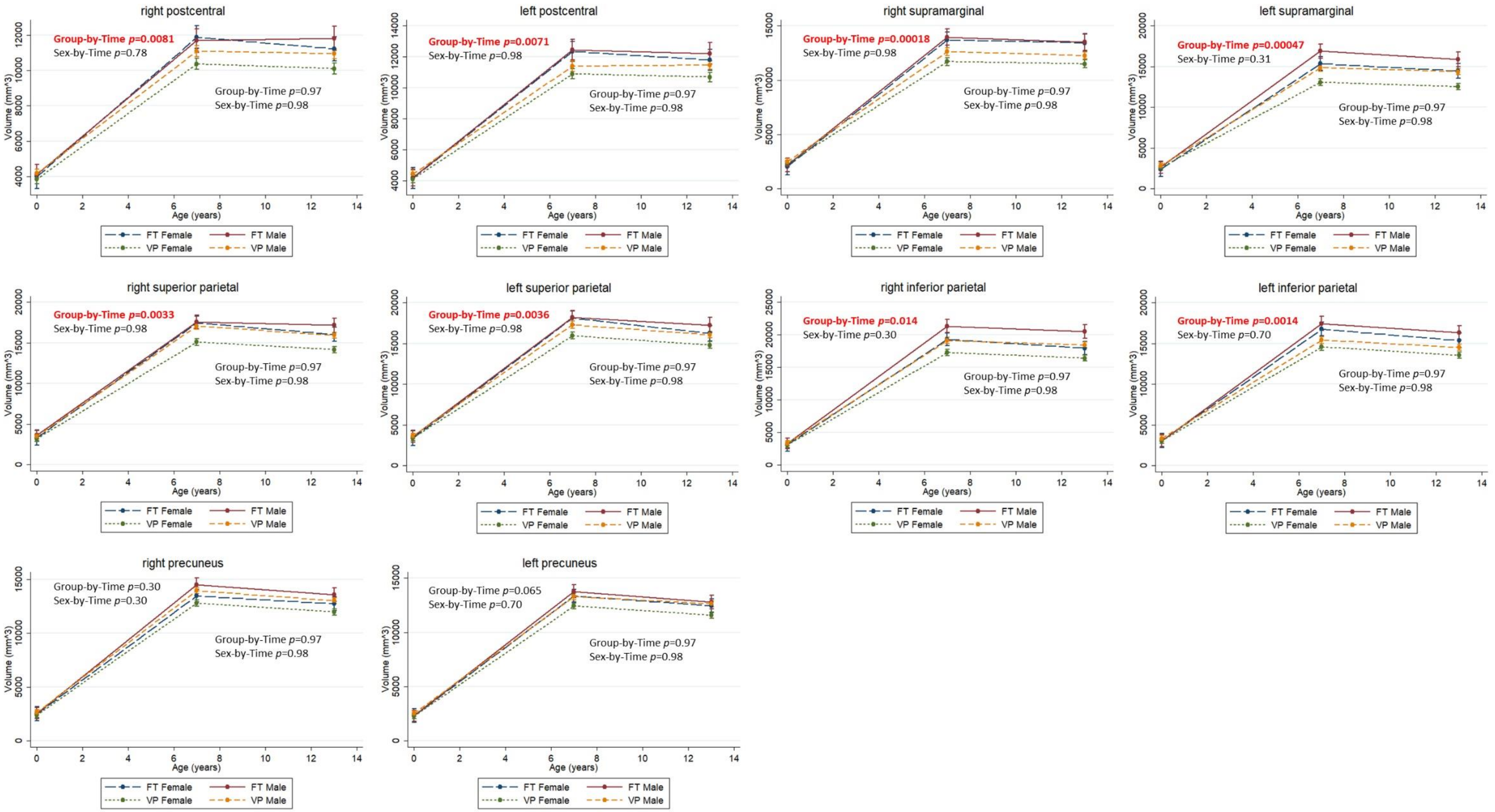

j **Occipital lobe**

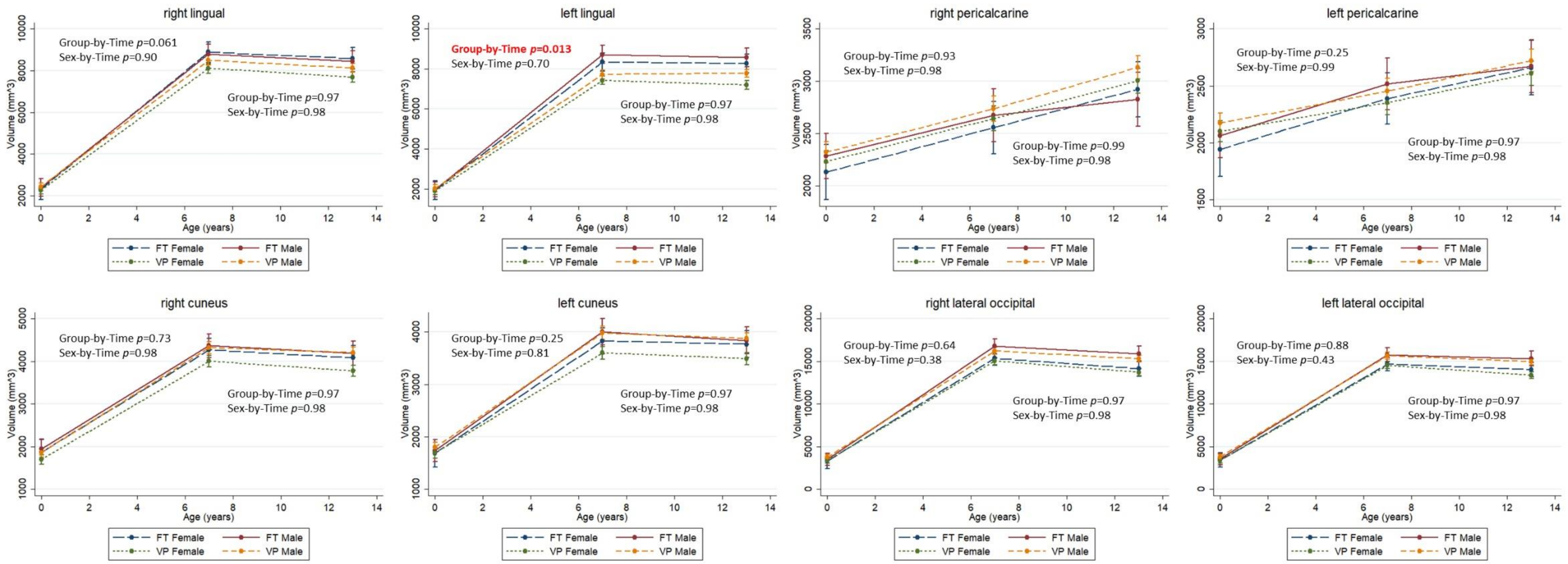

Supplementary Figure 1. Trajectory of growth for brain regions between term-equivalent age, 7 years and 13 years of age separated for birth group [very preterm (VP) and full-term FT)] and sex (male and female). NOTE: false discovery rate corrected *p*-values for the group and sex interactions from linear mixed effects models are listed for volumetric change from term to 7 and 7 to 13 years separately; Interactions where there was evidence of a group or sex difference ($p<0.05$) before adjusting for total brain volume are denoted with red text; Interactions where there was evidence of a group or sex difference ($p<0.05$) after adjusting for total brain volume are denoted with a star.

a **Whole brain, cortex, white matter, and cerebrospinal fluid**

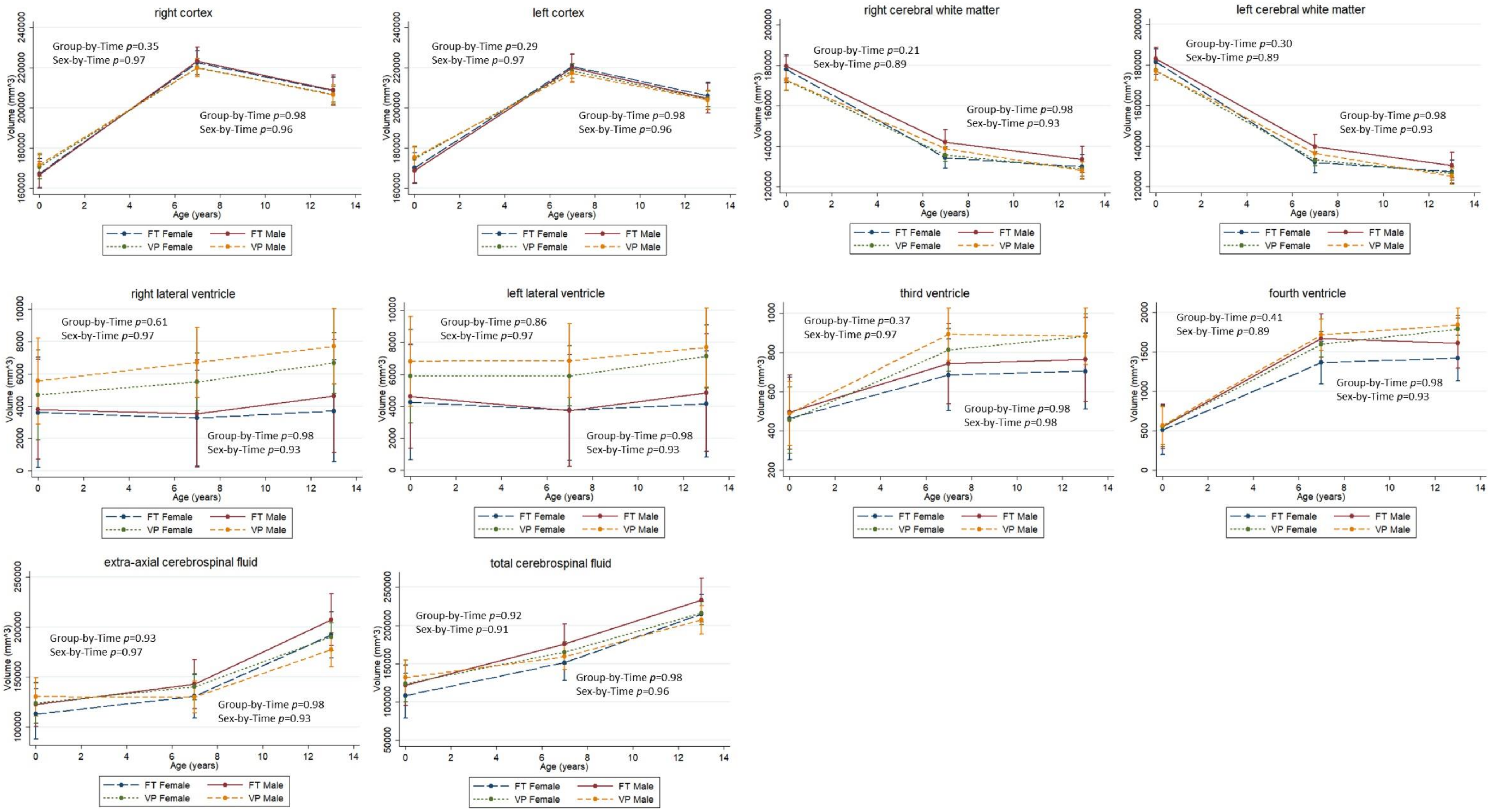

b

Corpus callosum, brainstem, cerebellum and insula

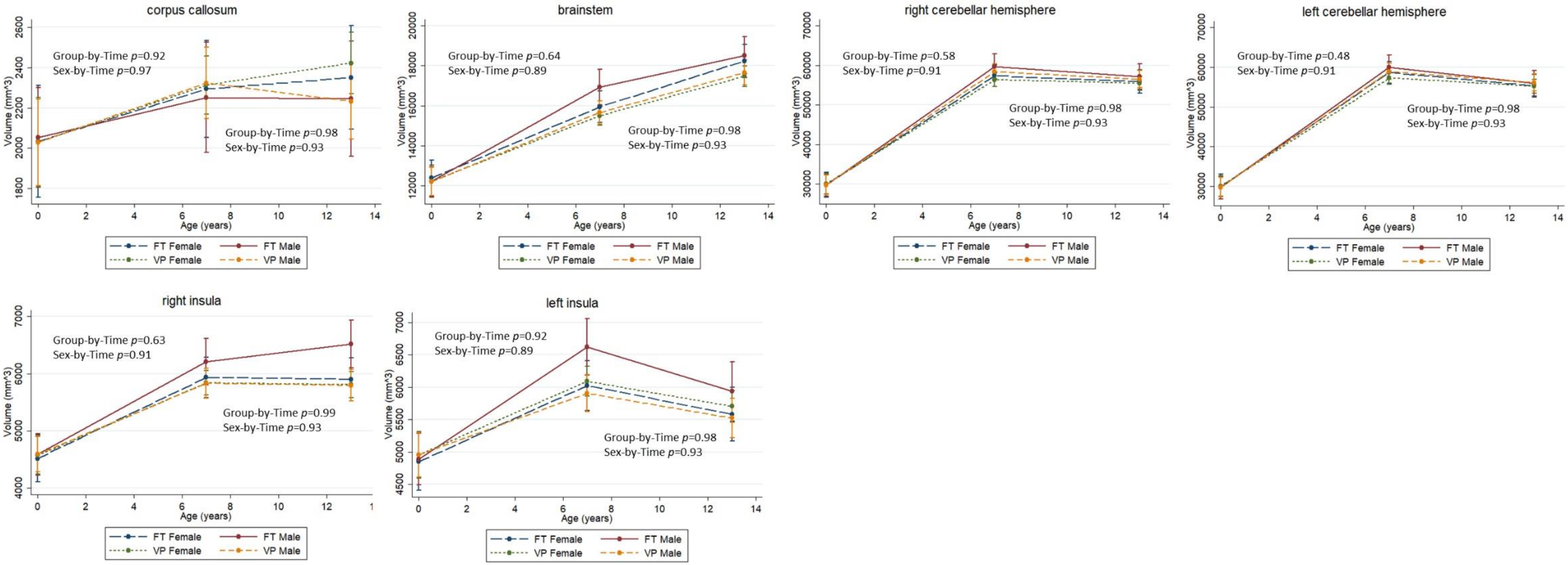

c **Basal Ganglia and thalamus**

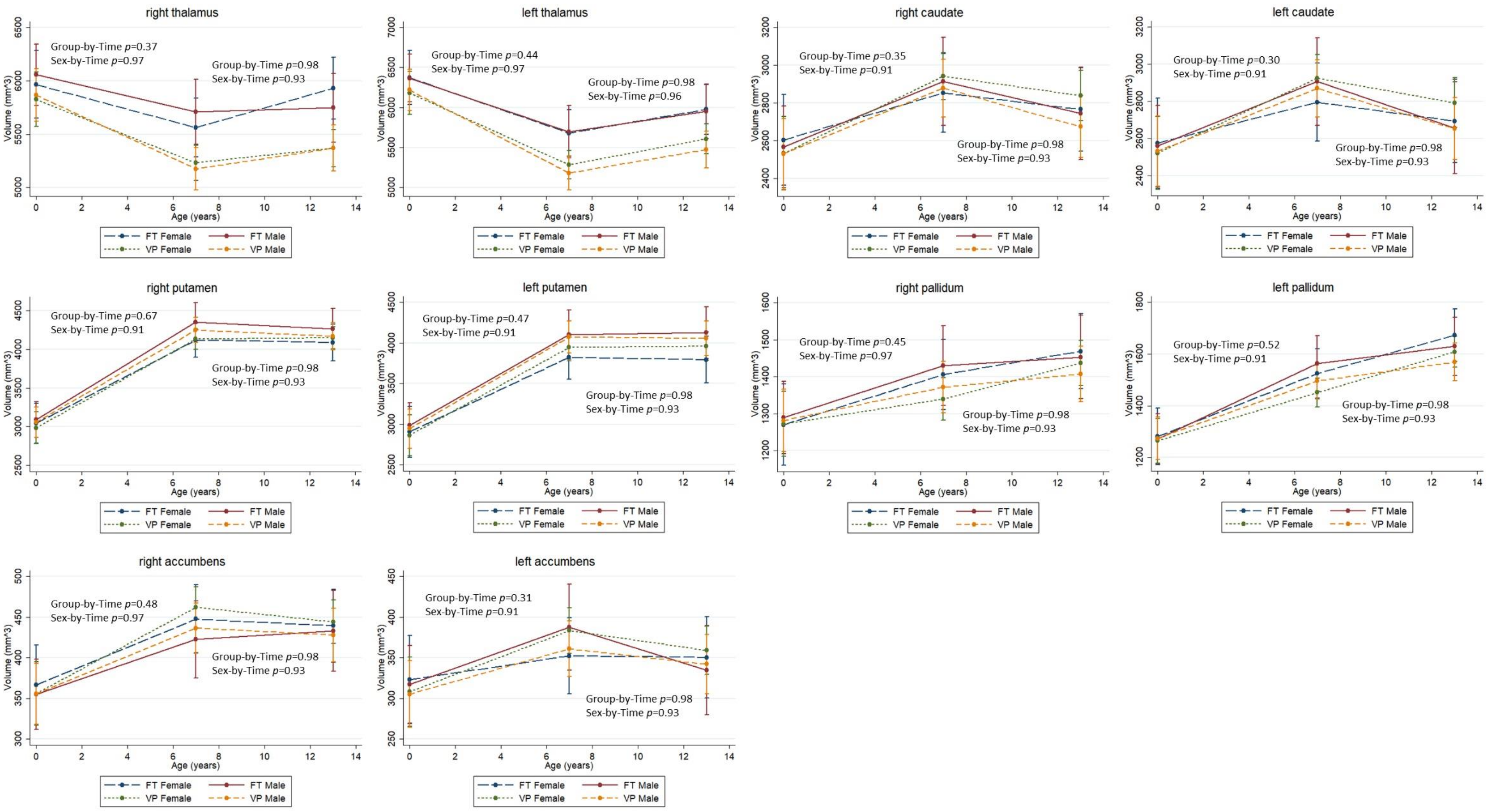

d **Limbic system**

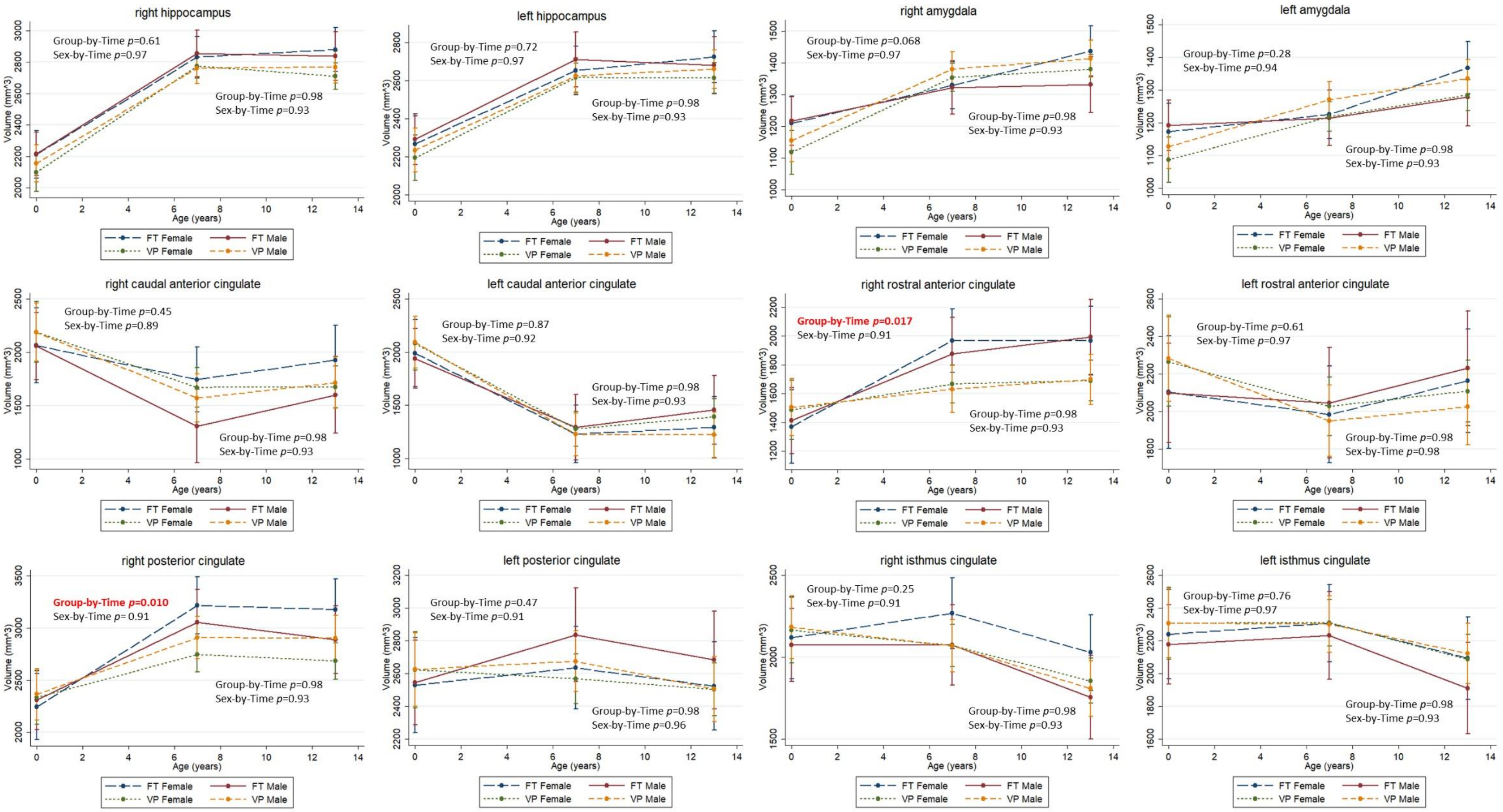

e **Temporal lobe (medial aspect)**

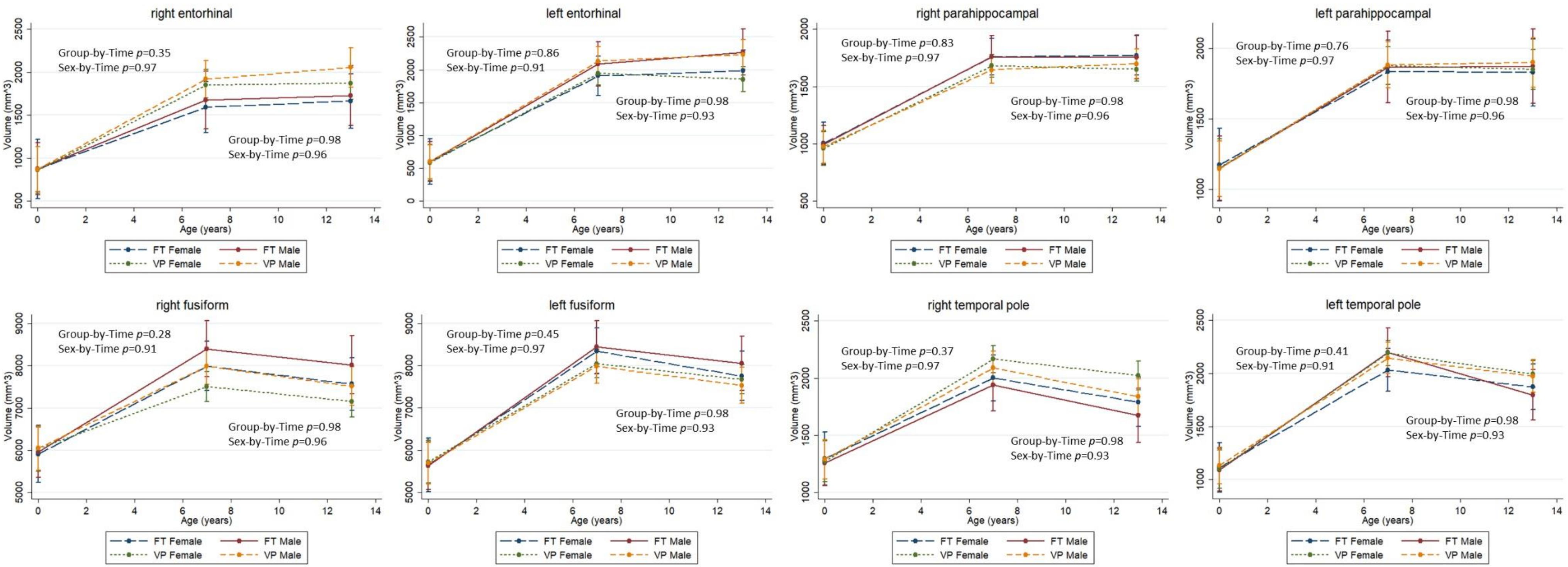

f

Temporal lobe (lateral aspect)

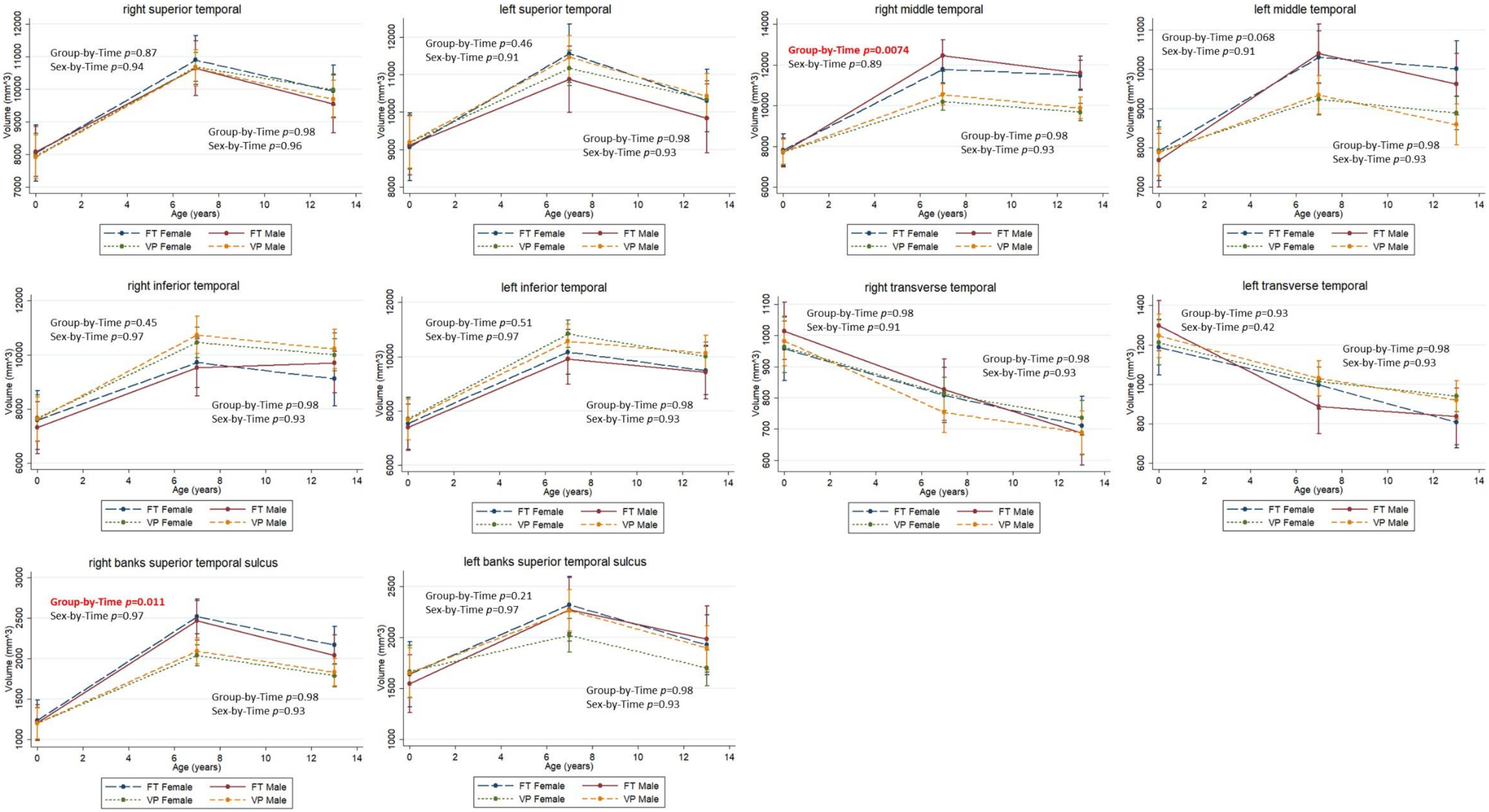

g

Frontal lobe

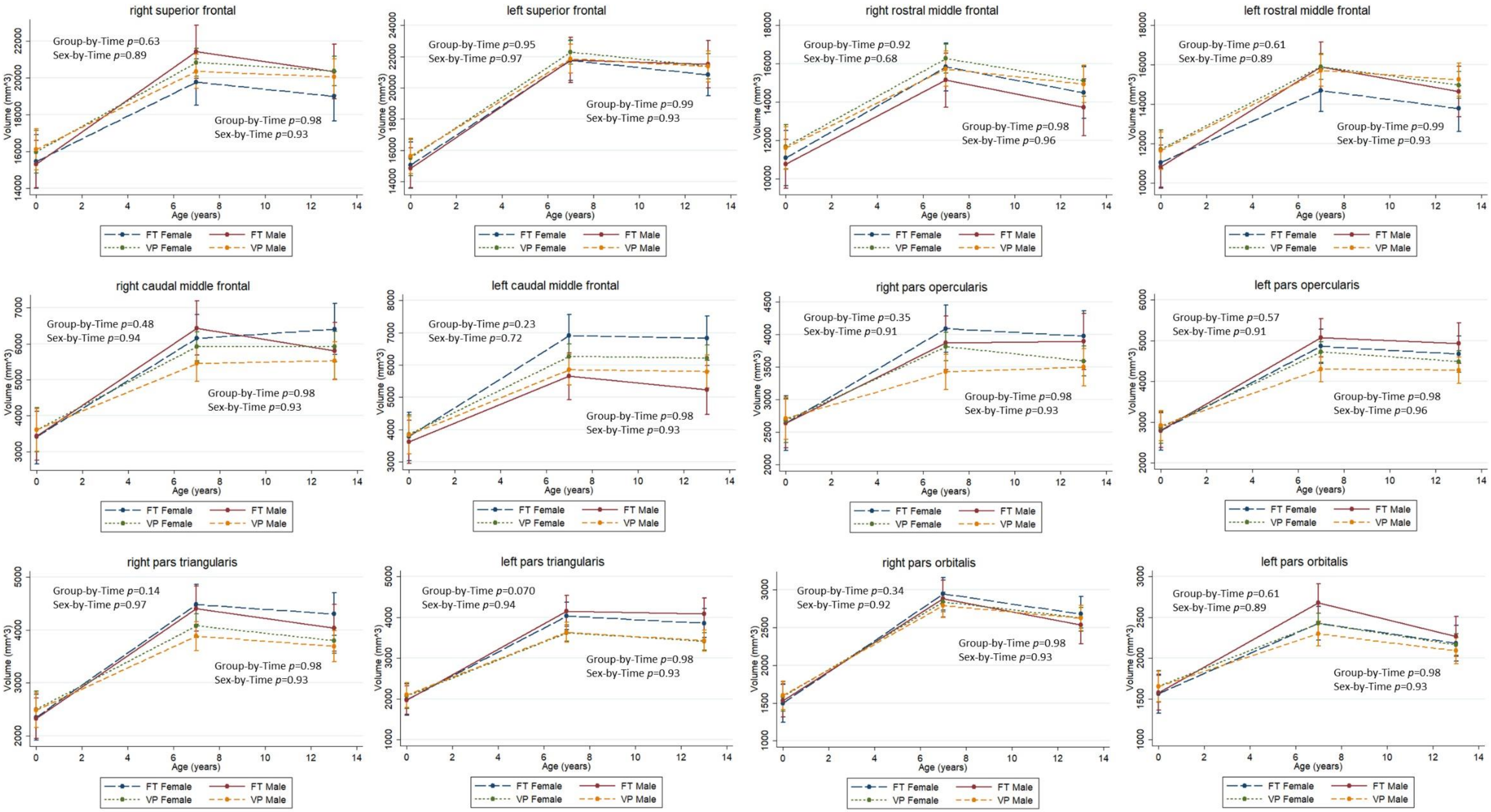

h **Frontal lobe (continued)**

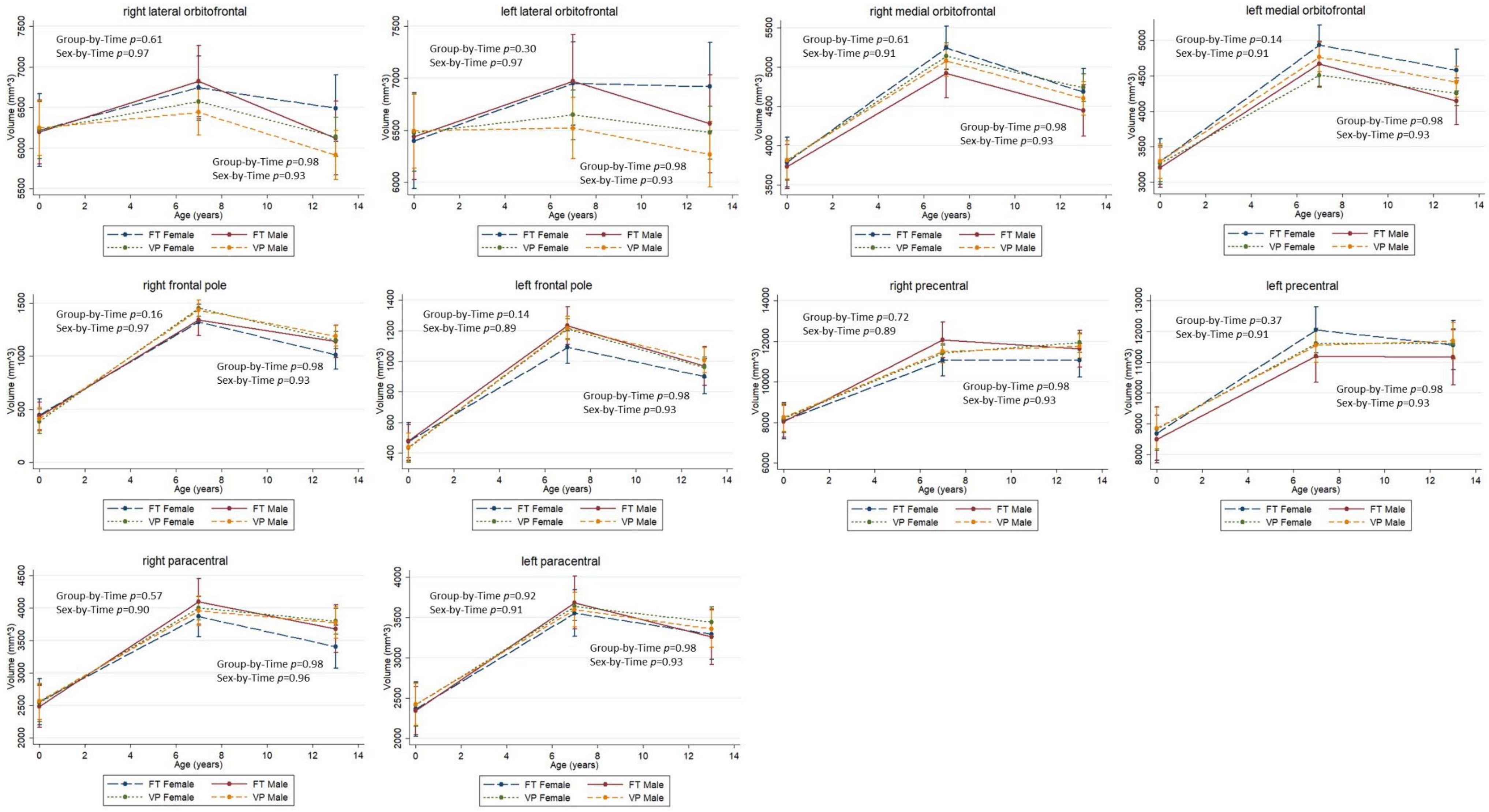

i

Parietal lobe

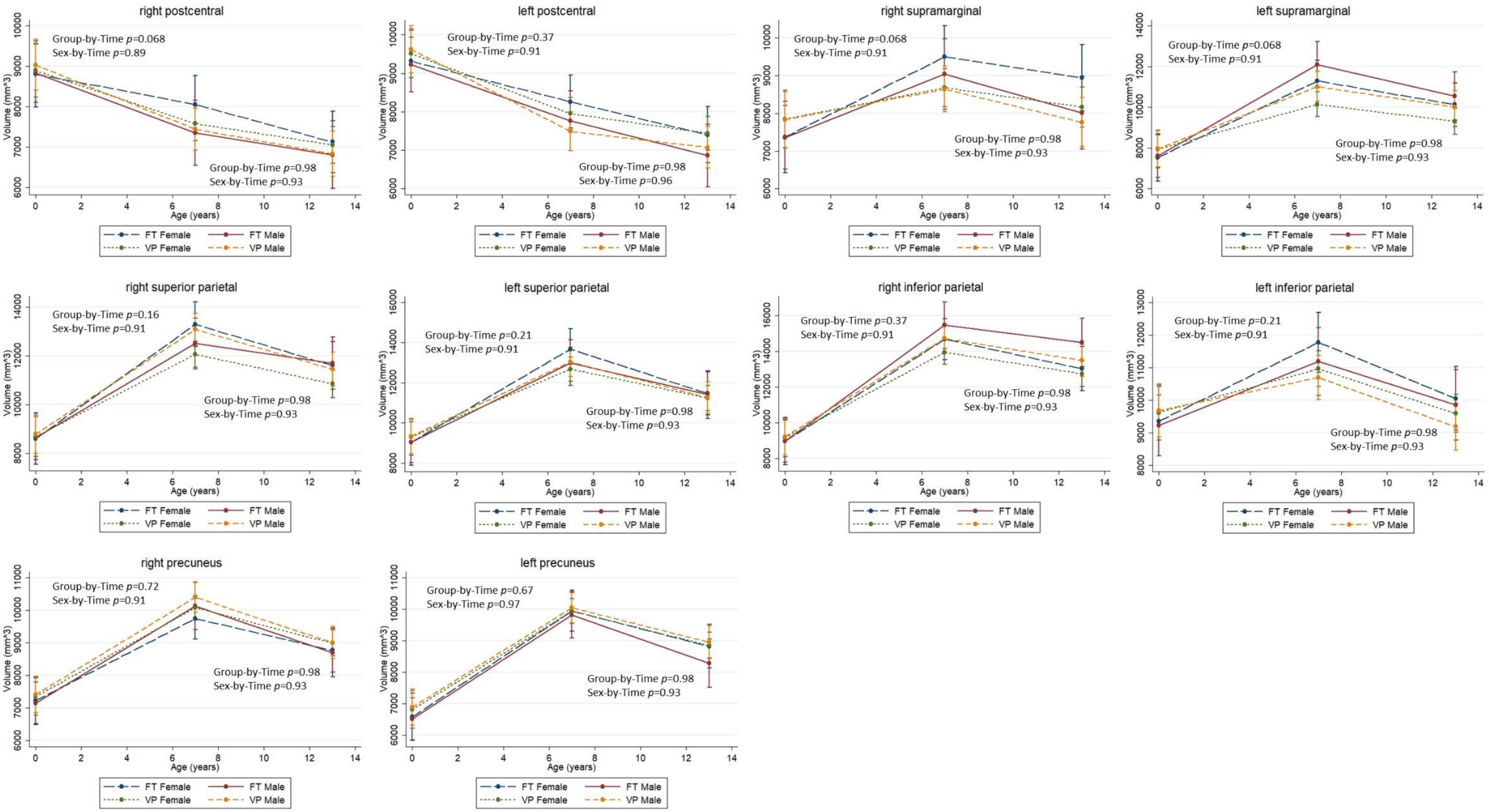

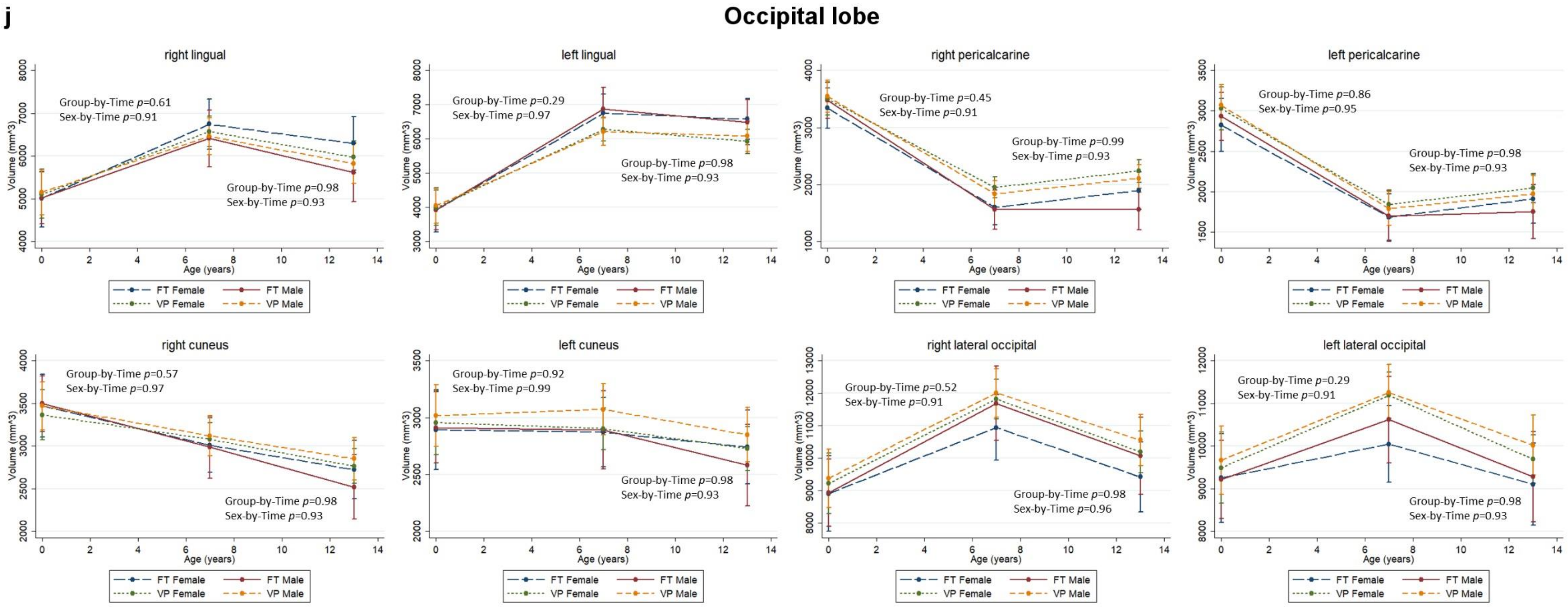

Supplementary Figure 2. Trajectory of growth for brain regions between term-equivalent age, 7 years and 13 years of age separated for birth group [very preterm (VP) and full-term FT)] and sex (male and female) after adjusting for total brain volume. NOTE: false discovery rate corrected *p*-values for the group and sex interactions from linear mixed effects models are listed for volumetric change from term to 7 and 7 to 13 years separately; Interactions where there was evidence of a group or sex difference ($p<0.05$) are denoted with red text.

Supplementary Table 1a. Raw mean volumes (mm^3) per brain region for the very preterm (VP) and full-term (FT) groups, and mean differences in volumes between groups, at the neonatal time point.

Region	VP mean	VP SD	FT mean	FT SD	Mean difference	95% CI Lower	95% CI Upper	*p*-value (VP vs FT)
Intracranial	442155.11	60658.50	455981.52	51847.36	-13826.41	-35614.66	7961.84	0.212
Total brain tissue	333999.38	44008.60	357664.48	41785.22	-23665.10	-39677.75	-7652.45	0.004
Extra-axial cerebrospinal fluid	98988.07	20401.38	92094.50	12112.19	6893.57	-219.81	14006.95	0.057
Cerebrospinal fluid	108155.74	22838.00	98317.05	13136.02	9838.69	1889.71	17787.68	0.016
Total cortical grey matter	127565.62	20786.14	128303.31	21388.75	-737.68	-8388.80	6913.43	0.849
Left cortical grey matter	64076.66	10401.81	63706.67	10752.84	370.00	-3461.51	4201.50	0.849
Right cortical grey matter	63488.96	10486.61	64596.64	10773.19	-1107.68	-4966.71	2751.35	0.572
Left lateral ventricle	4663.29	3868.95	2932.77	1118.66	1730.52	411.25	3049.80	0.010
Right lateral ventricle	3666.39	3815.33	2457.29	1021.94	1209.11	-90.58	2508.79	0.068
Third ventricle	433.23	140.55	452.49	109.29	-19.26	-69.25	30.74	0.449
Fourth ventricle	404.75	91.92	380.00	72.04	24.75	-7.97	57.47	0.138
Left white matter	80986.59	11771.52	90567.86	10963.50	-9581.27	-13853.50	-5309.05	<0.001
Right white matter	80498.85	11636.28	90730.25	10772.58	-10231.40	-14451.26	-6011.53	<0.001
Total white matter	161485.44	23363.01	181298.11	21704.98	-19812.67	-28289.03	-11336.31	<0.001
Corpus callosum	731.98	127.00	805.33	102.23	-73.35	-118.68	-28.02	0.002
Brainstem	5475.24	572.50	5890.99	616.08	-415.75	-628.00	-203.51	<0.001
Left cerebellum	10112.24	1568.32	10697.63	1541.20	-585.39	-1158.74	-12.03	0.045
Right cerebellum	10065.56	1539.29	10667.74	1538.44	-602.18	-1166.30	-38.06	0.037
Left thalamus	3595.68	436.11	3865.67	392.22	-269.99	-427.57	-112.41	0.001

Left caudate	1231.80	173.34	1328.78	166.67	-96.98	-160.15	-33.80	0.003
Left putamen	1612.64	234.06	1707.66	241.06	-95.02	-181.18	-8.85	0.031
Left pallidum	690.98	90.51	725.37	89.53	-34.39	-67.51	-1.27	0.042
Left hippocampus	1373.49	160.90	1482.24	166.78	-108.75	-168.05	-49.46	<0.001
Left amygdala	690.25	99.17	782.42	107.27	-92.17	-128.96	-55.37	<0.001
Left accumbens	144.96	24.71	165.20	31.44	-20.24	-29.70	-10.78	<0.001
Right thalamus	3549.23	434.90	3834.72	373.73	-285.48	-441.79	-129.18	<0.001
Right caudate	1188.53	170.28	1288.35	158.07	-99.82	-161.59	-38.04	0.002
Right putamen	1653.05	229.73	1735.04	221.61	-81.99	-165.76	1.78	0.055
Right pallidum	689.77	87.95	723.33	97.57	-33.56	-66.34	-0.79	0.045
Right hippocampus	1328.43	154.98	1445.51	153.61	-117.08	-173.81	-60.35	<0.001
Right amygdala	653.87	97.05	743.54	94.18	-89.66	-125.08	-54.25	<0.001
Right accumbens	160.62	30.06	173.55	34.59	-12.94	-24.21	-1.66	0.025
Left banks superior temporal sulcus	414.11	117.32	402.77	118.47	11.34	-31.72	54.40	0.604
Left caudal anterior cingulate	824.02	163.51	763.25	144.06	60.77	1.83	119.71	0.043
Left caudal middle frontal	1228.67	256.79	1127.25	251.34	101.43	7.60	195.25	0.034
Left cuneus	1747.51	294.29	1741.29	361.09	6.22	-105.57	118.00	0.913
Left entorhinal	362.91	67.82	382.12	64.46	-19.21	-43.90	5.47	0.126
Left fusiform	1749.54	318.41	1856.33	338.25	-106.79	-224.58	11.01	0.075
Left inferior parietal	3226.64	620.58	3184.79	537.71	41.85	-181.40	265.10	0.712
Left inferior temporal	2214.04	383.18	2238.72	433.71	-24.68	-167.98	118.63	0.735
Left isthmus cingulate	862.45	156.94	816.56	152.66	45.89	-11.41	103.18	0.116
Left lateral occipital	3600.69	760.01	3554.29	675.63	46.40	-227.83	320.64	0.739

Left lateral orbital frontal	2625.18	480.49	2759.10	513.22	-133.93	-311.84	43.98	0.139
Left lingual	1973.46	347.55	1982.19	418.18	-8.73	-140.23	122.76	0.896
Left medial orbital frontal	1239.34	225.80	1293.82	225.24	-54.48	-137.20	28.25	0.196
Left middle temporal	2275.36	409.73	2473.50	480.52	-198.14	-352.39	-43.89	0.012
Left parahippocampal	418.50	82.81	456.62	96.63	-38.12	-69.26	-6.97	0.017
Left paracentral	1291.20	210.10	1291.42	214.23	-0.22	-77.45	77.00	0.995
Left pars opercularis	1090.84	242.85	1084.14	232.46	6.71	-81.75	95.17	0.881
Left pars orbitalis	668.16	135.31	624.26	172.75	43.90	-7.93	95.73	0.097
Left pars triangularis	790.95	170.19	755.51	194.84	35.44	-28.34	99.22	0.275
Left pericalcarine	2144.77	393.54	2032.72	480.66	112.05	-37.30	261.40	0.141
Left postcentral	4239.64	725.00	4206.11	736.84	33.53	-232.83	299.89	0.804
Left posterior cingulate	963.46	173.97	957.96	151.34	5.50	-57.12	68.11	0.863
Left precentral	3508.93	634.85	3424.10	595.09	84.83	-145.77	315.43	0.469
Left precuneus	2415.44	428.98	2260.61	445.31	154.83	-3.28	312.95	0.055
Left rostral anterior cingulate	619.48	162.85	539.08	149.58	80.40	21.40	139.40	0.008
Left rostral middle frontal	3410.43	674.02	3031.40	712.64	379.03	129.87	628.19	0.003
Left superior frontal	5928.50	967.24	5752.84	1083.19	175.67	-185.38	536.72	0.339
Left superior parietal	3539.25	676.39	3550.01	673.47	-10.77	-258.52	236.98	0.932
Left superior temporal	2822.12	536.52	3070.68	542.69	-248.56	-445.54	-51.59	0.014
Left supramarginal	2692.92	495.95	2630.90	501.61	62.01	-120.06	244.09	0.503
Left frontal pole	191.02	80.32	243.92	69.44	-52.90	-81.79	-24.01	<0.001
Left temporal pole	488.86	131.35	501.33	129.61	-12.47	-60.52	35.58	0.609
Left transverse temporal	601.47	135.89	667.98	120.69	-66.51	-115.54	-17.48	0.008

Left insula	1773.68	301.85	1890.46	304.45	-116.78	-227.55	-6.01	0.039
Right banks superior temporal sulcus	449.96	118.67	506.04	131.47	-56.07	-100.28	-11.86	0.013
Right caudal anterior cingulate	1053.51	198.76	963.02	156.09	90.49	19.72	161.26	0.012
Right caudal middle frontal	1196.18	263.29	1114.77	245.25	81.41	-14.15	176.96	0.095
Right cuneus	1778.40	305.95	1933.83	363.43	-155.43	-270.89	-39.96	0.009
Right entorhinal	379.49	64.37	395.11	70.77	-15.61	-39.56	8.34	0.200
Right fusiform	2097.39	388.14	2173.35	394.49	-75.96	-218.56	66.65	0.295
Right inferior parietal	3253.08	613.91	3270.71	517.02	-17.63	-237.78	202.53	0.875
Right inferior temporal	1955.25	359.54	2014.25	419.32	-59.00	-194.21	76.21	0.391
Right isthmus cingulate	905.64	166.52	872.02	169.34	33.62	-27.57	94.80	0.280
Right lateral occipital	3546.12	695.01	3503.75	642.23	42.37	-209.61	294.35	0.741
Right lateral orbital frontal	2551.45	457.33	2708.64	452.98	-157.19	-324.57	10.19	0.066
Right lingual	2361.67	418.62	2386.40	459.93	-24.73	-180.47	131.01	0.755
Right medial orbital frontal	1353.48	234.03	1403.06	251.91	-49.59	-136.36	37.18	0.261
Right middle temporal	2599.21	470.00	2937.73	577.32	-338.52	-517.09	-159.95	<0.001
Right parahippocampal	403.38	87.94	449.00	117.07	-45.61	-79.62	-11.61	0.009
Right paracentral	1247.52	213.96	1279.36	213.04	-31.84	-110.20	46.53	0.424
Right pars opercularis	1073.96	250.43	1093.48	214.66	-19.51	-109.50	70.47	0.670
Right pars orbitalis	537.09	115.67	516.86	141.76	20.22	-23.70	64.15	0.365
Right pars triangularis	772.86	176.71	694.14	180.90	78.72	13.73	143.71	0.018
Right pericalcarine	2287.06	431.54	2257.27	514.47	29.79	-133.19	192.77	0.719
Right postcentral	3983.62	712.43	4104.96	805.53	-121.34	-387.73	145.05	0.370
Right posterior cingulate	1064.36	186.98	1058.21	167.37	6.15	-61.37	73.67	0.858

Right precentral	3199.96	609.37	3293.07	602.66	-93.11	-316.09	129.87	0.411
Right precuneus	2532.58	438.96	2561.02	435.06	-28.44	-189.11	132.24	0.728
Right rostral anterior cingulate	631.21	152.88	570.86	130.67	60.35	5.44	115.26	0.031
Right rostral middle frontal	3205.22	647.46	2763.91	680.18	441.31	202.21	680.40	<0.001
Right superior frontal	5884.39	1051.91	5750.08	1153.91	134.32	-256.92	525.56	0.499
Right superior parietal	3366.11	650.75	3504.67	618.26	-138.56	-375.36	98.23	0.250
Right superior temporal	2480.11	457.47	2919.71	558.96	-439.60	-613.22	-265.98	<0.001
Right supramarginal	2371.20	466.91	2296.71	390.55	74.48	-92.83	241.80	0.381
Right frontal pole	223.78	93.77	274.19	70.48	-50.42	-83.67	-17.17	0.003
Right temporal pole	448.57	118.15	478.57	116.38	-30.00	-73.20	13.21	0.173
Right transverse temporal	508.85	111.86	568.16	112.70	-59.31	-100.35	-18.26	0.005
Right insula	1648.54	296.94	1806.68	279.19	-158.14	-266.04	-50.23	0.004

CI= confidence interval; SD= standard deviation. **Note:** Data for all brain regions are based on *n*=193 VP infants and *n*=34 FT infants. *p*-values are derived from independent samples *t*-tests.

Supplementary Table 1b. Raw mean volumes (mm^3) per brain region for the very preterm (VP) and full-term (FT) groups, and mean differences in volumes between groups, at the 7-year time point.

Region	VP mean	VP SD	FT mean	FT SD	Mean difference	95% CI Lower	95% CI Upper	*p*-value (VP vs FT)
Intracranial	1338099.90	118348.59	1435895.60	101217.11	-97795.70	-142183.34	-53408.07	<0.001
Total brain tissue	1164702.68	108512.76	1258380.90	87271.05	-93678.22	-134066.47	-53289.96	<0.001
Extra-axial cerebrospinal fluid	152701.36	40392.83	159330.97	47016.94	-6629.60	-24185.57	10926.37	0.457
Cerebrospinal fluid	173397.21	48030.23	177514.69	48590.82	-4117.48	-22597.81	14362.86	0.661
Total cortical grey matter	570647.01	51774.54	617797.41	48729.79	-47150.39	-67074.77	-27226.02	<0.001
Left cortical grey matter	285261.33	26174.37	308919.77	24618.13	-23658.44	-33729.91	-13586.97	<0.001
Right cortical grey matter	285385.68	25789.31	308877.64	24230.77	-23491.96	-33413.48	-13570.44	<0.001
Left lateral ventricle	7218.19	4904.27	5043.33	3001.90	2174.86	292.61	4057.11	0.024
Right lateral ventricle	6685.34	4331.17	4441.51	2129.78	2243.83	605.59	3882.07	0.008
Third ventricle	853.53	362.35	716.40	146.38	137.13	1.28	272.98	0.048
Fourth ventricle	1736.61	551.57	1612.79	448.69	123.82	-94.35	341.98	0.264
Left white matter	193165.31	22836.51	212787.11	20690.42	-19621.80	-28353.94	-10889.65	<0.001
Right white matter	193372.11	22063.18	211846.37	20322.87	-18474.26	-26933.72	-10014.80	<0.001
Total white matter	386537.42	44764.27	424633.48	40884.01	-38096.06	-55235.35	-20956.77	<0.001
Corpus callosum	3157.80	510.12	3410.00	518.16	-252.20	-461.43	-42.97	0.018
Brainstem	19939.88	1896.21	22029.72	2040.95	-2089.84	-2877.63	-1302.05	<0.001
Left cerebellum	70935.87	6350.52	75541.26	6969.99	-4605.39	-7252.39	-1958.40	0.001
Right cerebellum	70213.02	6530.39	74730.78	6818.70	-4517.76	-7211.04	-1824.48	0.001
Left thalamus	6924.73	663.35	7872.80	632.63	-948.06	-1216.98	-679.15	<0.001
Left caudate	3743.07	534.29	3952.58	489.18	-209.51	-424.60	5.58	0.056

Left putamen	4838.32	568.78	5067.98	684.77	-229.67	-472.01	12.67	0.063
Left pallidum	1845.85	220.73	2036.17	205.12	-190.32	-279.40	-101.24	<0.001
Left hippocampus	3163.20	291.03	3378.34	279.37	-215.13	-333.25	-97.02	<0.001
Left amygdala	1514.54	165.81	1579.08	134.65	-64.54	-130.11	1.03	0.054
Left accumbens	477.09	100.20	507.33	131.21	-30.25	-73.92	13.43	0.173
Right thalamus	6699.32	703.79	7583.89	569.05	-884.57	-1162.71	-606.43	<0.001
Right caudate	3789.84	542.31	4023.89	466.55	-234.06	-450.26	-17.85	0.034
Right putamen	5084.97	515.13	5389.44	533.61	-304.48	-516.59	-92.37	0.005
Right pallidum	1735.74	217.92	1921.27	223.74	-185.54	-275.11	-95.97	<0.001
Right hippocampus	3290.31	313.35	3514.12	285.54	-223.82	-349.87	-97.77	0.001
Right amygdala	1682.42	170.87	1736.62	144.89	-54.20	-122.17	13.78	0.117
Right accumbens	577.13	84.14	608.76	101.65	-31.63	-67.51	4.25	0.084
Left banks superior temporal sulcus	2938.53	630.41	3416.41	783.35	-477.89	-748.71	-207.07	0.001
Left caudal anterior cingulate	2067.34	525.41	2310.83	666.78	-243.49	-470.49	-16.48	0.036
Left caudal middle frontal	7704.85	1379.80	8528.72	1407.12	-823.87	-1390.25	-257.50	0.005
Left cuneus	3793.83	625.47	3932.59	687.30	-138.75	-399.53	122.02	0.295
Left entorhinal	2178.48	600.23	2203.24	449.90	-24.76	-259.74	210.22	0.835
Left fusiform	10554.65	1317.42	11751.93	1345.57	-1197.28	-1738.22	-656.35	<0.001
Left inferior parietal	15008.13	2225.95	17176.07	2334.96	-2167.94	-3086.84	-1249.03	<0.001
Left inferior temporal	14220.45	1969.01	14729.83	1982.34	-509.38	-1315.59	296.84	0.214
Left isthmus cingulate	3230.93	504.28	3514.00	578.96	-283.08	-495.44	-70.71	0.009
Left lateral occipital	15055.97	2193.17	15255.31	2216.71	-199.34	-1098.02	699.34	0.662
Left lateral orbital frontal	9069.18	1108.07	10202.17	1189.80	-1132.99	-1592.66	-673.32	<0.001

Left lingual	7583.07	1087.48	8568.17	1121.35	-985.11	-1432.47	-537.74	<0.001
Left medial orbital frontal	5938.43	726.33	6478.24	634.75	-539.81	-830.06	-249.56	<0.001
Left middle temporal	12931.53	1652.02	15118.59	1924.63	-2187.05	-2885.21	-1488.90	<0.001
Left parahippocampal	2348.91	484.96	2460.21	292.11	-111.30	-297.19	74.59	0.239
Left paracentral	4349.58	567.37	4535.86	609.75	-186.29	-421.70	49.12	0.120
Left pars opercularis	5666.28	875.68	6508.45	733.06	-842.16	-1189.90	-494.43	<0.001
Left pars orbitalis	2997.08	474.65	3349.76	462.42	-352.68	-545.84	-159.53	<0.001
Left pars triangularis	4448.31	630.73	5168.59	922.98	-720.28	-1004.86	-435.70	<0.001
Left pericalcarine	2416.86	456.17	2485.97	577.38	-69.11	-266.05	127.84	0.489
Left postcentral	11147.05	1573.55	12454.28	1417.26	-1307.23	-1939.01	-675.44	<0.001
Left posterior cingulate	3697.18	613.91	4135.38	544.67	-438.20	-684.10	-192.30	0.001
Left precentral	14983.98	1867.87	16097.83	1542.09	-1113.84	-1854.13	-373.55	0.003
Left precuneus	12861.73	1680.70	13623.52	1727.61	-761.79	-1452.76	-70.82	0.031
Left rostral anterior cingulate	3052.67	554.81	3420.10	671.03	-367.44	-604.10	-130.77	0.003
Left rostral middle frontal	21110.78	2662.40	22140.38	2962.87	-1029.60	-2142.77	83.56	0.070
Left superior frontal	28265.43	3130.35	29746.59	2986.88	-1481.16	-2750.27	-212.05	0.022
Left superior parietal	16611.95	2505.80	18258.66	1809.01	-1646.71	-2623.54	-669.87	0.001
Left superior temporal	15435.38	2117.31	16611.97	1980.40	-1176.59	-2032.03	-321.16	0.007
Left supramarginal	13968.08	2428.17	16152.41	2342.50	-2184.34	-3170.70	-1197.98	<0.001
Left frontal pole	1371.63	238.58	1342.66	274.71	28.98	-71.57	129.52	0.570
Left temporal pole	2577.63	503.45	2577.79	344.04	-0.17	-195.31	194.97	0.999
Left transverse temporal	1427.21	299.34	1468.83	224.33	-41.62	-158.80	75.57	0.484
Left insula	8039.14	1005.11	9016.97	1380.85	-977.82	-1422.22	-533.43	<0.001

Right banks superior temporal sulcus	2549.93	451.02	3138.38	422.90	-588.45	-770.75	-406.15	<0.001
Right caudal anterior cingulate	2342.38	623.02	2523.41	618.91	-181.03	-435.48	73.42	0.162
Right caudal middle frontal	7237.01	1348.35	8252.24	1532.04	-1015.23	-1581.68	-448.79	0.001
Right cuneus	4167.27	714.53	4340.76	736.88	-173.49	-467.44	120.46	0.245
Right entorhinal	2196.78	574.75	2040.00	377.73	156.78	-65.17	378.73	0.165
Right fusiform	10294.64	1456.53	11497.41	1639.48	-1202.77	-1813.34	-592.21	<0.001
Right inferior parietal	18120.83	2618.08	20329.31	2798.44	-2208.48	-3293.50	-1123.45	<0.001
Right inferior temporal	14248.61	2363.56	14605.93	2097.20	-357.32	-1304.06	589.41	0.457
Right isthmus cingulate	2885.07	514.84	3251.66	430.39	-366.59	-570.99	-162.19	0.001
Right lateral occipital	15619.18	2482.17	16120.90	2187.42	-501.72	-1494.91	491.47	0.320
Right lateral orbital frontal	8874.74	1106.71	9863.41	1061.30	-988.67	-1437.76	-539.59	<0.001
Right lingual	8335.58	1256.74	8914.59	1100.54	-579.01	-1081.38	-76.64	0.024
Right medial orbital frontal	6697.78	748.20	7159.97	771.82	-462.18	-770.00	-154.36	0.004
Right middle temporal	13676.92	1752.06	16578.31	2046.69	-2901.39	-3642.31	-2160.48	<0.001
Right parahippocampal	2029.22	316.31	2218.59	358.34	-189.37	-322.16	-56.58	0.005
Right paracentral	4807.22	657.32	5066.24	602.11	-259.02	-523.67	5.62	0.055
Right pars opercularis	4669.49	756.71	5370.69	803.34	-701.20	-1014.36	-388.04	<0.001
Right pars orbitalis	3501.23	519.16	3777.86	526.73	-276.63	-489.52	-63.74	0.011
Right pars triangularis	5085.95	818.98	5880.34	938.73	-794.39	-1139.16	-449.63	<0.001
Right pericalcarine	2725.88	504.26	2671.10	533.42	54.77	-153.75	263.30	0.604
Right postcentral	10707.94	1709.53	11885.17	1531.75	-1177.23	-1863.04	-491.42	0.001
Right posterior cingulate	3639.36	578.10	4209.97	618.52	-570.61	-810.24	-330.97	<0.001
Right precentral	14669.71	1886.20	15648.79	1830.40	-979.08	-1746.10	-212.07	0.013

Right precuneus	13372.69	1704.71	14041.72	1743.88	-669.03	-1369.21	31.14	0.061
Right rostral anterior cingulate	2208.52	483.91	2647.07	462.57	-438.55	-634.80	-242.30	<0.001
Right rostral middle frontal	21422.13	3076.27	22608.10	3283.31	-1185.97	-2460.49	88.55	0.068
Right superior frontal	27144.16	2932.15	28941.03	3585.76	-1796.88	-3051.21	-542.54	0.005
Right superior parietal	16024.11	2418.13	17515.72	1943.45	-1491.62	-2446.52	-536.71	0.002
Right superior temporal	14203.21	1903.34	15359.03	1620.16	-1155.83	-1913.46	-398.20	0.003
Right supramarginal	12150.74	1883.00	13886.00	2414.32	-1735.26	-2551.12	-919.40	<0.001
Right frontal pole	1552.54	296.53	1456.83	283.96	95.71	-24.58	216.01	0.118
Right temporal pole	2664.90	473.48	2664.97	397.65	-0.07	-188.17	188.04	0.999
Right transverse temporal	1080.85	185.97	1198.34	199.97	-117.49	-194.67	-40.32	0.003
Right insula	7728.48	953.78	8547.69	995.01	-819.21	-1212.51	-425.92	<0.001

CI= confidence interval; SD= standard deviation. **Note:** Intracranial, total brain tissue, and cerebrospinal fluid are based on *n*=151 VP children and *n*=32 FT children; Total cortical grey matter, left cortical grey matter, right cortical grey matter, left white matter, right white matter, and total white matter, are based on *n*=132 VP children and *n*=32 FT children; Extra-axial cerebrospinal fluid volume is based on *n*=119 VP children and *n*=27 FT children; Brainstem volume is based on *n*=119 VP children and *n*=29 FT children; Data for all remaining brain regions are based on *n*=120 VP children and *n*=29 FT children. *p*-values are derived from independent samples *t*-tests.

Supplementary Table 1c. Raw mean volumes (mm^3) per brain region for the very preterm (VP) and full-term (FT) groups, and mean differences in volumes between groups, at the 13-year time point.

Region	VP mean	VP SD	FT mean	FT SD	Mean difference	95% CI Lower	95% CI Upper	*p*-value (VP vs FT)
Intracranial	1437968.09	132220.48	1550075.41	122707.28	-112107.32	-167267.50	-56947.15	<0.001
Total brain tissue	1213489.34	118914.19	1310717.60	111633.18	-97228.26	-146914.52	-47542.00	<0.001
Extra-axial cerebrospinal fluid	203947.16	40513.16	224629.21	33847.01	-20682.05	-37367.18	-3996.92	0.015
Cerebrospinal fluid	224478.75	47493.79	239357.82	37043.96	-14879.07	-34298.56	4540.42	0.132
Total cortical grey matter	565003.01	55314.83	609961.47	54362.79	-44958.46	-68222.07	-21694.85	<0.001
Left cortical grey matter	282433.79	28087.58	304886.02	27221.97	-22452.22	-34240.88	-10663.57	<0.001
Right cortical grey matter	282569.22	27335.98	305075.45	27228.62	-22506.24	-34025.99	-10986.49	<0.001
Left lateral ventricle	8664.52	6564.18	6089.67	3452.85	2574.85	-35.99	5185.68	0.053
Right lateral ventricle	8264.32	6115.59	5483.60	2231.83	2780.71	378.39	5183.04	0.024
Third ventricle	916.33	378.15	760.20	154.10	156.13	7.16	305.10	0.040
Fourth ventricle	1932.84	593.44	1640.58	383.26	292.26	53.41	531.12	0.017
Left white matter	193828.15	23945.64	214942.98	23072.11	-21114.83	-31156.60	-11073.05	<0.001
Right white matter	193647.45	23747.19	214175.68	22153.34	-20528.23	-30442.09	-10614.36	<0.001
Total white matter	387475.60	47591.13	429118.66	45180.11	-41643.05	-61558.97	-21727.14	<0.001
Corpus callosum	3271.13	595.04	3489.57	613.12	-218.44	-470.53	33.64	0.089
Brainstem	22526.25	2475.86	24563.86	2264.01	-2037.61	-3068.48	-1006.74	<0.001
Left cerebellum	70162.43	7962.72	73509.49	5992.44	-3347.07	-6591.80	-102.34	0.043
Right cerebellum	70556.03	7994.19	74416.26	6427.81	-3860.23	-7139.02	-581.43	0.021
Left thalamus	7451.50	823.27	8370.15	794.01	-918.65	-1263.94	-573.36	<0.001
Left caudate	3669.50	519.47	3878.00	434.23	-208.50	-422.45	5.45	0.056

Left putamen	4969.14	637.59	5165.10	680.14	-195.95	-467.62	75.71	0.156
Left pallidum	2011.63	229.25	2185.24	170.19	-173.61	-266.91	-80.31	<0.001
Left hippocampus	3260.20	327.44	3489.14	270.63	-228.94	-363.63	-94.24	0.001
Left amygdala	1620.02	174.09	1711.88	146.33	-91.87	-163.61	-20.12	0.012
Left accumbens	468.14	87.27	494.27	90.13	-26.13	-63.11	10.85	0.165
Right thalamus	7075.26	773.80	7973.22	518.82	-897.96	-1210.26	-585.67	<0.001
Right caudate	3739.37	528.10	4015.47	436.69	-276.10	-493.34	-58.85	0.013
Right putamen	5168.21	575.63	5462.17	540.56	-293.96	-534.49	-53.44	0.017
Right pallidum	1854.47	228.69	2003.74	215.52	-149.27	-244.88	-53.67	0.002
Right hippocampus	3327.65	313.55	3600.70	276.95	-273.05	-403.03	-143.07	<0.001
Right amygdala	1753.58	184.83	1833.34	138.27	-79.76	-155.03	-4.48	0.038
Right accumbens	577.94	97.35	618.27	89.86	-40.33	-80.92	0.25	0.051
Left banks superior temporal sulcus	2715.58	591.35	3119.08	799.54	-403.50	-668.12	-138.88	0.003
Left caudal anterior cingulate	2218.41	615.28	2553.54	802.21	-335.13	-608.06	-62.20	0.016
Left caudal middle frontal	7885.46	1389.28	8439.27	1497.75	-553.80	-1146.82	39.21	0.067
Left cuneus	3694.41	639.76	3802.85	568.12	-108.43	-373.81	156.95	0.421
Left entorhinal	2229.09	669.77	2348.50	537.76	-119.41	-394.07	155.25	0.392
Left fusiform	10493.92	1450.34	11543.00	1515.74	-1049.08	-1664.92	-433.24	0.001
Left inferior parietal	14089.19	2261.09	15953.62	2554.59	-1864.43	-2837.73	-891.12	<0.001
Left inferior temporal	14058.43	2121.40	14494.92	2199.07	-436.49	-1336.08	463.09	0.339
Left isthmus cingulate	3160.41	589.93	3357.00	610.25	-196.59	-446.67	53.49	0.123
Left lateral occipital	14229.26	2216.70	14812.38	2236.50	-583.12	-1519.11	352.86	0.220
Left lateral orbital frontal	9185.26	1075.78	10296.77	1096.30	-1111.51	-1566.46	-656.56	<0.001

Left lingual	7515.41	1227.84	8464.73	1246.70	-949.32	-1468.27	-430.36	<0.001
Left medial orbital frontal	5824.07	762.73	6238.96	697.40	-414.89	-732.46	-97.32	0.011
Left middle temporal	12837.11	1794.24	15041.35	2176.58	-2204.23	-2987.52	-1420.94	<0.001
Left parahippocampal	2415.99	513.20	2520.42	342.49	-104.43	-311.48	102.62	0.321
Left paracentral	4211.29	636.61	4336.62	651.17	-125.33	-394.71	144.05	0.360
Left pars opercularis	5691.54	972.80	6485.92	785.87	-794.39	-1193.57	-395.20	<0.001
Left pars orbitalis	2844.54	417.61	3127.69	491.68	-283.15	-464.35	-101.95	0.002
Left pars triangularis	4361.99	690.86	5166.38	936.74	-804.40	-1113.77	-495.03	<0.001
Left pericalcarine	2665.04	499.92	2700.77	561.43	-35.73	-250.68	179.23	0.743
Left postcentral	11117.40	1841.65	12040.46	1423.47	-923.06	-1675.42	-170.71	0.017
Left posterior cingulate	3711.77	654.63	4133.19	659.47	-421.42	-697.77	-145.08	0.003
Left precentral	15534.61	1946.37	16275.69	1782.39	-741.08	-1551.64	69.48	0.073
Left precuneus	12141.44	1558.65	12664.15	1431.80	-522.71	-1172.07	126.65	0.114
Left rostral anterior cingulate	3272.23	645.96	3723.19	744.08	-450.96	-730.05	-171.88	0.002
Left rostral middle frontal	21143.79	3013.38	21808.12	2739.87	-664.32	-1918.07	589.42	0.297
Left superior frontal	28408.22	3401.88	30083.19	3558.19	-1674.97	-3119.67	-230.27	0.023
Left superior parietal	15459.70	2343.74	16806.69	2103.85	-1346.99	-2320.53	-373.46	0.007
Left superior temporal	15032.54	2184.14	15917.85	1820.83	-885.30	-1784.62	14.01	0.054
Left supramarginal	13497.19	2459.11	15142.08	2507.22	-1644.88	-2684.92	-604.84	0.002
Left frontal pole	1165.39	188.14	1161.08	188.77	4.32	-75.06	83.69	0.915
Left temporal pole	2442.79	355.71	2414.19	325.65	28.59	-119.53	176.72	0.704
Left transverse temporal	1379.35	283.40	1400.35	229.51	-21.00	-137.32	95.33	0.722
Left insula	7916.74	971.93	8684.77	1122.69	-768.03	-1188.18	-347.89	<0.001

Right banks superior temporal sulcus	2364.31	459.52	2791.96	486.03	-427.65	-623.16	-232.14	<0.001
Right caudal anterior cingulate	2511.38	683.55	2809.35	638.68	-297.97	-583.39	-12.54	0.041
Right caudal middle frontal	7467.61	1437.62	8289.62	1234.32	-822.01	-1415.94	-228.08	0.007
Right cuneus	4004.86	668.14	4130.31	782.78	-125.45	-415.07	164.17	0.394
Right entorhinal	2328.53	673.23	2150.65	558.74	177.87	-99.19	454.94	0.207
Right fusiform	10245.87	1469.39	11465.62	1897.25	-1219.74	-1870.08	-569.41	<0.001
Right inferior parietal	17479.43	2881.68	19237.46	2995.60	-1758.03	-2980.58	-535.48	0.005
Right inferior temporal	14259.25	2312.31	14673.12	2193.79	-413.87	-1381.44	553.71	0.400
Right isthmus cingulate	2754.84	508.49	3073.50	392.70	-318.66	-526.37	-110.95	0.003
Right lateral occipital	14570.08	2329.27	15085.54	2343.27	-515.46	-1498.54	467.62	0.302
Right lateral orbital frontal	8706.35	1024.52	9702.12	924.17	-995.77	-1421.59	-569.94	<0.001
Right lingual	7912.33	1363.55	8544.96	1065.04	-632.63	-1190.25	-75.02	0.026
Right medial orbital frontal	6458.90	759.61	6832.38	611.33	-373.48	-685.07	-61.90	0.019
Right middle temporal	13524.44	1762.70	16291.62	2093.91	-2767.18	-3533.38	-2000.98	<0.001
Right parahippocampal	2091.00	349.79	2279.00	395.97	-188.00	-338.63	-37.37	0.015
Right paracentral	4734.73	680.10	4744.85	729.62	-10.12	-300.17	279.94	0.945
Right pars opercularis	4727.11	772.10	5428.27	874.68	-701.16	-1033.69	-368.64	<0.001
Right pars orbitalis	3403.81	439.17	3581.04	419.38	-177.23	-361.17	6.71	0.059
Right pars triangularis	4996.43	763.29	5760.81	991.64	-764.38	-1102.68	-426.07	<0.001
Right pericalcarine	3074.74	649.16	2877.00	484.43	197.74	-66.58	462.06	0.142
Right postcentral	10554.54	1742.78	11610.00	1358.49	-1055.46	-1768.02	-342.91	0.004
Right posterior cingulate	3734.89	669.35	4225.27	638.42	-490.38	-770.68	-210.09	0.001
Right precentral	15506.14	1850.83	15978.65	1859.00	-472.51	-1253.47	308.45	0.234

Right precuneus	12538.11	1620.57	13233.50	1629.73	-695.39	-1379.32	-11.47	0.046
Right rostral anterior cingulate	2323.62	462.85	2770.69	461.59	-447.07	-642.16	-251.99	<0.001
Right rostral middle frontal	21160.56	2921.17	21861.73	3372.61	-701.17	-1963.80	561.45	0.274
Right superior frontal	27638.49	3512.95	29047.23	3706.31	-1408.74	-2902.74	85.27	0.064
Right superior parietal	15099.20	2174.57	16601.54	2049.37	-1502.34	-2411.43	-593.25	0.001
Right superior temporal	13806.24	1965.12	14738.96	1615.12	-932.73	-1740.59	-124.86	0.024
Right supramarginal	11901.72	1910.22	13549.73	2275.69	-1648.01	-2478.82	-817.20	<0.001
Right frontal pole	1299.98	200.26	1237.85	214.91	62.13	-23.28	147.55	0.153
Right temporal pole	2532.05	390.74	2499.50	408.27	32.55	-133.36	198.46	0.699
Right transverse temporal	1042.21	192.06	1127.88	166.53	-85.68	-165.12	-6.24	0.035
Right insula	7936.61	949.28	8911.65	1094.51	-975.04	-1385.24	-564.84	<0.001

CI= confidence interval; SD= standard deviation. **Note:** Data for all brain regions are based on *n*=140 VP children and *n*=26 FT children. *p*-values are derived from independent samples *t*-tests.

Supplementary Table 2a. Brain growth from term-equivalent to 7 years in very preterm (VP) and full-term (FT) children and the group difference in trajectories.

**Region**	Unadjusted						Adjusted for total brain tissue volume					
	VP β	VP *p*	FT β	FT *p*	Group interaction β	Group interaction *p*	VP β	VP *p*	FT β	FT *p*	Group interaction β	Group interaction *p*
Intracranial	1.8467	0.0000	2.0494	0.0000	-0.1986	0.0015	0.0700	0.0701	0.1006	0.0265	-0.0039	0.9238
Total brain tissue	1.9014	0.0000	2.0841	0.0000	-0.2028	0.0008						
Extra-axial cerebrospinal fluid	0.9591	0.0000	1.2327	0.0000	-0.1141	0.7093	0.1380	0.6618	0.3377	0.3306	-0.0250	0.9289
Cerebrospinal fluid	1.0301	0.0000	1.3113	0.0000	-0.0838	0.7502	0.5375	0.0701	0.7724	0.0265	-0.0296	0.9238
Left cortical grey matter	1.9639	0.0000	2.1849	0.0000	-0.2341	0.0002	0.3788	0.0000	0.4493	0.0000	-0.0600	0.2895
Right cortical grey matter	1.9669	0.0000	2.1761	0.0000	-0.2212	0.0003	0.4317	0.0000	0.4935	0.0000	-0.0503	0.3502
Left lateral ventricle	0.5427	0.0000	0.4483	0.0180	0.0418	0.9099	0.0056	0.9998	-0.1403	0.8065	0.1013	0.8615
Right lateral ventricle	0.6701	0.0000	0.4462	0.0167	0.1859	0.6398	0.2044	0.6889	-0.0607	0.9172	0.2386	0.6080
Third ventricle	1.2623	0.0000	0.8149	0.0000	0.3654	0.2402	1.0990	0.0065	0.6763	0.1492	0.3909	0.3722
Fourth ventricle	1.6621	0.0000	1.5131	0.0000	0.2020	0.3319	1.3461	0.0000	1.2120	0.0000	0.2379	0.4096
Left white matter	1.8721	0.0000	2.0638	0.0000	-0.1696	0.0593	-0.7053	0.0000	-0.7716	0.0000	0.0905	0.3041
Right white matter	1.8823	0.0000	2.0464	0.0000	-0.1405	0.1196	-0.5889	0.0000	-0.6738	0.0000	0.1109	0.2098
Corpus callosum	1.8409	0.0000	1.9797	0.0000	-0.1545	0.2592	0.2192	0.1199	0.1730	0.2999	0.0169	0.9238
Brainstem	1.7524	0.0000	1.9589	0.0000	-0.1750	0.0127	0.4086	0.0000	0.5040	0.0000	-0.0350	0.6377
Left cerebellum	1.9768	0.0000	2.0946	0.0000	-0.1603	0.0104	0.9177	0.0000	0.9624	0.0000	-0.0504	0.4788
Right cerebellum	1.9571	0.0000	2.0739	0.0000	-0.1529	0.0138	0.8934	0.0000	0.9373	0.0000	-0.0420	0.5761
Left thalamus	1.6745	0.0000	2.0135	0.0000	-0.3101	0.0042	-0.4903	0.0000	-0.3445	0.0095	-0.1037	0.4369
Left caudate	1.9104	0.0000	2.0235	0.0000	-0.0319	0.7752	0.2832	0.0229	0.2188	0.1321	0.1409	0.3041

Left putamen	1.8812	0.0000	1.9578	0.0000	-0.0282	0.7919	0.6428	0.0000	0.5932	0.0000	0.0951	0.4655
Left pallidum	1.7528	0.0000	2.0026	0.0000	-0.2627	0.0103	0.3103	0.0044	0.4065	0.0017	-0.0869	0.5206
Left hippocampus	1.8629	0.0000	1.9825	0.0000	-0.1138	0.1995	0.4222	0.0000	0.4200	0.0004	0.0380	0.7169
Left amygdala	1.7914	0.0000	1.7146	0.0000	0.0168	0.8909	0.2990	0.0169	0.0812	0.5989	0.1716	0.2812
Left accumbens	1.8386	0.0000	1.9023	0.0000	0.1011	0.6241	0.3620	0.0684	0.2800	0.2332	0.2557	0.3056
Right thalamus	1.7050	0.0000	2.0561	0.0000	-0.3141	0.0010	-0.3505	0.0022	-0.2039	0.1259	-0.1035	0.3740
Right caudate	1.9015	0.0000	2.0143	0.0000	-0.0545	0.6241	0.2791	0.0184	0.2217	0.1095	0.1182	0.3502
Right putamen	1.9031	0.0000	2.0222	0.0000	-0.0835	0.3015	0.6492	0.0000	0.6521	0.0000	0.0394	0.6670
Right pallidum	1.7704	0.0000	2.0321	0.0000	-0.2979	0.0090	0.1338	0.2874	0.2325	0.1118	-0.1152	0.4451
Right hippocampus	1.8989	0.0000	1.9864	0.0000	-0.0702	0.4152	0.6229	0.0000	0.6135	0.0000	0.0547	0.6080
Right amygdala	1.8785	0.0000	1.7846	0.0000	0.0778	0.4219	0.4203	0.0001	0.2040	0.0998	0.2123	0.0682
Right accumbens	1.8716	0.0000	1.9709	0.0000	-0.0530	0.7148	0.4204	0.0041	0.3339	0.0537	0.1150	0.4788
Left banks superior temporal sulcus	1.9037	0.0000	2.2505	0.0000	-0.4012	0.0044	0.3651	0.0212	0.5282	0.0047	-0.2440	0.2098
Left caudal anterior cingulate	1.4787	0.0000	1.8544	0.0000	-0.2981	0.1588	-1.0032	0.0001	-0.8421	0.0044	-0.0504	0.8725
Left caudal middle frontal	1.8548	0.0000	2.0699	0.0000	-0.3267	0.0071	0.6306	0.0000	0.7308	0.0000	-0.2032	0.2272
Left cuneus	1.8147	0.0000	1.9559	0.0000	-0.2134	0.2508	0.0039	0.9998	-0.0189	0.9353	-0.0277	0.9238
Left entorhinal	1.7769	0.0000	1.7770	0.0000	0.0077	0.9646	1.4105	0.0000	1.3585	0.0000	0.0562	0.8615
Left fusiform	1.9323	0.0000	2.1630	0.0000	-0.2286	0.0090	0.5035	0.0000	0.6009	0.0000	-0.0820	0.4521
Left inferior parietal	1.9418	0.0000	2.3071	0.0000	-0.3555	0.0014	0.1929	0.0975	0.3583	0.0085	-0.1766	0.2098
Left inferior temporal	1.9516	0.0000	2.0261	0.0000	-0.0792	0.4565	0.4884	0.0000	0.4192	0.0006	0.0806	0.5086
Left isthmus cingulate	1.8947	0.0000	2.1099	0.0000	-0.2291	0.0899	-0.0006	0.9998	0.0481	0.7900	-0.0507	0.7632

Left lateral occipital	1.9886	0.0000	2.0316	0.0000	-0.0199	0.8760	0.2833	0.0176	0.1919	0.1703	0.1553	0.2939
Left lateral orbital frontal	1.8718	0.0000	2.1689	0.0000	-0.3131	0.0008	0.0281	0.7748	0.1573	0.1341	-0.1098	0.3041
Left lingual	1.8707	0.0000	2.1937	0.0000	-0.2946	0.0127	0.7414	0.0000	0.9622	0.0000	-0.1804	0.2939
Left medial orbital frontal	1.9581	0.0000	2.1319	0.0000	-0.3032	0.0006	0.5653	0.0000	0.6436	0.0000	-0.1669	0.1410
Left middle temporal	1.8932	0.0000	2.2450	0.0000	-0.3556	0.0001	0.2468	0.0063	0.4518	0.0000	-0.1883	0.0678
Left parahippocampal	1.8531	0.0000	1.9104	0.0000	-0.0632	0.7148	0.6980	0.0000	0.6588	0.0006	0.0598	0.7632
Left paracentral	1.9427	0.0000	2.0604	0.0000	-0.1039	0.4209	0.7630	0.0000	0.8078	0.0000	0.0173	0.9238
Left pars opercularis	1.8618	0.0000	2.2097	0.0000	-0.2186	0.0593	0.6545	0.0000	0.8820	0.0000	-0.0886	0.5703
Left pars orbitalis	1.9365	0.0000	2.2638	0.0000	-0.2176	0.0609	0.5870	0.0000	0.8209	0.0000	-0.0772	0.6080
Left pars triangularis	1.8790	0.0000	2.2862	0.0000	-0.3764	0.0008	0.7915	0.0000	1.0867	0.0000	-0.2584	0.0701
Left pericalcarine	0.5102	0.0000	0.8596	0.0000	-0.3710	0.2534	-2.3561	0.0000	-2.2650	0.0000	-0.0927	0.8615
Left postcentral	1.8212	0.0000	2.1777	0.0000	-0.3737	0.0071	-0.4917	0.0004	-0.3354	0.0432	-0.1344	0.4276
Left posterior cingulate	1.8443	0.0000	2.1503	0.0000	-0.2948	0.0137	0.0000	0.9998	0.1357	0.3808	-0.1074	0.4674
Left precentral	1.8887	0.0000	2.0533	0.0000	-0.2379	0.0090	0.4478	0.0000	0.4954	0.0000	-0.0968	0.3744
Left precuneus	2.0053	0.0000	2.1529	0.0000	-0.1688	0.0655	0.6038	0.0000	0.6396	0.0000	-0.0457	0.6670
Left rostral anterior cingulate	1.7489	0.0000	2.0918	0.0000	-0.2978	0.0316	-0.2061	0.1503	-0.0605	0.7354	-0.0856	0.6080
Left rostral middle frontal	1.9393	0.0000	2.0901	0.0000	-0.0928	0.3319	0.4511	0.0000	0.4807	0.0000	0.0587	0.6080
Left superior frontal	1.9371	0.0000	2.0746	0.0000	-0.1343	0.1196	0.5608	0.0000	0.5891	0.0000	0.0045	0.9535
Left superior parietal	1.9883	0.0000	2.2243	0.0000	-0.3346	0.0036	0.5391	0.0000	0.6504	0.0000	-0.1911	0.2098
Left superior temporal	1.9603	0.0000	2.1087	0.0000	-0.2471	0.0090	0.3298	0.0003	0.3268	0.0028	-0.0804	0.4655
Left supramarginal	1.8913	0.0000	2.2838	0.0000	-0.4163	0.0005	0.4428	0.0006	0.6944	0.0000	-0.2622	0.0683

Left frontal pole	2.1173	0.0000	1.9815	0.0000	0.1996	0.1652	1.3932	0.0000	1.2291	0.0000	0.2911	0.1421
Left temporal pole	1.9875	0.0000	1.9781	0.0000	0.0623	0.7148	0.9971	0.0000	0.9666	0.0000	0.1613	0.4146
Left transverse temporal	1.7803	0.0000	1.8040	0.0000	-0.2310	0.1549	-0.4532	0.0280	-0.6662	0.0055	-0.0150	0.9289
Left insula	1.9247	0.0000	2.2311	0.0000	-0.1974	0.0288	0.3196	0.0004	0.4492	0.0000	-0.0124	0.9238
Right banks superior temporal sulcus	1.9099	0.0000	2.3915	0.0000	-0.5328	0.0001	0.7839	0.0000	1.1518	0.0000	-0.4024	0.0107
Right caudal anterior cingulate	1.4573	0.0000	1.7525	0.0000	-0.4518	0.0483	-0.6526	0.0158	-0.6200	0.0516	-0.2290	0.4534
Right caudal middle frontal	1.8182	0.0000	2.1493	0.0000	-0.2495	0.0609	0.6221	0.0000	0.8609	0.0000	-0.1242	0.4788
Right cuneus	1.8648	0.0000	1.8846	0.0000	-0.0711	0.7296	-0.2490	0.1964	-0.3801	0.0967	0.1384	0.5671
Right entorhinal	1.7741	0.0000	1.5893	0.0000	0.1778	0.3866	0.9896	0.0000	0.7409	0.0045	0.2597	0.3502
Right fusiform	1.9112	0.0000	2.1646	0.0000	-0.2880	0.0043	0.3968	0.0001	0.5249	0.0000	-0.1413	0.2812
Right inferior parietal	1.9463	0.0000	2.2193	0.0000	-0.2546	0.0137	0.6690	0.0000	0.7933	0.0000	-0.1197	0.3744
Right inferior temporal	1.9336	0.0000	1.9702	0.0000	-0.0394	0.7482	0.4637	0.0000	0.3408	0.0099	0.1046	0.4521
Right isthmus cingulate	1.8745	0.0000	2.2067	0.0000	-0.4245	0.0044	-0.0981	0.5541	0.0690	0.7328	-0.2300	0.2482
Right lateral occipital	1.9927	0.0000	2.0910	0.0000	-0.0647	0.6364	0.4311	0.0007	0.3963	0.0085	0.0933	0.5206
Right lateral orbital frontal	1.9226	0.0000	2.1890	0.0000	-0.2790	0.0034	0.0820	0.3734	0.1758	0.0998	-0.0528	0.6083
Right lingual	1.9367	0.0000	2.1041	0.0000	-0.2367	0.0609	0.4514	0.0022	0.5051	0.0035	-0.0831	0.6080
Right medial orbital frontal	1.9858	0.0000	2.1251	0.0000	-0.1908	0.0197	0.4812	0.0000	0.4899	0.0000	-0.0478	0.6080
Right middle temporal	1.8829	0.0000	2.3295	0.0000	-0.4078	0.0000	0.4491	0.0000	0.7413	0.0000	-0.2545	0.0074
Right parahippocampal	1.8603	0.0000	2.0122	0.0000	-0.1496	0.2348	0.7915	0.0000	0.8683	0.0000	-0.0413	0.8251
Right paracentral	1.9439	0.0000	2.0541	0.0000	-0.0323	0.7919	0.7785	0.0000	0.8005	0.0000	0.0838	0.5738

Right pars opercularis	1.8309	0.0000	2.1867	0.0000	-0.3094	0.0134	0.4667	0.0010	0.6827	0.0000	-0.1630	0.3502
Right pars orbitalis	1.9624	0.0000	2.1675	0.0000	-0.2551	0.0114	0.8068	0.0000	0.9256	0.0000	-0.1311	0.3390
Right pars triangularis	1.8894	0.0000	2.2689	0.0000	-0.3757	0.0019	0.6507	0.0000	0.9189	0.0000	-0.2410	0.1410
Right pericalcarine	0.6690	0.0000	0.6567	0.0000	-0.0233	0.9342	-2.6388	0.0000	-2.9570	0.0000	0.2970	0.4534
Right postcentral	1.8442	0.0000	2.1148	0.0000	-0.3753	0.0081	-0.3981	0.0077	-0.3086	0.0835	-0.1535	0.3744
Right posterior cingulate	1.7943	0.0000	2.1794	0.0000	-0.5421	0.0000	0.3324	0.0232	0.5918	0.0006	-0.3862	0.0100
Right precentral	1.8775	0.0000	2.0118	0.0000	-0.0965	0.3319	0.5259	0.0000	0.5710	0.0000	0.0409	0.7169
Right precuneus	2.0202	0.0000	2.1312	0.0000	-0.1017	0.3015	0.5326	0.0000	0.5121	0.0000	0.0391	0.7169
Right rostral anterior cingulate	1.6820	0.0000	2.2091	0.0000	-0.5998	0.0001	0.1636	0.3795	0.5631	0.0085	-0.4424	0.0167
Right rostral middle frontal	1.9391	0.0000	2.0816	0.0000	-0.1524	0.1203	0.4634	0.0000	0.4848	0.0000	-0.0164	0.9238
Right superior frontal	1.9149	0.0000	2.0987	0.0000	-0.1133	0.2125	0.4077	0.0000	0.4705	0.0000	0.0485	0.6333
Right superior parietal	1.9850	0.0000	2.1872	0.0000	-0.3514	0.0033	0.5996	0.0000	0.6642	0.0000	-0.2064	0.1587
Right superior temporal	1.9649	0.0000	2.0981	0.0000	-0.1778	0.0593	0.4576	0.0000	0.4527	0.0001	-0.0200	0.8725
Right supramarginal	1.8842	0.0000	2.2572	0.0000	-0.4378	0.0002	0.1585	0.1991	0.3658	0.0105	-0.2495	0.0682
Right frontal pole	2.1390	0.0000	1.9016	0.0000	0.2388	0.1203	1.6793	0.0000	1.4316	0.0000	0.3017	0.1587
Right temporal pole	1.9942	0.0000	1.9657	0.0000	0.0285	0.8613	0.7579	0.0000	0.6218	0.0001	0.1668	0.3740
Right transverse temporal	1.7397	0.0000	2.0184	0.0000	-0.2445	0.1457	-0.5915	0.0051	-0.5245	0.0329	-0.0028	0.9836
Right insula	1.8832	0.0000	2.1237	0.0000	-0.2115	0.0127	0.3866	0.0000	0.4692	0.0000	-0.0455	0.6333

Note: Red text denotes $p<0.05$ (false discovery rate corrected); Regression coefficients (β) and *p*-values are derived from linear mixed effects models.

Supplementary Table 2b. Brain growth from 7 to 13 years in very preterm (VP) and full-term (FT) children and the group difference in trajectories.

Region	Unadjusted						Adjusted for total brain tissue volume					
	VP β	VP *p*	FT β	FT *p*	Group-by-time β	Group-by-time *p*	VP β	VP *p*	FT β	FT *p*	Group-by-time β	Group-by-time *p*
Intracranial	0.1968	0.0000	0.2249	0.0000	-0.0148	0.9657	0.1022	0.0000	0.1240	0.0000	-0.0249	0.9771
Total brain tissue	0.1011	0.0000	0.1066	0.0082	0.0119	0.9657						
Extra-axial cerebrospinal fluid	0.8875	0.0000	1.1464	0.0000	-0.2009	0.9657	0.8523	0.0000	1.1039	0.0000	-0.1996	0.9771
Cerebrospinal fluid	0.8112	0.0000	0.9817	0.0000	-0.1893	0.9657	0.7848	0.0000	0.9528	0.0000	-0.1916	0.9771
Left cortical grey matter	-0.0401	0.0198	-0.0449	0.3456	0.0130	0.9657	-0.1210	0.0000	-0.1312	0.0000	0.0053	0.9771
Right cortical grey matter	-0.0387	0.0225	-0.0407	0.3919	0.0128	0.9657	-0.1178	0.0000	-0.1259	0.0000	0.0056	0.9771
Left lateral ventricle	0.2272	0.0223	0.1800	0.5339	0.1713	0.9657	0.2003	0.0406	0.1522	0.5260	0.1653	0.9771
Right lateral ventricle	0.2466	0.0114	0.1893	0.5056	0.1575	0.9657	0.2228	0.0194	0.1608	0.4975	0.1512	0.9771
Third ventricle	0.0931	0.2719	0.1143	0.6381	0.1546	0.9657	0.0814	0.3385	0.0589	0.7982	0.1455	0.9771
Fourth ventricle	0.2074	0.0002	0.0593	0.7218	0.1670	0.9657	0.1917	0.0004	-0.0045	0.9891	0.1634	0.9771
Left white matter	-0.0159	0.5257	0.0161	0.8301	-0.0143	0.9657	-0.1492	0.0000	-0.1163	0.0031	-0.0337	0.9771
Right white matter	-0.0190	0.4461	0.0151	0.8301	-0.0160	0.9657	-0.1485	0.0000	-0.1088	0.0051	-0.0399	0.9771
Corpus callosum	0.0808	0.0331	0.0647	0.5552	0.0441	0.9657	0.0043	0.9197	0.0184	0.8343	0.0399	0.9771
Brainstem	0.3022	0.0000	0.3178	0.0000	-0.0352	0.9657	0.2404	0.0000	0.2338	0.0000	-0.0397	0.9771
Left cerebellum	-0.0294	0.0978	-0.0396	0.4297	0.0446	0.9657	-0.0798	0.0000	-0.1217	0.0003	0.0409	0.9771
Right cerebellum	0.0049	0.7873	0.0159	0.7687	0.0233	0.9657	-0.0456	0.0018	-0.0676	0.0505	0.0206	0.9771
Left thalamus	0.2573	0.0000	0.2523	0.0004	0.0118	0.9657	0.1554	0.0000	0.1390	0.0194	0.0141	0.9771
Left caudate	-0.0555	0.0477	-0.0778	0.3085	-0.0171	0.9657	-0.1348	0.0000	-0.1362	0.0223	-0.0245	0.9771

Left putamen	0.0529	0.0615	0.0457	0.5552	0.0243	0.9657	-0.0016	0.9490	-0.0004	0.9954	0.0254	0.9771
Left pallidum	0.2402	0.0000	0.2200	0.0012	0.0166	0.9657	0.1729	0.0000	0.1604	0.0111	0.0146	0.9771
Left hippocampus	0.0868	0.0002	0.1081	0.0714	-0.0642	0.9657	0.0182	0.4119	0.0193	0.7317	-0.0770	0.9771
Left amygdala	0.2127	0.0000	0.2882	0.0001	-0.1541	0.9657	0.1411	0.0000	0.2205	0.0012	-0.1658	0.9771
Left accumbens	-0.0522	0.3012	-0.1049	0.4677	-0.1071	0.9657	-0.1197	0.0133	-0.1570	0.1821	-0.1246	0.9771
Right thalamus	0.1861	0.0000	0.2027	0.0010	-0.1169	0.9657	0.0908	0.0000	0.1071	0.0394	-0.1253	0.9771
Right caudate	-0.0327	0.2296	-0.0305	0.6926	0.0057	0.9813	-0.1151	0.0000	-0.0960	0.0932	-0.0121	0.9771
Right putamen	0.0385	0.0781	0.0259	0.6811	0.0280	0.9657	-0.0188	0.3385	-0.0339	0.4772	0.0262	0.9771
Right pallidum	0.1851	0.0000	0.1441	0.0714	0.0530	0.9657	0.1102	0.0001	0.0714	0.3223	0.0588	0.9771
Right hippocampus	0.0345	0.1334	0.1007	0.0716	-0.1034	0.9657	-0.0268	0.2133	0.0136	0.8043	-0.1091	0.9771
Right amygdala	0.1193	0.0000	0.1793	0.0039	-0.1479	0.9657	0.0527	0.0231	0.1040	0.0562	-0.1506	0.9771
Right accumbens	0.0148	0.6723	0.0292	0.7777	-0.0247	0.9657	-0.0590	0.0800	0.0065	0.9643	-0.0446	0.9771
Left banks superior temporal sulcus	-0.1821	0.0000	-0.2217	0.0268	0.0636	0.9657	-0.2559	0.0000	-0.2545	0.0031	0.0564	0.9771
Left caudal anterior cingulate	0.1760	0.0024	0.2727	0.0634	0.0578	0.9657	0.0644	0.2529	0.1387	0.3112	0.0590	0.9771
Left caudal middle frontal	0.0408	0.2450	-0.0179	0.8499	0.0140	0.9657	-0.0156	0.6441	-0.0738	0.3473	0.0077	0.9771
Left cuneus	-0.0943	0.0663	-0.1020	0.4880	-0.0400	0.9657	-0.1765	0.0002	-0.1968	0.0810	-0.0399	0.9771
Left entorhinal	0.0276	0.6425	0.1328	0.4160	-0.1534	0.9657	0.0066	0.9197	0.1293	0.3473	-0.1641	0.9771
Left fusiform	-0.0272	0.2749	-0.0388	0.5722	0.0459	0.9657	-0.0903	0.0000	-0.1058	0.0330	0.0479	0.9771
Left inferior parietal	-0.1591	0.0000	-0.2070	0.0056	0.0591	0.9657	-0.2372	0.0000	-0.2500	0.0001	0.0575	0.9771
Left inferior temporal	-0.0332	0.2486	-0.0289	0.7230	-0.0183	0.9657	-0.1020	0.0000	-0.0949	0.1019	-0.0237	0.9771
Left isthmus cingulate	-0.0736	0.0551	-0.1237	0.2086	-0.0004	0.9972	-0.1590	0.0000	-0.2132	0.0058	-0.0086	0.9771

Left lateral occipital	-0.1573	0.0000	-0.0951	0.2823	-0.0841	0.9657	-0.2351	0.0000	-0.1981	0.0021	-0.0956	0.9771
Left lateral orbital frontal	0.0240	0.3641	0.0345	0.6425	-0.0346	0.9657	-0.0618	0.0021	-0.0656	0.1821	-0.0403	0.9771
Left lingual	-0.0267	0.4413	-0.0351	0.7218	-0.0518	0.9657	-0.0795	0.0129	-0.0925	0.2350	-0.0588	0.9771
Left medial orbital frontal	-0.0669	0.0071	-0.0856	0.1762	0.0385	0.9657	-0.1272	0.0000	-0.1843	0.0003	0.0419	0.9771
Left middle temporal	-0.0231	0.3712	-0.0064	0.9174	-0.0091	0.9657	-0.0990	0.0000	-0.0960	0.0424	-0.0103	0.9771
Left parahippocampal	0.0493	0.2466	0.0675	0.5552	-0.0156	0.9657	-0.0034	0.9373	0.0005	0.9954	-0.0193	0.9771
Left paracentral	-0.0841	0.0157	-0.1252	0.1586	0.0461	0.9657	-0.1384	0.0000	-0.2208	0.0031	0.0437	0.9771
Left pars opercularis	0.0055	0.8704	-0.0155	0.8520	-0.0103	0.9657	-0.0515	0.0950	-0.0657	0.3767	-0.0187	0.9771
Left pars orbitalis	-0.1335	0.0000	-0.1796	0.0300	-0.0107	0.9657	-0.1946	0.0000	-0.2776	0.0001	-0.0122	0.9771
Left pars triangularis	-0.0559	0.0781	-0.0195	0.8301	-0.0147	0.9657	-0.1060	0.0003	-0.0622	0.3949	-0.0170	0.9771
Left pericalcarine	0.4998	0.0000	0.4030	0.0671	-0.0308	0.9657	0.3644	0.0000	0.2589	0.2136	-0.0458	0.9771
Left postcentral	-0.0170	0.6688	-0.1031	0.3455	0.0936	0.9657	-0.1224	0.0001	-0.2334	0.0021	0.0924	0.9771
Left posterior cingulate	0.0053	0.8741	0.0024	0.9723	0.0303	0.9657	-0.0804	0.0064	-0.0898	0.2136	0.0310	0.9771
Left precentral	0.0782	0.0024	0.0426	0.5552	0.0823	0.9657	0.0118	0.6179	-0.0423	0.4288	0.0803	0.9771
Left precuneus	-0.1455	0.0000	-0.1782	0.0039	0.0084	0.9657	-0.2095	0.0000	-0.2565	0.0000	0.0067	0.9771
Left rostral anterior cingulate	0.1399	0.0003	0.2093	0.0371	-0.0714	0.9657	0.0560	0.1086	0.1300	0.1067	-0.0699	0.9771
Left rostral middle frontal	-0.0132	0.6305	-0.0340	0.6477	-0.0126	0.9657	-0.0756	0.0007	-0.1205	0.0216	-0.0017	0.9907
Left superior frontal	0.0020	0.9267	0.0354	0.5960	-0.0013	0.9956	-0.0595	0.0031	-0.0498	0.3223	0.0024	0.9907
Left superior parietal	-0.1800	0.0000	-0.2104	0.0070	0.1119	0.9657	-0.2458	0.0000	-0.2863	0.0000	0.1162	0.9771
Left superior temporal	-0.0722	0.0075	-0.0927	0.1762	0.0531	0.9657	-0.1459	0.0000	-0.1776	0.0005	0.0674	0.9771
Left supramarginal	-0.0864	0.0099	-0.1608	0.0573	0.0557	0.9657	-0.1541	0.0000	-0.2295	0.0009	0.0555	0.9771

Left frontal pole	-0.3773	0.0000	-0.3285	0.0005	-0.1020	0.9657	-0.4111	0.0000	-0.4090	0.0000	-0.1086	0.9771
Left temporal pole	-0.1325	0.0019	-0.1588	0.1514	-0.0398	0.9657	-0.1722	0.0000	-0.2707	0.0026	-0.0352	0.9771
Left transverse temporal	-0.1036	0.0198	-0.1782	0.1086	0.2376	0.9657	-0.2026	0.0000	-0.2563	0.0072	0.2530	0.9771
Left insula	-0.0441	0.0856	-0.1145	0.0714	0.0165	0.9657	-0.1181	0.0000	-0.1746	0.0004	0.0173	0.9771
Right banks superior temporal sulcus	-0.1764	0.0000	-0.3032	0.0005	0.0981	0.9657	-0.2310	0.0000	-0.3547	0.0000	0.0912	0.9771
Right caudal anterior cingulate	0.1885	0.0032	0.3065	0.0574	-0.1833	0.9657	0.0871	0.1579	0.2747	0.0522	-0.2043	0.9771
Right caudal middle frontal	0.0676	0.0781	0.0189	0.8512	-0.0710	0.9657	0.0113	0.7682	-0.0611	0.4872	-0.0777	0.9771
Right cuneus	-0.1315	0.0105	-0.1426	0.3085	-0.0317	0.9657	-0.2274	0.0000	-0.2968	0.0059	-0.0214	0.9771
Right entorhinal	0.1137	0.0433	0.1042	0.5272	-0.0451	0.9657	0.0782	0.1572	0.0593	0.6666	-0.0487	0.9771
Right fusiform	-0.0321	0.2642	-0.0104	0.8893	0.0111	0.9657	-0.0966	0.0000	-0.0930	0.1035	0.0164	0.9771
Right inferior parietal	-0.0988	0.0009	-0.1388	0.0639	0.0633	0.9657	-0.1578	0.0000	-0.1689	0.0059	0.0585	0.9771
Right inferior temporal	-0.0131	0.6688	0.0093	0.9038	0.0171	0.9657	-0.0769	0.0043	-0.0320	0.6444	0.0226	0.9771
Right isthmus cingulate	-0.1305	0.0021	-0.1568	0.1526	0.0398	0.9657	-0.2247	0.0000	-0.2661	0.0021	0.0245	0.9771
Right lateral occipital	-0.1829	0.0000	-0.1708	0.0397	-0.0157	0.9657	-0.2534	0.0000	-0.2587	0.0001	-0.0181	0.9771
Right lateral orbital frontal	-0.0600	0.0238	-0.0471	0.5420	-0.0429	0.9657	-0.1466	0.0000	-0.1476	0.0033	-0.0549	0.9771
Right lingual	-0.1284	0.0002	-0.1031	0.2928	-0.0409	0.9657	-0.2015	0.0000	-0.2036	0.0072	-0.0490	0.9771
Right medial orbital frontal	-0.0949	0.0000	-0.1129	0.0574	0.0594	0.9657	-0.1643	0.0000	-0.1914	0.0000	0.0590	0.9771
Right middle temporal	-0.0323	0.1892	-0.0443	0.5296	-0.0320	0.9657	-0.1000	0.0000	-0.0989	0.0366	-0.0339	0.9771
Right parahippocampal	0.0620	0.0747	0.0770	0.4297	-0.0474	0.9657	0.0148	0.6698	0.0033	0.9891	-0.0477	0.9771
Right paracentral	-0.0538	0.0856	-0.1616	0.0377	0.1371	0.9657	-0.1060	0.0002	-0.2396	0.0004	0.1386	0.9771

Right pars opercularis	0.0278	0.4461	0.0312	0.7691	-0.0473	0.9657	-0.0355	0.3088	-0.0230	0.7999	-0.0531	0.9771
Right pars orbitalis	-0.0722	0.0132	-0.1263	0.0772	0.0375	0.9657	-0.1229	0.0000	-0.2041	0.0007	0.0386	0.9771
Right pars triangularis	-0.0449	0.2032	-0.0570	0.5552	-0.0358	0.9657	-0.1033	0.0008	-0.1198	0.1035	-0.0451	0.9771
Right pericalcarine	0.6107	0.0000	0.4111	0.0377	-0.0067	0.9956	0.4506	0.0000	0.2342	0.2136	-0.0086	0.9907
Right postcentral	-0.0550	0.1816	-0.0674	0.5552	0.1056	0.9657	-0.1553	0.0000	-0.1995	0.0111	0.1113	0.9771
Right posterior cingulate	0.0456	0.1953	0.0070	0.9271	-0.0063	0.9813	-0.0223	0.5210	-0.0734	0.3647	-0.0172	0.9771
Right precentral	0.1215	0.0000	0.0673	0.3778	0.0754	0.9657	0.0620	0.0073	-0.0358	0.5260	0.0816	0.9771
Right precuneus	-0.1636	0.0000	-0.1565	0.0185	-0.0166	0.9657	-0.2314	0.0000	-0.2257	0.0000	-0.0207	0.9771
Right rostral anterior cingulate	0.1170	0.0075	0.1513	0.1762	0.0231	0.9657	0.0487	0.2507	0.0636	0.5260	0.0231	0.9771
Right rostral middle frontal	-0.0372	0.1871	-0.0682	0.3756	0.0187	0.9657	-0.1033	0.0000	-0.1469	0.0086	0.0187	0.9771
Right superior frontal	0.0291	0.2473	0.0147	0.8301	0.0183	0.9657	-0.0341	0.1163	-0.0834	0.1001	0.0280	0.9771
Right superior parietal	-0.1612	0.0000	-0.1392	0.1025	0.0754	0.9657	-0.2221	0.0000	-0.1926	0.0037	0.0752	0.9771
Right superior temporal	-0.0712	0.0073	-0.0907	0.1762	0.0360	0.9657	-0.1398	0.0000	-0.1717	0.0008	0.0421	0.9771
Right supramarginal	-0.0562	0.0856	-0.0694	0.4622	0.0110	0.9657	-0.1356	0.0000	-0.1542	0.0170	0.0075	0.9771
Right frontal pole	-0.4100	0.0000	-0.3472	0.0006	0.0290	0.9657	-0.4366	0.0000	-0.4078	0.0000	0.0150	0.9771
Right temporal pole	-0.1224	0.0029	-0.1491	0.1586	0.0690	0.9657	-0.1789	0.0000	-0.2154	0.0103	0.0607	0.9771
Right transverse temporal	-0.1104	0.0165	-0.2382	0.0377	0.0644	0.9657	-0.2191	0.0000	-0.3688	0.0002	0.0631	0.9771
Right insula	0.0532	0.0286	0.1042	0.0824	-0.0105	0.9657	-0.0104	0.6306	0.0445	0.3670	0.0004	0.9953

Note: Red text denotes $p<0.05$ (false discovery rate corrected); Regression coefficients (β) and *p*-values are derived from linear mixed effects models.

Supplementary Table 3a. Brain growth from term-equivalent to 7 years in male and female children and the sex difference in trajectories.

Region	Unadjusted						Adjusted for total brain tissue volume					
	Male β	Male *p*	Female β	Female *p*	Sex-by-time β	Sex-by-time *p*	Male β	Male *p*	Female β	Female *p*	Sex-by-time β	Sex-by-time *p*
Intracranial	1.9410	0.0000	1.8118	0.0000	0.1357	0.3049	0.0647	0.1217	0.0859	0.0241	0.0220	0.9110
Total brain tissue	2.0073	0.0000	1.8471	0.0000	0.1278	0.3049						
Extra-axial cerebrospinal fluid	0.9212	0.0000	1.0912	0.0000	0.0886	0.9144	0.0556	0.8711	0.2932	0.3115	0.0454	0.9693
Cerebrospinal fluid	1.0177	0.0000	1.1373	0.0000	0.1989	0.6956	0.4973	0.1217	0.6595	0.0241	0.1690	0.9110
Left cortical grey matter	2.0659	0.0000	1.9303	0.0000	0.1143	0.3116	0.3836	0.0000	0.3973	0.0000	0.0033	0.9693
Right cortical grey matter	2.0684	0.0000	1.9298	0.0000	0.1193	0.3049	0.4387	0.0000	0.4449	0.0000	0.0126	0.9693
Left lateral ventricle	0.5455	0.0000	0.5074	0.0000	-0.0467	0.9785	-0.0224	0.9731	-0.0131	0.9768	-0.0816	0.9693
Right lateral ventricle	0.6832	0.0000	0.5797	0.0000	0.0422	0.9785	0.1919	0.7290	0.1291	0.8066	0.0196	0.9693
Third ventricle	1.2509	0.0000	1.1220	0.0000	-0.0033	0.9977	1.0898	0.0111	0.9674	0.0161	0.0711	0.9693
Fourth ventricle	1.7217	0.0000	1.5471	0.0000	0.2602	0.5069	1.4016	0.0000	1.2417	0.0000	0.3280	0.8902
Left white matter	2.0527	0.0000	1.7443	0.0000	0.3438	0.0178	-0.6846	0.0000	-0.7497	0.0000	0.1045	0.8902
Right white matter	2.0519	0.0000	1.7569	0.0000	0.3332	0.0178	-0.5742	0.0000	-0.6332	0.0000	0.1011	0.8902
Corpus callosum	1.9332	0.0000	1.7889	0.0000	0.1188	0.6956	0.2120	0.1604	0.2115	0.1288	-0.0470	0.9693
Brainstem	1.8556	0.0000	1.7116	0.0000	0.1945	0.1901	0.4439	0.0000	0.4027	0.0000	0.1387	0.8902
Left cerebellum	2.0623	0.0000	1.9248	0.0000	0.0690	0.6473	0.9536	0.0000	0.8942	0.0000	0.0500	0.9110
Right cerebellum	2.0501	0.0000	1.8968	0.0000	0.0950	0.4483	0.9367	0.0000	0.8616	0.0000	0.0784	0.9110
Left thalamus	1.7852	0.0000	1.6709	0.0000	0.1609	0.4483	-0.4942	0.0000	-0.4372	0.0001	0.0112	0.9693
Left caudate	1.9827	0.0000	1.8711	0.0000	0.2425	0.2686	0.2599	0.0509	0.2866	0.0206	0.0972	0.9110

Left putamen	1.9638	0.0000	1.8185	0.0000	0.2233	0.3049	0.6552	0.0000	0.6129	0.0000	0.1159	0.9110
Left pallidum	1.8721	0.0000	1.7096	0.0000	0.1416	0.5036	0.3527	0.0024	0.2969	0.0061	0.0710	0.9110
Left hippocampus	1.9286	0.0000	1.8329	0.0000	0.1050	0.5576	0.4115	0.0002	0.4329	0.0000	0.0365	0.9693
Left amygdala	1.8444	0.0000	1.7081	0.0000	0.0397	0.9144	0.2695	0.0438	0.2578	0.0358	-0.0631	0.9409
Left accumbens	1.8835	0.0000	1.8119	0.0000	0.3373	0.3049	0.3228	0.1303	0.3766	0.0527	0.2274	0.9110
Right thalamus	1.8218	0.0000	1.6990	0.0000	0.1825	0.3049	-0.3458	0.0046	-0.3066	0.0068	0.0308	0.9693
Right caudate	1.9755	0.0000	1.8604	0.0000	0.2090	0.3049	0.2574	0.0421	0.2832	0.0161	0.0729	0.9110
Right putamen	1.9888	0.0000	1.8515	0.0000	0.1948	0.2686	0.6676	0.0000	0.6304	0.0000	0.1058	0.9110
Right pallidum	1.8936	0.0000	1.7270	0.0000	0.1083	0.6956	0.1656	0.2109	0.1327	0.2800	0.0061	0.9693
Right hippocampus	1.9337	0.0000	1.8911	0.0000	0.0706	0.6956	0.5948	0.0000	0.6499	0.0000	0.0184	0.9693
Right amygdala	1.9098	0.0000	1.8127	0.0000	0.0713	0.7300	0.3754	0.0007	0.3962	0.0001	-0.0274	0.9693
Right accumbens	1.9034	0.0000	1.8713	0.0000	0.1068	0.6956	0.3554	0.0231	0.4612	0.0014	-0.0595	0.9693
Left banks superior temporal sulcus	2.1096	0.0000	1.8002	0.0000	0.2217	0.4234	0.4727	0.0052	0.3042	0.0519	0.0376	0.9693
Left caudal anterior cingulate	1.6117	0.0000	1.4636	0.0000	0.2732	0.5036	-0.9981	0.0002	-0.9547	0.0001	0.1359	0.9163
Left caudal middle frontal	1.8713	0.0000	1.9104	0.0000	-0.2190	0.3333	0.5776	0.0001	0.7210	0.0000	-0.3103	0.7211
Left cuneus	1.9457	0.0000	1.7219	0.0000	0.1075	0.8130	0.0392	0.8711	-0.0416	0.8585	-0.0009	0.9962
Left entorhinal	1.8783	0.0000	1.6678	0.0000	0.2230	0.6411	1.4829	0.0000	1.3154	0.0000	0.1743	0.9110
Left fusiform	2.0263	0.0000	1.9098	0.0000	0.1198	0.5036	0.5193	0.0000	0.5192	0.0000	0.0249	0.9693
Left inferior parietal	2.0483	0.0000	1.9517	0.0000	0.1124	0.6956	0.1918	0.1260	0.2495	0.0292	-0.0759	0.9110
Left inferior temporal	2.0085	0.0000	1.9156	0.0000	0.0854	0.6956	0.4602	0.0000	0.4954	0.0000	-0.0167	0.9693
Left isthmus cingulate	2.0048	0.0000	1.8495	0.0000	0.1328	0.6956	0.0031	0.9833	0.0117	0.9414	-0.0117	0.9693

Left lateral occipital	2.0628	0.0000	1.9235	0.0000	0.1764	0.4327	0.2690	0.0353	0.2680	0.0239	0.1046	0.9110
Left lateral orbital frontal	1.9731	0.0000	1.8640	0.0000	0.0833	0.6956	0.0314	0.7620	0.0678	0.4522	-0.0050	0.9693
Left lingual	1.9545	0.0000	1.8906	0.0000	0.1097	0.6956	0.7638	0.0000	0.7912	0.0000	0.0381	0.9693
Left medial orbital frontal	2.0681	0.0000	1.8989	0.0000	-0.0393	0.9015	0.6100	0.0000	0.5436	0.0000	-0.0771	0.9110
Left middle temporal	2.0283	0.0000	1.8677	0.0000	0.1545	0.3800	0.2946	0.0022	0.2640	0.0031	0.0577	0.9110
Left parahippocampal	1.9171	0.0000	1.8038	0.0000	0.1037	0.7604	0.7004	0.0001	0.6823	0.0000	0.0514	0.9693
Left paracentral	1.9993	0.0000	1.9219	0.0000	0.0995	0.7028	0.7664	0.0000	0.7742	0.0000	0.0927	0.9110
Left pars opercularis	1.9005	0.0000	1.9387	0.0000	0.1701	0.4483	0.6208	0.0000	0.7668	0.0000	0.0791	0.9110
Left pars orbitalis	2.0122	0.0000	1.9665	0.0000	0.2225	0.3049	0.5983	0.0000	0.6531	0.0000	0.1991	0.8902
Left pars triangularis	1.9931	0.0000	1.8950	0.0000	0.1477	0.5036	0.8425	0.0000	0.8355	0.0000	0.0666	0.9398
Left pericalcarine	0.5889	0.0000	0.5446	0.0000	0.0095	0.9975	-2.4273	0.0000	-2.2493	0.0000	-0.1805	0.9484
Left postcentral	1.9014	0.0000	1.8565	0.0000	0.0173	0.9785	-0.5362	0.0003	-0.3917	0.0047	-0.1089	0.9110
Left posterior cingulate	2.0024	0.0000	1.7784	0.0000	0.2419	0.3049	0.0605	0.6930	-0.0194	0.8936	0.1256	0.9110
Left precentral	1.9581	0.0000	1.8701	0.0000	-0.0302	0.9144	0.4417	0.0000	0.4702	0.0000	-0.1083	0.9110
Left precuneus	2.0839	0.0000	1.9710	0.0000	0.0789	0.6956	0.6108	0.0000	0.6083	0.0000	-0.0135	0.9693
Left rostral anterior cingulate	1.8592	0.0000	1.7471	0.0000	0.1848	0.5036	-0.2063	0.1758	-0.1572	0.2686	0.0482	0.9693
Left rostral middle frontal	2.0278	0.0000	1.8955	0.0000	0.2257	0.2686	0.4625	0.0000	0.4488	0.0000	0.1567	0.8902
Left superior frontal	1.9965	0.0000	1.9200	0.0000	0.0817	0.6956	0.5488	0.0000	0.5831	0.0000	0.0189	0.9693
Left superior parietal	2.0923	0.0000	1.9568	0.0000	-0.0236	0.9777	0.5683	0.0000	0.5449	0.0000	-0.1059	0.9110
Left superior temporal	2.0549	0.0000	1.9091	0.0000	-0.0133	0.9785	0.3385	0.0006	0.3195	0.0005	-0.1162	0.9110
Left supramarginal	2.0799	0.0000	1.8222	0.0000	0.2193	0.3116	0.5511	0.0001	0.4107	0.0013	0.1233	0.9110

Left frontal pole	2.1377	0.0000	2.0490	0.0000	0.1917	0.5036	1.3860	0.0000	1.3461	0.0000	0.2455	0.8902
Left temporal pole	1.9958	0.0000	1.9754	0.0000	0.1057	0.7604	0.9755	0.0000	1.0100	0.0000	0.1774	0.9110
Left transverse temporal	1.8163	0.0000	1.7496	0.0000	-0.2676	0.3809	-0.5401	0.0140	-0.4313	0.0343	-0.4780	0.4225
Left insula	2.0304	0.0000	1.9155	0.0000	0.2905	0.0764	0.3309	0.0006	0.3508	0.0001	0.1699	0.8902
Right banks superior temporal sulcus	2.0546	0.0000	1.9183	0.0000	0.0537	0.9141	0.8621	0.0000	0.8232	0.0000	-0.0169	0.9693
Right caudal anterior cingulate	1.5190	0.0000	1.4916	0.0000	-0.2252	0.6956	-0.7306	0.0109	-0.5580	0.0355	-0.4907	0.8902
Right caudal middle frontal	1.8680	0.0000	1.8775	0.0000	0.1220	0.6956	0.6100	0.0002	0.7150	0.0000	0.0808	0.9398
Right cuneus	1.9172	0.0000	1.8151	0.0000	0.0193	0.9794	-0.2935	0.1540	-0.2452	0.1950	-0.0366	0.9693
Right entorhinal	1.8034	0.0000	1.6795	0.0000	0.1126	0.8218	0.9785	0.0000	0.9182	0.0000	0.0783	0.9693
Right fusiform	2.0586	0.0000	1.8388	0.0000	0.1641	0.4054	0.4684	0.0000	0.3628	0.0004	0.0842	0.9110
Right inferior parietal	2.0913	0.0000	1.8833	0.0000	0.2376	0.3049	0.7336	0.0000	0.6414	0.0000	0.0996	0.9110
Right inferior temporal	2.0252	0.0000	1.8475	0.0000	0.1730	0.4106	0.4654	0.0001	0.4207	0.0001	0.0152	0.9693
Right isthmus cingulate	1.9916	0.0000	1.8618	0.0000	-0.0189	0.9785	-0.0904	0.6177	-0.0506	0.7736	-0.1415	0.9110
Right lateral occipital	2.0796	0.0000	1.9328	0.0000	0.2009	0.3800	0.4364	0.0014	0.4138	0.0011	0.1174	0.9110
Right lateral orbital frontal	2.0196	0.0000	1.9090	0.0000	0.0905	0.6956	0.0800	0.4167	0.1157	0.1950	0.0308	0.9693
Right lingual	1.9888	0.0000	1.9377	0.0000	-0.0607	0.8992	0.4295	0.0063	0.4929	0.0007	-0.1110	0.9110
Right medial orbital frontal	2.0519	0.0000	1.9621	0.0000	0.0069	0.9885	0.4655	0.0000	0.5009	0.0000	-0.0988	0.9110
Right middle temporal	2.0483	0.0000	1.8570	0.0000	0.2538	0.1155	0.5300	0.0000	0.4601	0.0000	0.1310	0.8902
Right parahippocampal	1.9030	0.0000	1.8661	0.0000	0.0405	0.9144	0.7826	0.0000	0.8267	0.0000	0.0134	0.9693
Right paracentral	2.0044	0.0000	1.9164	0.0000	0.2135	0.3049	0.7795	0.0000	0.7849	0.0000	0.1660	0.9015

Right pars opercularis	1.8536	0.0000	1.9278	0.0000	0.0004	0.9977	0.4065	0.0075	0.6036	0.0000	-0.1112	0.9110
Right pars orbitalis	2.0146	0.0000	1.9761	0.0000	-0.0419	0.9141	0.8041	0.0000	0.8495	0.0000	-0.0654	0.9152
Right pars triangularis	1.9697	0.0000	1.9324	0.0000	0.0434	0.9144	0.6603	0.0000	0.7302	0.0000	-0.0259	0.9693
Right pericalcarine	0.6642	0.0000	0.6699	0.0000	-0.0631	0.9777	-2.8109	0.0000	-2.5606	0.0000	-0.2845	0.9110
Right postcentral	1.9252	0.0000	1.8492	0.0000	-0.0927	0.7756	-0.4301	0.0070	-0.3338	0.0241	-0.1999	0.8902
Right posterior cingulate	1.9400	0.0000	1.7687	0.0000	-0.0818	0.7756	0.3987	0.0109	0.3482	0.0172	-0.1551	0.9110
Right precentral	1.9644	0.0000	1.8297	0.0000	0.1956	0.3049	0.5490	0.0000	0.5162	0.0000	0.1722	0.8902
Right precuneus	2.1213	0.0000	1.9491	0.0000	0.1873	0.3049	0.5553	0.0000	0.5014	0.0000	0.0842	0.9110
Right rostral anterior cingulate	1.7907	0.0000	1.7448	0.0000	-0.0714	0.9012	0.1941	0.3265	0.2646	0.1424	-0.1400	0.9110
Right rostral middle frontal	2.0012	0.0000	1.9209	0.0000	0.0643	0.7756	0.4437	0.0000	0.4917	0.0000	-0.0401	0.9693
Right superior frontal	1.9909	0.0000	1.8958	0.0000	0.2088	0.2733	0.4079	0.0000	0.4285	0.0000	0.1597	0.8902
Right superior parietal	2.1205	0.0000	1.9080	0.0000	-0.0282	0.9777	0.6578	0.0000	0.5588	0.0000	-0.1308	0.9110
Right superior temporal	2.0390	0.0000	1.9305	0.0000	0.0368	0.9144	0.4527	0.0000	0.4614	0.0000	-0.0491	0.9398
Right supramarginal	2.0043	0.0000	1.8821	0.0000	0.0175	0.9785	0.1835	0.1618	0.2010	0.0969	-0.0856	0.9110
Right frontal pole	2.0966	0.0000	2.1037	0.0000	-0.0049	0.9975	1.6180	0.0000	1.6621	0.0000	0.0435	0.9693
Right temporal pole	2.0036	0.0000	1.9744	0.0000	0.0290	0.9777	0.7001	0.0000	0.7744	0.0000	-0.0246	0.9693
Right transverse temporal	1.7640	0.0000	1.8087	0.0000	0.0104	0.9930	-0.6887	0.0021	-0.4649	0.0254	-0.1198	0.9110
Right insula	1.9798	0.0000	1.8612	0.0000	0.1653	0.3049	0.4010	0.0000	0.3988	0.0000	0.0623	0.9110

Note: Red text denotes p<0.05 (false discovery rate corrected); Regression coefficients (β) and p-values are derived from linear mixed effects models.

Supplementary Table 3b. Brain growth from 7 to 13 years in male and female children and the sex difference in trajectories.

**Region**	Unadjusted						Adjusted for total brain tissue volume					
	Male β	Male *p*	Female β	Female *p*	Sex-by-time β	Sex-by-time *p*	Male β	Male *p*	Female β	Female *p*	Sex-by-time β	Sex-by-time *p*
Intracranial	0.2192	0.0000	0.1818	0.0000	0.0587	0.9817	0.1023	0.0000	0.1093	0.0000	-0.0120	0.9325
Total brain tissue	0.1257	0.0000	0.0763	0.0003	0.0775	0.9817						
Extra-axial cerebrospinal fluid	0.9253	0.0000	0.9336	0.0000	0.0857	0.9817	0.8798	0.0000	0.9070	0.0000	0.0571	0.9606
Cerebrospinal fluid	0.8177	0.0000	0.8609	0.0000	-0.0734	0.9817	0.7856	0.0000	0.8399	0.0000	-0.0923	0.9325
Left cortical grey matter	-0.0282	0.2213	-0.0544	0.0163	0.0393	0.9817	-0.1203	0.0000	-0.1251	0.0000	-0.0033	0.9606
Right cortical grey matter	-0.0298	0.1983	-0.0488	0.0291	0.0364	0.9817	-0.1197	0.0000	-0.1185	0.0000	-0.0053	0.9606
Left lateral ventricle	0.2037	0.1369	0.2363	0.0720	0.1674	0.9817	0.1716	0.1855	0.2149	0.0837	0.1466	0.9325
Right lateral ventricle	0.2368	0.0752	0.2377	0.0641	0.1606	0.9817	0.2076	0.1083	0.2183	0.0751	0.1340	0.9325
Third ventricle	0.0104	0.9307	0.1893	0.0898	0.1044	0.9817	-0.0146	0.8914	0.1769	0.0904	0.0076	0.9823
Fourth ventricle	0.1485	0.0512	0.2202	0.0027	-0.0412	0.9817	0.1156	0.1227	0.2076	0.0031	-0.1452	0.9325
Left white matter	-0.0284	0.4004	0.0084	0.8342	-0.0083	0.9817	-0.1816	0.0000	-0.1036	0.0000	-0.0792	0.9325
Right white matter	-0.0264	0.4203	0.0006	0.9921	0.0022	0.9817	-0.1734	0.0000	-0.1086	0.0000	-0.0651	0.9325
Corpus callosum	0.0280	0.5981	0.1321	0.0077	-0.0589	0.9817	-0.0585	0.1855	0.0764	0.0756	-0.0475	0.9325
Brainstem	0.3189	0.0000	0.2895	0.0000	-0.0020	0.9817	0.2339	0.0000	0.2451	0.0000	-0.0860	0.9325
Left cerebellum	-0.0248	0.3071	-0.0379	0.1071	0.0687	0.9817	-0.0974	0.0000	-0.0749	0.0000	-0.0240	0.9325
Right cerebellum	0.0084	0.7403	0.0048	0.8678	0.0589	0.9817	-0.0648	0.0007	-0.0323	0.0843	-0.0348	0.9325
Left thalamus	0.2771	0.0000	0.2342	0.0000	0.0540	0.9817	0.1450	0.0000	0.1611	0.0000	-0.0199	0.9606
Left caudate	-0.0775	0.0374	-0.0395	0.2852	-0.1015	0.9817	-0.1696	0.0000	-0.0980	0.0026	-0.1135	0.9325

Left putamen	0.0602	0.1203	0.0427	0.2643	0.0451	0.9817	-0.0066	0.8523	0.0043	0.8904	0.0322	0.9325
Left pallidum	0.2000	0.0000	0.2766	0.0000	-0.0825	0.9817	0.1097	0.0020	0.2366	0.0000	-0.1235	0.9325
Left hippocampus	0.1144	0.0002	0.0644	0.0391	-0.0193	0.9817	0.0255	0.3892	0.0107	0.7028	-0.1081	0.9325
Left amygdala	0.2263	0.0000	0.2237	0.0000	-0.1240	0.9817	0.1395	0.0002	0.1693	0.0000	-0.1698	0.9325
Left accumbens	-0.0542	0.4203	-0.0680	0.3096	-0.2437	0.9817	-0.1356	0.0343	-0.1151	0.0688	-0.2828	0.9325
Right thalamus	0.2117	0.0000	0.1642	0.0000	-0.1143	0.9817	0.0914	0.0016	0.0956	0.0006	-0.1808	0.9325
Right caudate	-0.0527	0.1465	-0.0104	0.8156	-0.0295	0.9817	-0.1470	0.0000	-0.0745	0.0147	-0.0611	0.9325
Right putamen	0.0293	0.3203	0.0442	0.1231	0.0099	0.9817	-0.0449	0.0809	0.0041	0.8653	-0.0310	0.9325
Right pallidum	0.1502	0.0003	0.2086	0.0000	-0.0389	0.9817	0.0565	0.1466	0.1549	0.0000	-0.0662	0.9325
Right hippocampus	0.0852	0.0042	0.0024	0.9450	0.0229	0.9817	0.0035	0.8914	-0.0458	0.0843	-0.0621	0.9325
Right amygdala	0.1375	0.0000	0.1202	0.0003	-0.1243	0.9817	0.0521	0.0979	0.0706	0.0171	-0.1794	0.9325
Right accumbens	0.0484	0.3187	-0.0163	0.7864	0.0480	0.9817	-0.0244	0.6068	-0.0742	0.0821	0.0837	0.9325
Left banks superior temporal sulcus	-0.1850	0.0003	-0.1925	0.0001	0.0462	0.9817	-0.2639	0.0000	-0.2468	0.0000	0.0767	0.9325
Left caudal anterior cingulate	0.1823	0.0196	0.2023	0.0078	0.2291	0.9817	0.0307	0.6923	0.1254	0.0751	0.1212	0.9325
Left caudal middle frontal	0.0366	0.4203	0.0254	0.6075	-0.0610	0.9817	-0.0325	0.4622	-0.0170	0.7028	-0.0973	0.9325
Left cuneus	-0.1011	0.1447	-0.0896	0.1880	-0.0884	0.9817	-0.2099	0.0007	-0.1475	0.0154	-0.1598	0.9325
Left entorhinal	0.1250	0.1177	-0.0415	0.6162	0.0888	0.9817	0.1085	0.1466	-0.0617	0.4108	0.1031	0.9325
Left fusiform	-0.0162	0.6361	-0.0431	0.1965	0.0821	0.9817	-0.0966	0.0004	-0.0887	0.0010	0.0445	0.9325
Left inferior parietal	-0.1552	0.0001	-0.1796	0.0000	0.0423	0.9817	-0.2435	0.0000	-0.2346	0.0000	0.0635	0.9325
Left inferior temporal	0.0088	0.8327	-0.0771	0.0391	0.0634	0.9817	-0.0729	0.0236	-0.1308	0.0000	0.0310	0.9325
Left isthmus cingulate	-0.0535	0.3041	-0.1124	0.0237	-0.0226	0.9817	-0.1599	0.0002	-0.1761	0.0000	-0.0856	0.9325

Left lateral occipital	-0.1143	0.0073	-0.1823	0.0000	0.0327	0.9817	-0.2164	0.0000	-0.2429	0.0000	-0.0684	0.9325
Left lateral orbital frontal	0.0301	0.4004	0.0210	0.5715	-0.0296	0.9817	-0.0807	0.0022	-0.0427	0.0975	-0.1095	0.9325
Left lingual	0.0086	0.8528	-0.0677	0.1300	-0.0206	0.9817	-0.0575	0.1797	-0.1075	0.0085	-0.0665	0.9325
Left medial orbital frontal	-0.0703	0.0338	-0.0696	0.0309	0.0312	0.9817	-0.1585	0.0000	-0.1127	0.0000	-0.0704	0.9325
Left middle temporal	-0.0332	0.3376	-0.0065	0.8687	-0.0145	0.9817	-0.1344	0.0000	-0.0600	0.0194	-0.0861	0.9325
Left parahippocampal	0.0840	0.1401	0.0180	0.7969	0.0702	0.9817	0.0151	0.7951	-0.0219	0.7009	0.0121	0.9606
Left paracentral	-0.0911	0.0511	-0.0907	0.0452	0.0076	0.9817	-0.1720	0.0000	-0.1298	0.0014	-0.1049	0.9325
Left pars opercularis	0.0482	0.2799	-0.0477	0.2852	0.0454	0.9817	-0.0172	0.6923	-0.0932	0.0161	0.0227	0.9606
Left pars orbitalis	-0.1141	0.0088	-0.1701	0.0000	-0.0354	0.9817	-0.2017	0.0000	-0.2148	0.0000	-0.1412	0.9325
Left pars triangularis	-0.0337	0.4203	-0.0674	0.1071	0.0687	0.9817	-0.0946	0.0161	-0.1036	0.0063	0.0524	0.9325
Left pericalcarine	0.4772	0.0000	0.4912	0.0000	-0.2197	0.9817	0.3049	0.0068	0.3930	0.0003	-0.3334	0.9325
Left postcentral	0.0045	0.9307	-0.0695	0.1850	0.0860	0.9817	-0.1306	0.0020	-0.1506	0.0003	-0.0100	0.9606
Left posterior cingulate	-0.0013	0.9747	0.0114	0.8342	0.0313	0.9817	-0.1128	0.0034	-0.0488	0.2021	-0.0291	0.9606
Left precentral	0.1051	0.0019	0.0371	0.2852	0.1434	0.9817	0.0179	0.5585	-0.0129	0.6832	0.0734	0.9325
Left precuneus	-0.1377	0.0000	-0.1651	0.0000	-0.0119	0.9817	-0.2235	0.0000	-0.2103	0.0000	-0.0784	0.9325
Left rostral anterior cingulate	0.1712	0.0009	0.1299	0.0130	0.0381	0.9817	0.0666	0.1466	0.0695	0.1113	0.0036	0.9823
Left rostral middle frontal	0.0198	0.5971	-0.0557	0.1084	0.0218	0.9817	-0.0645	0.0305	-0.1026	0.0004	-0.0374	0.9325
Left superior frontal	0.0421	0.1983	-0.0298	0.3455	0.1237	0.9817	-0.0396	0.1466	-0.0776	0.0027	0.0576	0.9325
Left superior parietal	-0.1775	0.0000	-0.1931	0.0000	0.1471	0.9817	-0.2656	0.0000	-0.2380	0.0000	0.0949	0.9325
Left superior temporal	-0.0644	0.0805	-0.0875	0.0135	0.0757	0.9817	-0.1621	0.0000	-0.1391	0.0000	0.0349	0.9325
Left supramarginal	-0.0970	0.0304	-0.1005	0.0213	-0.0266	0.9817	-0.1823	0.0000	-0.1491	0.0001	-0.0653	0.9325

Left frontal pole	-0.3354	0.0000	-0.4058	0.0000	-0.0155	0.9817	-0.3904	0.0000	-0.4326	0.0000	-0.1303	0.9325
Left temporal pole	-0.1202	0.0347	-0.1547	0.0058	-0.0720	0.9817	-0.1958	0.0001	-0.1799	0.0003	-0.2326	0.9325
Left transverse temporal	-0.0962	0.1178	-0.1369	0.0176	0.3034	0.9817	-0.2202	0.0000	-0.2017	0.0001	0.3043	0.9325
Left insula	-0.0447	0.2014	-0.0675	0.0444	-0.0641	0.9817	-0.1323	0.0000	-0.1217	0.0000	-0.0742	0.9325
Right banks superior temporal sulcus	-0.1954	0.0000	-0.1993	0.0000	-0.0423	0.9817	-0.2606	0.0000	-0.2407	0.0000	-0.0725	0.9325
Right caudal anterior cingulate	0.2920	0.0004	0.1173	0.1744	0.0694	0.9817	0.1905	0.0167	0.0389	0.6602	0.1246	0.9325
Right caudal middle frontal	0.0642	0.2107	0.0547	0.2852	-0.1834	0.9817	-0.0117	0.8219	0.0117	0.7992	-0.2667	0.9325
Right cuneus	-0.0963	0.1657	-0.1732	0.0099	0.0078	0.9817	-0.2350	0.0001	-0.2424	0.0000	-0.1397	0.9325
Right entorhinal	0.1657	0.0265	0.0544	0.4838	0.0234	0.9817	0.1192	0.1003	0.0278	0.7028	-0.0182	0.9606
Right fusiform	-0.0174	0.6544	-0.0406	0.2852	0.0761	0.9817	-0.1066	0.0007	-0.0846	0.0061	0.0104	0.9606
Right inferior parietal	-0.0907	0.0239	-0.1212	0.0017	0.0680	0.9817	-0.1536	0.0000	-0.1660	0.0000	0.0892	0.9325
Right inferior temporal	0.0090	0.8349	-0.0293	0.4864	0.1021	0.9817	-0.0642	0.0788	-0.0755	0.0298	0.1206	0.9325
Right isthmus cingulate	-0.1373	0.0150	-0.1321	0.0176	0.0164	0.9817	-0.2542	0.0000	-0.2069	0.0000	-0.0748	0.9325
Right lateral occipital	-0.1504	0.0005	-0.2137	0.0000	0.0574	0.9817	-0.2437	0.0000	-0.2655	0.0000	-0.0161	0.9606
Right lateral orbital frontal	-0.0556	0.1301	-0.0603	0.0875	-0.0436	0.9817	-0.1683	0.0000	-0.1237	0.0000	-0.1352	0.9325
Right lingual	-0.1177	0.0132	-0.1313	0.0047	-0.0114	0.9817	-0.2155	0.0000	-0.1871	0.0000	-0.1113	0.9325
Right medial orbital frontal	-0.0894	0.0038	-0.1070	0.0003	0.0844	0.9817	-0.1777	0.0000	-0.1590	0.0000	0.0329	0.9325
Right middle temporal	-0.0365	0.2678	-0.0319	0.3146	-0.0756	0.9817	-0.1168	0.0000	-0.0816	0.0015	-0.0882	0.9325
Right parahippocampal	0.1166	0.0101	0.0083	0.8687	0.0561	0.9817	0.0493	0.2639	-0.0260	0.5760	-0.0206	0.9606
Right paracentral	-0.0473	0.2678	-0.0976	0.0175	0.0975	0.9817	-0.1206	0.0014	-0.1350	0.0002	0.0224	0.9606

Right pars opercularis	0.1049	0.0304	-0.0541	0.2738	0.0881	0.9817	0.0320	0.4954	-0.1037	0.0158	0.0697	0.9325
Right pars orbitalis	-0.0547	0.1657	-0.1095	0.0038	0.0281	0.9817	-0.1278	0.0001	-0.1449	0.0000	-0.0519	0.9325
Right pars triangularis	-0.0248	0.6056	-0.0706	0.1231	-0.0314	0.9817	-0.0967	0.0184	-0.1159	0.0037	-0.0806	0.9325
Right pericalcarine	0.5730	0.0000	0.5831	0.0000	-0.3428	0.9817	0.3680	0.0003	0.4668	0.0000	-0.4633	0.9325
Right postcentral	-0.0270	0.6263	-0.0893	0.0904	0.2126	0.9817	-0.1633	0.0002	-0.1615	0.0002	0.1071	0.9325
Right posterior cingulate	0.0650	0.1657	0.0117	0.8342	-0.0192	0.9817	-0.0215	0.6465	-0.0403	0.3529	-0.0918	0.9325
Right precentral	0.1119	0.0017	0.1134	0.0013	0.0327	0.9817	0.0242	0.4581	0.0698	0.0189	-0.0717	0.9325
Right precuneus	-0.1742	0.0000	-0.1498	0.0000	-0.0396	0.9817	-0.2603	0.0000	-0.1984	0.0000	-0.0863	0.9325
Right rostral anterior cingulate	0.1725	0.0024	0.0688	0.2468	0.1963	0.9817	0.0793	0.1467	0.0208	0.7028	0.1202	0.9325
Right rostral middle frontal	-0.0104	0.7926	-0.0766	0.0325	0.0462	0.9817	-0.0948	0.0026	-0.1269	0.0000	-0.0083	0.9606
Right superior frontal	0.0519	0.1301	-0.0003	0.9921	0.0584	0.9817	-0.0384	0.1792	-0.0461	0.0908	-0.0268	0.9325
Right superior parietal	-0.1577	0.0003	-0.1574	0.0003	0.1566	0.9817	-0.2329	0.0000	-0.2006	0.0000	0.1373	0.9325
Right superior temporal	-0.0742	0.0350	-0.0745	0.0309	0.0269	0.9817	-0.1646	0.0000	-0.1238	0.0000	-0.0243	0.9557
Right supramarginal	-0.0731	0.1076	-0.0425	0.3244	-0.0341	0.9817	-0.1742	0.0000	-0.1004	0.0046	-0.0918	0.9325
Right frontal pole	-0.3427	0.0000	-0.4609	0.0000	0.2662	0.9817	-0.3813	0.0000	-0.4863	0.0000	0.1760	0.9325
Right temporal pole	-0.1563	0.0038	-0.0950	0.0869	0.0068	0.9817	-0.2283	0.0000	-0.1381	0.0032	-0.0511	0.9325
Right transverse temporal	-0.0939	0.1369	-0.1718	0.0038	-0.0244	0.9817	-0.2380	0.0000	-0.2488	0.0000	-0.1296	0.9325
Right insula	0.0877	0.0062	0.0335	0.3000	0.1193	0.9817	0.0062	0.8258	-0.0099	0.7040	0.1058	0.9325

Note: Red text denotes p<0.05 (false discovery rate corrected); Regression coefficients (β) and p-values are derived from linear mixed effects models.

Supplementary Table 4a. Brain growth from term-equivalent to 7 years in very preterm children born with and without moderate to severe brain abnormality and the difference in trajectories.

**Region**	Unadjusted						Adjusted for total brain tissue volume					
	Moderate-severe β	Moderate-severe *p*	No-mild β	No-mild *p*	Abnormality-by-time β	Abnormality-by-time *p*	Moderate-severe β	Moderate-severe *p*	No-mild β	No-mild *p*	Abnormality-by-time β	Abnormality-by-time *p*
Intracranial	1.7895	0.0000	1.8612	0.0000	-0.0717	0.1446	0.0858	0.0475	0.0494	0.2653	0.0364	0.4091
Total brain tissue	1.8108	0.0000	1.9253	0.0000	-0.1145	0.0292						
Extra-axial cerebrospinal fluid	1.1043	0.0000	0.9230	0.0000	0.1813	0.3605	0.3421	0.3047	0.1091	0.7423	0.2330	0.5946
Cerebrospinal fluid	1.2185	0.0000	0.9776	0.0000	0.2409	0.1737	0.6589	0.0475	0.3792	0.2653	0.2797	0.4091
Left cortical grey matter	1.8846	0.0000	1.9855	0.0000	-0.1008	0.0440	0.3771	0.0000	0.3811	0.0000	-0.0040	0.9483
Right cortical grey matter	1.8982	0.0000	1.9855	0.0000	-0.0873	0.0641	0.4334	0.0000	0.4242	0.0000	0.0092	0.9393
Left lateral ventricle	0.2217	0.2400	0.6483	0.0000	-0.4266	0.1459	-0.8168	0.1416	-0.4530	0.4260	-0.3638	0.5946
Right lateral ventricle	0.4139	0.0263	0.7551	0.0000	-0.3412	0.2412	-0.4943	0.3682	-0.2075	0.7138	-0.2869	0.6731
Third ventricle	1.0205	0.0000	1.3221	0.0000	-0.3016	0.2412	0.8622	0.0706	1.1614	0.0162	-0.2992	0.5946
Fourth ventricle	1.6505	0.0000	1.6564	0.0000	-0.0059	0.9800	1.4153	0.0000	1.4083	0.0000	0.0071	0.9953
Left white matter	1.7467	0.0000	1.9006	0.0000	-0.1538	0.0440	-0.6929	0.0000	-0.6840	0.0000	-0.0089	0.9483
Right white matter	1.7701	0.0000	1.9081	0.0000	-0.1380	0.0578	-0.5905	0.0000	-0.5991	0.0000	0.0086	0.9483
Corpus callosum	1.6935	0.0000	1.8774	0.0000	-0.1839	0.0590	0.2286	0.1478	0.3140	0.0489	-0.0854	0.6731
Brainstem	1.6676	0.0000	1.7727	0.0000	-0.1052	0.0574	0.4616	0.0000	0.4928	0.0000	-0.0313	0.7776
Left cerebellum	1.8820	0.0000	1.9996	0.0000	-0.1175	0.0292	0.9235	0.0000	0.9779	0.0000	-0.0545	0.5946
Right cerebellum	1.8811	0.0000	1.9729	0.0000	-0.0918	0.0590	0.8945	0.0000	0.9199	0.0000	-0.0254	0.7776
Left thalamus	1.6353	0.0000	1.6807	0.0000	-0.0454	0.6126	-0.2616	0.0391	-0.3416	0.0074	0.0800	0.5951

Left caudate	1.7855	0.0000	1.9427	0.0000	-0.1572	0.0440	0.2782	0.0427	0.3429	0.0131	-0.0647	0.6731
Left putamen	1.8264	0.0000	1.8948	0.0000	-0.0684	0.3917	0.6451	0.0000	0.6335	0.0000	0.0116	0.9483
Left pallidum	1.6774	0.0000	1.7625	0.0000	-0.0851	0.2961	0.4140	0.0011	0.4151	0.0012	-0.0010	0.9953
Left hippocampus	1.8673	0.0000	1.8613	0.0000	0.0060	0.9336	0.4656	0.0000	0.3695	0.0015	0.0961	0.4091
Left amygdala	1.8186	0.0000	1.7807	0.0000	0.0380	0.6770	0.3464	0.0145	0.2142	0.1334	0.1322	0.4091
Left accumbens	1.8264	0.0000	1.8448	0.0000	-0.0184	0.9049	0.4365	0.0440	0.3574	0.0997	0.0791	0.7776
Right thalamus	1.6407	0.0000	1.7214	0.0000	-0.0807	0.2961	-0.2685	0.0283	-0.3054	0.0134	0.0369	0.7776
Right caudate	1.7645	0.0000	1.9374	0.0000	-0.1729	0.0292	0.3252	0.0136	0.4126	0.0020	-0.0874	0.5946
Right putamen	1.8387	0.0000	1.9182	0.0000	-0.0795	0.2137	0.6789	0.0000	0.6827	0.0000	-0.0038	0.9857
Right pallidum	1.7019	0.0000	1.7816	0.0000	-0.0797	0.3892	0.2089	0.1395	0.1868	0.1894	0.0221	0.9393
Right hippocampus	1.8684	0.0000	1.9090	0.0000	-0.0406	0.5749	0.6593	0.0000	0.6240	0.0000	0.0353	0.7776
Right amygdala	1.8769	0.0000	1.8772	0.0000	-0.0004	0.9948	0.4857	0.0000	0.3891	0.0012	0.0967	0.5068
Right accumbens	1.8066	0.0000	1.8917	0.0000	-0.0851	0.3892	0.4305	0.0079	0.4276	0.0088	0.0029	0.9953
Left banks superior temporal sulcus	1.7174	0.0000	1.9560	0.0000	-0.2387	0.0440	0.3095	0.0706	0.4516	0.0088	-0.1421	0.5946
Left caudal anterior cingulate	1.3951	0.0000	1.4933	0.0000	-0.0982	0.5749	-0.6810	0.0127	-0.7182	0.0091	0.0373	0.9393
Left caudal middle frontal	1.8135	0.0000	1.8687	0.0000	-0.0552	0.5913	0.7441	0.0000	0.7327	0.0000	0.0114	0.9515
Left cuneus	1.8111	0.0000	1.8149	0.0000	-0.0038	0.9810	0.2232	0.3386	0.1111	0.6421	0.1121	0.7577
Left entorhinal	1.6975	0.0000	1.7939	0.0000	-0.0963	0.5913	1.1551	0.0000	1.2183	0.0000	-0.0632	0.9393
Left fusiform	1.8252	0.0000	1.9590	0.0000	-0.1338	0.0440	0.5346	0.0000	0.5771	0.0000	-0.0425	0.7776
Left inferior parietal	1.8979	0.0000	1.9582	0.0000	-0.0603	0.5388	0.2528	0.0475	0.1994	0.1214	0.0534	0.7776
Left inferior temporal	1.8806	0.0000	1.9776	0.0000	-0.0969	0.2322	0.4843	0.0000	0.4857	0.0000	-0.0014	0.9953

Left isthmus cingulate	1.7576	0.0000	1.9319	0.0000	-0.1744	0.0799	0.0453	0.7738	0.1028	0.5252	-0.0574	0.7776
Left lateral occipital	1.8690	0.0000	2.0275	0.0000	-0.1585	0.0620	0.2324	0.0795	0.2785	0.0368	-0.0460	0.7776
Left lateral orbital frontal	1.8062	0.0000	1.8920	0.0000	-0.0858	0.2412	0.0515	0.5915	0.0183	0.8427	0.0331	0.7776
Left lingual	1.8407	0.0000	1.8854	0.0000	-0.0447	0.6544	0.8492	0.0000	0.8256	0.0000	0.0236	0.9393
Left medial orbital frontal	1.9185	0.0000	1.9663	0.0000	-0.0478	0.5572	0.3895	0.0001	0.3353	0.0006	0.0542	0.6731
Left middle temporal	1.8268	0.0000	1.9096	0.0000	-0.0828	0.2501	0.3227	0.0009	0.3029	0.0021	0.0199	0.9393
Left parahippocampal	1.8185	0.0000	1.8601	0.0000	-0.0415	0.7434	0.6778	0.0006	0.6405	0.0013	0.0374	0.9393
Left paracentral	1.8979	0.0000	1.9528	0.0000	-0.0548	0.5913	0.7798	0.0000	0.7574	0.0000	0.0224	0.9393
Left pars opercularis	1.8183	0.0000	1.8758	0.0000	-0.0576	0.5749	0.7251	0.0000	0.7123	0.0000	0.0127	0.9483
Left pars orbitalis	1.9883	0.0000	1.9213	0.0000	0.0670	0.5143	0.7053	0.0000	0.5473	0.0003	0.1580	0.4091
Left pars triangularis	1.8412	0.0000	1.8903	0.0000	-0.0490	0.5826	0.8260	0.0000	0.8064	0.0000	0.0196	0.9393
Left pericalcarine	0.8132	0.0000	0.4220	0.0000	0.3912	0.0796	-1.6920	0.0001	-2.2549	0.0000	0.5629	0.0579
Left postcentral	1.7469	0.0000	1.8409	0.0000	-0.0939	0.3978	-0.4141	0.0106	-0.4655	0.0043	0.0514	0.8109
Left posterior cingulate	1.7385	0.0000	1.8714	0.0000	-0.1329	0.1737	-0.1068	0.4679	-0.0996	0.5119	-0.0073	0.9746
Left precentral	1.7755	0.0000	1.9203	0.0000	-0.1448	0.0440	0.4408	0.0000	0.4965	0.0000	-0.0557	0.6731
Left precuneus	1.8825	0.0000	2.0403	0.0000	-0.1579	0.0420	0.5467	0.0000	0.6186	0.0000	-0.0720	0.5946
Left rostral anterior cingulate	1.7167	0.0000	1.7605	0.0000	-0.0438	0.6770	-0.1864	0.2323	-0.2768	0.0813	0.0904	0.6731
Left rostral middle frontal	1.8610	0.0000	1.9640	0.0000	-0.1030	0.1737	0.3690	0.0003	0.3716	0.0003	-0.0026	0.9953
Left superior frontal	1.9118	0.0000	1.9439	0.0000	-0.0321	0.6544	0.5433	0.0000	0.4827	0.0000	0.0605	0.6703
Left superior parietal	1.9705	0.0000	1.9890	0.0000	-0.0185	0.8542	0.3921	0.0028	0.2946	0.0252	0.0976	0.5946

Left superior temporal	1.8199	0.0000	1.9977	0.0000	-0.1778	0.0292	0.2739	0.0089	0.3445	0.0011	-0.0706	0.5951
Left supramarginal	1.7547	0.0000	1.9250	0.0000	-0.1703	0.0620	0.2402	0.0960	0.3059	0.0351	-0.0656	0.7776
Left frontal pole	2.1287	0.0000	2.1121	0.0000	0.0167	0.9003	1.3464	0.0000	1.2745	0.0000	0.0718	0.7776
Left temporal pole	1.9054	0.0000	2.0151	0.0000	-0.1097	0.3627	0.8466	0.0000	0.8711	0.0000	-0.0244	0.9412
Left transverse temporal	1.6308	0.0000	1.8129	0.0000	-0.1821	0.1459	-0.4456	0.0511	-0.4006	0.0854	-0.0450	0.9393
Left insula	1.8182	0.0000	1.9550	0.0000	-0.1368	0.0440	0.3583	0.0002	0.3968	0.0001	-0.0385	0.7776
Right banks superior temporal sulcus	1.8114	0.0000	1.9309	0.0000	-0.1196	0.2412	0.7598	0.0000	0.8086	0.0000	-0.0488	0.8473
Right caudal anterior cingulate	1.3153	0.0000	1.5017	0.0000	-0.1864	0.2961	-0.8248	0.0066	-0.7796	0.0108	-0.0452	0.9393
Right caudal middle frontal	1.7757	0.0000	1.8316	0.0000	-0.0559	0.6126	0.6197	0.0003	0.6035	0.0006	0.0162	0.9483
Right cuneus	1.8407	0.0000	1.8725	0.0000	-0.0318	0.8369	-0.0407	0.8414	-0.1500	0.4938	0.1092	0.7577
Right entorhinal	1.7343	0.0000	1.7799	0.0000	-0.0456	0.7805	0.5988	0.0208	0.5638	0.0292	0.0350	0.9412
Right fusiform	1.7919	0.0000	1.9434	0.0000	-0.1515	0.0440	0.3802	0.0007	0.4310	0.0002	-0.0508	0.7749
Right inferior parietal	1.9172	0.0000	1.9524	0.0000	-0.0351	0.6770	0.5047	0.0001	0.4435	0.0006	0.0612	0.7577
Right inferior temporal	1.8853	0.0000	1.9475	0.0000	-0.0622	0.5388	0.3890	0.0028	0.3450	0.0088	0.0441	0.7973
Right isthmus cingulate	1.8355	0.0000	1.8927	0.0000	-0.0572	0.6544	-0.0831	0.6382	-0.1585	0.3908	0.0754	0.7776
Right lateral occipital	1.9259	0.0000	2.0194	0.0000	-0.0935	0.3493	0.4348	0.0028	0.4226	0.0043	0.0122	0.9483
Right lateral orbital frontal	1.8672	0.0000	1.9402	0.0000	-0.0730	0.3468	0.1263	0.2036	0.0847	0.4084	0.0416	0.7776
Right lingual	1.7983	0.0000	1.9795	0.0000	-0.1811	0.0590	0.5314	0.0016	0.6288	0.0002	-0.0973	0.6731
Right medial orbital frontal	1.9242	0.0000	2.0060	0.0000	-0.0818	0.2322	0.4152	0.0000	0.3937	0.0000	0.0215	0.9393

Right middle temporal	1.8227	0.0000	1.8977	0.0000	-0.0749	0.2961	0.4756	0.0000	0.4581	0.0000	0.0175	0.9393
Right parahippocampal	1.8360	0.0000	1.8667	0.0000	-0.0307	0.7630	0.8890	0.0000	0.8533	0.0000	0.0357	0.9393
Right paracentral	1.9248	0.0000	1.9437	0.0000	-0.0189	0.8373	0.9197	0.0000	0.8707	0.0000	0.0490	0.7776
Right pars opercularis	1.7870	0.0000	1.8487	0.0000	-0.0617	0.5749	0.4914	0.0018	0.4669	0.0035	0.0245	0.9393
Right pars orbitalis	1.9732	0.0000	1.9636	0.0000	0.0096	0.9177	0.7597	0.0000	0.6620	0.0000	0.0977	0.5946
Right pars triangularis	1.8178	0.0000	1.9132	0.0000	-0.0953	0.3227	0.6871	0.0000	0.7021	0.0000	-0.0151	0.9483
Right pericalcarine	0.9983	0.0000	0.5653	0.0000	0.4330	0.0440	-2.0786	0.0000	-2.7009	0.0000	0.6223	0.0092
Right postcentral	1.7518	0.0000	1.8657	0.0000	-0.1139	0.3266	-0.3660	0.0309	-0.3963	0.0192	0.0303	0.9393
Right posterior cingulate	1.6850	0.0000	1.8253	0.0000	-0.1403	0.1568	0.2240	0.1681	0.2687	0.1026	-0.0447	0.8640
Right precentral	1.8176	0.0000	1.8865	0.0000	-0.0689	0.3782	0.4782	0.0000	0.4560	0.0000	0.0221	0.9393
Right precuneus	1.9183	0.0000	2.0487	0.0000	-0.1304	0.0620	0.3923	0.0000	0.4140	0.0000	-0.0217	0.9393
Right rostral anterior cingulate	1.6403	0.0000	1.6972	0.0000	-0.0569	0.6544	0.2453	0.2323	0.2080	0.3306	0.0373	0.9393
Right rostral middle frontal	1.8413	0.0000	1.9750	0.0000	-0.1337	0.0620	0.5025	0.0000	0.5483	0.0000	-0.0458	0.7776
Right superior frontal	1.8901	0.0000	1.9202	0.0000	-0.0301	0.6770	0.4744	0.0000	0.4121	0.0000	0.0623	0.6731
Right superior parietal	1.9493	0.0000	1.9886	0.0000	-0.0394	0.6770	0.4305	0.0004	0.3546	0.0038	0.0759	0.6731
Right superior temporal	1.8397	0.0000	1.9961	0.0000	-0.1564	0.0440	0.4734	0.0000	0.5357	0.0000	-0.0623	0.6731
Right supramarginal	1.7118	0.0000	1.9330	0.0000	-0.2212	0.0292	0.1852	0.1478	0.3022	0.0189	-0.1171	0.4091
Right frontal pole	2.1650	0.0000	2.1302	0.0000	0.0348	0.7805	1.5960	0.0000	1.5271	0.0000	0.0690	0.7776
Right temporal pole	1.9513	0.0000	2.0060	0.0000	-0.0547	0.6544	0.8858	0.0000	0.8632	0.0000	0.0226	0.9412

Right transverse temporal	1.7449	0.0000	1.7329	0.0000	0.0120	0.9336	-0.3070	0.1879	-0.4501	0.0580	0.1431	0.5946
Right insula	1.8174	0.0000	1.9009	0.0000	-0.0835	0.2322	0.2786	0.0020	0.2503	0.0060	0.0283	0.8360

Note: Red text denotes $p<0.05$ (false discovery rate corrected); Regression coefficients (β) and *p*-values are derived from linear mixed effects models.

Supplementary Table 4b. Brain growth from 7 to 13 years in very preterm children born with and without moderate to severe brain abnormality and the difference in trajectories.

Region	Unadjusted						Adjusted for total brain tissue volume					
	Moderate-severe β	Moderate-severe *p*	No-mild β	No-mild *p*	Abnormality-by-time β	Abnormality-by-time *p*	Moderate-severe β	Moderate-severe *p*	No-mild β	No-mild *p*	Abnormality-by-time β	Abnormality-by-time *p*
Intracranial	0.1774	0.0000	0.2070	0.0000	-0.0297	0.9998	0.0926	0.0000	0.1028	0.0000	-0.0102	0.9946
Total brain tissue	0.0898	0.0251	0.1109	0.0000	-0.0210	0.9998						
Extra-axial cerebrospinal fluid	0.8709	0.0000	0.8747	0.0000	-0.0038	0.9998	0.8403	0.0000	0.8374	0.0000	0.0029	0.9946
Cerebrospinal fluid	0.7410	0.0000	0.8234	0.0000	-0.0824	0.9998	0.7112	0.0000	0.7894	0.0000	-0.0783	0.9946
Left cortical grey matter	-0.0383	0.4429	-0.0340	0.1259	-0.0044	0.9998	-0.1121	0.0000	-0.1249	0.0000	0.0128	0.9946
Right cortical grey matter	-0.0399	0.4291	-0.0324	0.1352	-0.0075	0.9998	-0.1139	0.0000	-0.1209	0.0000	0.0069	0.9946
Left lateral ventricle	0.4973	0.0491	0.1430	0.2780	0.3544	0.9998	0.4555	0.0430	0.0837	0.5314	0.3718	0.9946
Right lateral ventricle	0.4698	0.0596	0.1790	0.1485	0.2908	0.9998	0.4324	0.0466	0.1266	0.3270	0.3058	0.9946
Third ventricle	0.3858	0.0743	0.0185	0.8728	0.3674	0.9998	0.3795	0.0466	0.0023	0.9872	0.3772	0.9946
Fourth ventricle	0.2262	0.1151	0.2116	0.0021	0.0146	0.9998	0.2156	0.0789	0.1963	0.0032	0.0194	0.9946
Left white matter	-0.0055	0.9510	-0.0055	0.8728	0.0000	0.9998	-0.1287	0.0003	-0.1537	0.0000	0.0250	0.9946
Right white matter	-0.0117	0.9054	-0.0091	0.8229	-0.0026	0.9998	-0.1337	0.0002	-0.1535	0.0000	0.0197	0.9946
Corpus callosum	0.0561	0.5715	0.0967	0.0326	-0.0406	0.9998	-0.0111	0.9017	0.0166	0.6823	-0.0276	0.9946
Brainstem	0.2313	0.0000	0.3335	0.0000	-0.1022	0.8555	0.1811	0.0000	0.2673	0.0000	-0.0862	0.5045
Left cerebellum	-0.0698	0.1076	-0.0081	0.7568	-0.0617	0.9998	-0.1135	0.0002	-0.0604	0.0003	-0.0531	0.9946
Right cerebellum	-0.0609	0.1820	0.0357	0.1047	-0.0966	0.8555	-0.1076	0.0005	-0.0177	0.3383	-0.0900	0.5045
Left thalamus	0.2189	0.0010	0.2750	0.0000	-0.0562	0.9998	0.1295	0.0168	0.1743	0.0000	-0.0449	0.9946
Left caudate	-0.0410	0.5715	-0.0551	0.1047	0.0141	0.9998	-0.1110	0.0389	-0.1397	0.0000	0.0287	0.9946

Left putamen	0.0490	0.5253	0.0605	0.0829	-0.0115	0.9998	-0.0034	0.9729	-0.0005	0.9872	-0.0030	0.9946
Left pallidum	0.2396	0.0001	0.2528	0.0000	-0.0132	0.9998	0.1803	0.0015	0.1853	0.0000	-0.0050	0.9946
Left hippocampus	0.0681	0.2690	0.0953	0.0009	-0.0273	0.9998	0.0071	0.9017	0.0170	0.5314	-0.0099	0.9946
Left amygdala	0.2255	0.0018	0.2109	0.0000	0.0147	0.9998	0.1602	0.0112	0.1283	0.0001	0.0318	0.9946
Left accumbens	-0.0697	0.5945	-0.0486	0.4361	-0.0211	0.9998	-0.1347	0.1855	-0.1210	0.0249	-0.0136	0.9946
Right thalamus	0.1731	0.0045	0.1950	0.0000	-0.0220	0.9998	0.0902	0.0439	0.0912	0.0002	-0.0010	0.9946
Right caudate	-0.0101	0.9087	-0.0351	0.2834	0.0250	0.9998	-0.0765	0.1357	-0.1213	0.0000	0.0448	0.9946
Right putamen	0.0242	0.7050	0.0490	0.0751	-0.0248	0.9998	-0.0273	0.5300	-0.0123	0.6146	-0.0150	0.9946
Right pallidum	0.1935	0.0092	0.1921	0.0000	0.0015	0.9998	0.1246	0.0466	0.1131	0.0008	0.0115	0.9946
Right hippocampus	0.0659	0.2623	0.0240	0.4191	0.0419	0.9998	0.0140	0.7958	-0.0433	0.0786	0.0573	0.9946
Right amygdala	0.1141	0.0714	0.1226	0.0000	-0.0085	0.9998	0.0499	0.3273	0.0499	0.0685	0.0000	0.9993
Right accumbens	0.0230	0.8244	0.0109	0.8250	0.0120	0.9998	-0.0441	0.5442	-0.0685	0.0720	0.0244	0.9946
Left banks superior temporal sulcus	-0.1319	0.2122	-0.1909	0.0000	0.0590	0.9998	-0.1968	0.0132	-0.2674	0.0000	0.0706	0.9946
Left caudal anterior cingulate	0.1674	0.2623	0.1891	0.0046	-0.0216	0.9998	0.0774	0.5300	0.0828	0.2088	-0.0054	0.9946
Left caudal middle frontal	0.0733	0.4291	0.0285	0.5206	0.0448	0.9998	0.0273	0.7358	-0.0294	0.4665	0.0567	0.9946
Left cuneus	-0.0845	0.5417	-0.0967	0.1259	0.0123	0.9998	-0.1570	0.1357	-0.1769	0.0015	0.0199	0.9946
Left entorhinal	0.0613	0.7205	0.0314	0.7154	0.0299	0.9998	0.0357	0.8089	-0.0031	0.9812	0.0389	0.9946
Left fusiform	-0.0800	0.1922	-0.0024	0.9331	-0.0775	0.9998	-0.1394	0.0015	-0.0677	0.0044	-0.0717	0.9946
Left inferior parietal	-0.1940	0.0060	-0.1499	0.0000	-0.0441	0.9998	-0.2707	0.0000	-0.2335	0.0000	-0.0372	0.9946
Left inferior temporal	-0.0520	0.5085	-0.0280	0.4426	-0.0240	0.9998	-0.1160	0.0253	-0.1035	0.0002	-0.0124	0.9946
Left isthmus cingulate	-0.0623	0.5417	-0.0690	0.1329	0.0067	0.9998	-0.1401	0.0474	-0.1586	0.0000	0.0185	0.9946

Left lateral occipital	-0.1480	0.0596	-0.1595	0.0000	0.0115	0.9998	-0.2210	0.0001	-0.2467	0.0000	0.0257	0.9946
Left lateral orbital frontal	0.0212	0.7909	0.0267	0.4346	-0.0054	0.9998	-0.0593	0.1709	-0.0673	0.0033	0.0080	0.9946
Left lingual	-0.0580	0.5253	-0.0212	0.6510	-0.0368	0.9998	-0.1036	0.1357	-0.0743	0.0507	-0.0294	0.9946
Left medial orbital frontal	-0.0761	0.2623	-0.0560	0.0804	-0.0201	0.9998	-0.1434	0.0015	-0.1333	0.0000	-0.0101	0.9946
Left middle temporal	-0.0552	0.4291	-0.0081	0.8250	-0.0471	0.9998	-0.1239	0.0037	-0.0871	0.0001	-0.0368	0.9946
Left parahippocampal	0.0494	0.7012	0.0555	0.3110	-0.0062	0.9998	-0.0031	0.9857	-0.0054	0.9328	0.0023	0.9946
Left paracentral	-0.0977	0.2623	-0.0768	0.0708	-0.0209	0.9998	-0.1485	0.0298	-0.1352	0.0002	-0.0133	0.9946
Left pars opercularis	-0.0107	0.9190	0.0093	0.8361	-0.0200	0.9998	-0.0578	0.4200	-0.0502	0.1988	-0.0076	0.9946
Left pars orbitalis	-0.1885	0.0130	-0.1165	0.0027	-0.0720	0.9998	-0.2474	0.0001	-0.1822	0.0000	-0.0651	0.9946
Left pars triangularis	-0.0556	0.4836	-0.0550	0.1205	-0.0006	0.9998	-0.1017	0.0779	-0.1096	0.0004	0.0079	0.9946
Left pericalcarine	0.5645	0.0028	0.4662	0.0000	0.0983	0.9998	0.4509	0.0112	0.3292	0.0005	0.1216	0.9946
Left postcentral	-0.0409	0.7205	-0.0058	0.9067	-0.0351	0.9998	-0.1343	0.0596	-0.1197	0.0017	-0.0146	0.9946
Left posterior cingulate	0.0048	0.9708	0.0130	0.8164	-0.0082	0.9998	-0.0783	0.2229	-0.0841	0.0140	0.0058	0.9946
Left precentral	0.0740	0.2623	0.0840	0.0058	-0.0100	0.9998	0.0136	0.7972	0.0120	0.6430	0.0016	0.9946
Left precuneus	-0.1566	0.0074	-0.1375	0.0000	-0.0191	0.9998	-0.2148	0.0000	-0.2080	0.0000	-0.0069	0.9946
Left rostral anterior cingulate	0.1681	0.0707	0.1311	0.0036	0.0370	0.9998	0.0854	0.2392	0.0369	0.3718	0.0485	0.9946
Left rostral middle frontal	0.0194	0.8204	-0.0189	0.6138	0.0383	0.9998	-0.0440	0.3884	-0.0929	0.0003	0.0489	0.9946
Left superior frontal	-0.0096	0.9087	0.0082	0.8229	-0.0177	0.9998	-0.0710	0.1035	-0.0622	0.0076	-0.0088	0.9946
Left superior parietal	-0.2120	0.0060	-0.1652	0.0000	-0.0468	0.9998	-0.2865	0.0000	-0.2464	0.0000	-0.0401	0.9946
Left superior temporal	-0.0530	0.4836	-0.0690	0.0417	0.0160	0.9998	-0.1224	0.0103	-0.1484	0.0000	0.0261	0.9946
Left supramarginal	-0.0603	0.5253	-0.0830	0.0531	0.0228	0.9998	-0.1291	0.0466	-0.1630	0.0000	0.0339	0.9946

Left frontal pole	-0.3469	0.0001	-0.3821	0.0000	0.0352	0.9998	-0.3833	0.0000	-0.4243	0.0000	0.0410	0.9946
Left temporal pole	-0.0317	0.8187	-0.1663	0.0009	0.1347	0.9998	-0.0818	0.3602	-0.2142	0.0000	0.1324	0.9946
Left transverse temporal	-0.0750	0.5269	-0.1013	0.0693	0.0263	0.9998	-0.1647	0.0604	-0.2053	0.0000	0.0406	0.9946
Left insula	-0.0198	0.7909	-0.0483	0.1047	0.0285	0.9998	-0.0862	0.0466	-0.1253	0.0000	0.0391	0.9946
Right banks superior temporal sulcus	-0.1972	0.0212	-0.1598	0.0001	-0.0374	0.9998	-0.2457	0.0007	-0.2191	0.0000	-0.0266	0.9946
Right caudal anterior cingulate	0.2361	0.1249	0.1730	0.0243	0.0631	0.9998	0.1399	0.2807	0.0545	0.4665	0.0854	0.9946
Right caudal middle frontal	0.0485	0.6387	0.0722	0.1047	-0.0237	0.9998	0.0004	0.9951	0.0087	0.8555	-0.0083	0.9946
Right cuneus	-0.1448	0.2623	-0.1277	0.0388	-0.0170	0.9998	-0.2309	0.0207	-0.2236	0.0000	-0.0073	0.9946
Right entorhinal	0.1457	0.3263	0.1135	0.1047	0.0322	0.9998	0.0935	0.4516	0.0549	0.4330	0.0387	0.9946
Right fusiform	-0.0081	0.9228	-0.0325	0.3575	0.0245	0.9998	-0.0724	0.1357	-0.1032	0.0001	0.0308	0.9946
Right inferior parietal	-0.1262	0.0922	-0.0852	0.0233	-0.0410	0.9998	-0.1908	0.0005	-0.1606	0.0000	-0.0302	0.9946
Right inferior temporal	-0.0368	0.7012	0.0007	0.9826	-0.0376	0.9998	-0.1049	0.0779	-0.0747	0.0200	-0.0302	0.9946
Right isthmus cingulate	-0.1596	0.1407	-0.1262	0.0178	-0.0334	0.9998	-0.2497	0.0020	-0.2322	0.0000	-0.0175	0.9946
Right lateral occipital	-0.1850	0.0242	-0.1849	0.0000	-0.0002	0.9998	-0.2530	0.0001	-0.2612	0.0000	0.0082	0.9946
Right lateral orbital frontal	-0.0763	0.2623	-0.0542	0.1047	-0.0221	0.9998	-0.1556	0.0005	-0.1494	0.0000	-0.0062	0.9946
Right lingual	-0.0927	0.3110	-0.1412	0.0007	0.0485	0.9998	-0.1506	0.0393	-0.2143	0.0000	0.0638	0.9946
Right medial orbital frontal	-0.0690	0.2623	-0.1023	0.0002	0.0333	0.9998	-0.1406	0.0005	-0.1816	0.0000	0.0410	0.9946
Right middle temporal	-0.0471	0.4915	-0.0231	0.4721	-0.0239	0.9998	-0.1088	0.0129	-0.0947	0.0000	-0.0142	0.9946
Right parahippocampal	0.0446	0.6492	0.0693	0.1047	-0.0247	0.9998	0.0019	0.9857	0.0220	0.6049	-0.0201	0.9946
Right paracentral	-0.1325	0.0714	-0.0230	0.5744	-0.1095	0.9998	-0.1772	0.0037	-0.0755	0.0241	-0.1018	0.9946

Right pars opercularis	0.0353	0.7205	0.0202	0.6822	0.0151	0.9998	-0.0227	0.7958	-0.0483	0.2313	0.0255	0.9946
Right pars orbitalis	-0.0768	0.3110	-0.0738	0.0417	-0.0030	0.9998	-0.1326	0.0168	-0.1344	0.0000	0.0018	0.9946
Right pars triangularis	-0.0010	0.9980	-0.0593	0.1467	0.0583	0.9998	-0.0543	0.4374	-0.1207	0.0006	0.0664	0.9946
Right pericalcarine	0.4908	0.0048	0.6447	0.0000	-0.1540	0.9998	0.3558	0.0277	0.4742	0.0000	-0.1184	0.9946
Right postcentral	-0.0786	0.4836	-0.0407	0.4368	-0.0379	0.9998	-0.1726	0.0207	-0.1491	0.0002	-0.0235	0.9946
Right posterior cingulate	0.0233	0.8244	0.0607	0.1505	-0.0374	0.9998	-0.0397	0.5995	-0.0194	0.6362	-0.0203	0.9946
Right precentral	0.1092	0.1076	0.1354	0.0000	-0.0261	0.9998	0.0526	0.3157	0.0671	0.0140	-0.0145	0.9946
Right precuneus	-0.1427	0.0240	-0.1661	0.0000	0.0234	0.9998	-0.2130	0.0000	-0.2471	0.0000	0.0341	0.9946
Right rostral anterior cingulate	0.1671	0.1202	0.0994	0.0693	0.0676	0.9998	0.1065	0.2392	0.0273	0.6049	0.0792	0.9946
Right rostral middle frontal	-0.0009	0.9980	-0.0492	0.1352	0.0483	0.9998	-0.0599	0.2516	-0.1192	0.0000	0.0593	0.9946
Right superior frontal	0.0000	1.0000	0.0428	0.1505	-0.0428	0.9998	-0.0583	0.2209	-0.0260	0.3309	-0.0322	0.9946
Right superior parietal	-0.1792	0.0301	-0.1474	0.0003	-0.0318	0.9998	-0.2509	0.0000	-0.2227	0.0000	-0.0282	0.9946
Right superior temporal	-0.0609	0.3949	-0.0661	0.0423	0.0052	0.9998	-0.1226	0.0112	-0.1367	0.0000	0.0141	0.9946
Right supramarginal	-0.0187	0.8361	-0.0616	0.1047	0.0428	0.9998	-0.0880	0.1253	-0.1429	0.0000	0.0550	0.9946
Right frontal pole	-0.3594	0.0001	-0.4241	0.0000	0.0647	0.9998	-0.3859	0.0000	-0.4606	0.0000	0.0747	0.9946
Right temporal pole	-0.1444	0.1602	-0.1152	0.0202	-0.0292	0.9998	-0.1945	0.0172	-0.1713	0.0001	-0.0233	0.9946
Right transverse temporal	-0.1272	0.2690	-0.1038	0.0705	-0.0233	0.9998	-0.2179	0.0178	-0.2115	0.0000	-0.0064	0.9946
Right insula	0.0755	0.2345	0.0501	0.0996	0.0255	0.9998	0.0063	0.9017	-0.0241	0.3217	0.0304	0.9946

Note: Red text denotes $p<0.05$ (false discovery rate corrected); Regression coefficients (β) and *p*-values are derived from linear mixed effects models.

Supplementary Table 5a. Brain growth from term-equivalent to 7 years in very preterm children born small (SGA) or appropriate for gestational age (AGA) and the difference in trajectories.

**Region**	Unadjusted						Adjusted for total brain tissue volume					
	SGA β	SGA *p*	AGA β	AGA *p*	Growth restriction-by-time β	Growth restriction-by-time *p*	SGA β	SGA *p*	AGA β	AGA *p*	Growth restriction-by-time β	Growth restriction-by-time *p*
Intracranial	1.7618	0.0000	1.8541	0.0000	-0.0923	0.5964	0.0847	0.0655	0.1219	0.0022	-0.0371	0.4299
Total brain tissue	1.8443	0.0000	1.9061	0.0000	-0.0618	0.7351						
Extra-axial cerebrospinal fluid	0.7416	0.0003	0.9823	0.0000	-0.2407	0.7351	0.3414	0.3562	0.5557	0.0740	-0.2143	0.6410
Cerebrospinal fluid	0.7661	0.0000	1.0550	0.0000	-0.2889	0.5964	0.6509	0.0655	0.9362	0.0022	-0.2852	0.4299
Left cortical grey matter	1.9220	0.0000	1.9664	0.0000	-0.0444	0.8090	0.3946	0.0000	0.3812	0.0000	0.0134	0.8483
Right cortical grey matter	1.9264	0.0000	1.9693	0.0000	-0.0428	0.8090	0.4448	0.0000	0.4264	0.0000	0.0183	0.8054
Left lateral ventricle	0.2564	0.4148	0.5651	0.0000	-0.3087	0.8090	-0.1686	0.7960	0.1153	0.8362	-0.2839	0.7152
Right lateral ventricle	0.5043	0.1052	0.6826	0.0000	-0.1783	0.8609	0.1120	0.8421	0.2677	0.6302	-0.1557	0.8410
Third ventricle	1.0304	0.0001	1.2751	0.0000	-0.2447	0.8090	1.0738	0.0342	1.3274	0.0030	-0.2536	0.6996
Fourth ventricle	1.7166	0.0000	1.6521	0.0000	0.0645	0.9266	1.4442	0.0000	1.3641	0.0000	0.0801	0.8483
Left white matter	1.8740	0.0000	1.8663	0.0000	0.0077	0.9346	-0.6429	0.0000	-0.7254	0.0000	0.0825	0.4034
Right white matter	1.8876	0.0000	1.8769	0.0000	0.0108	0.9346	-0.5257	0.0000	-0.6229	0.0000	0.0972	0.2981
Corpus callosum	1.6162	0.0000	1.8564	0.0000	-0.2402	0.4041	0.1155	0.5189	0.2539	0.0842	-0.1384	0.5014
Brainstem	1.7208	0.0000	1.7548	0.0000	-0.0340	0.8609	0.4513	0.0000	0.4014	0.0000	0.0499	0.6359
Left cerebellum	1.9432	0.0000	1.9777	0.0000	-0.0345	0.8609	0.9041	0.0000	0.8716	0.0000	0.0324	0.7738
Right cerebellum	1.9271	0.0000	1.9573	0.0000	-0.0303	0.8609	0.8670	0.0000	0.8301	0.0000	0.0368	0.7444
Left thalamus	1.6924	0.0000	1.6723	0.0000	0.0201	0.9346	-0.3003	0.0295	-0.4476	0.0002	0.1473	0.3095

Left caudate	1.6284	0.0000	1.9341	0.0000	-0.3057	0.0177	0.1755	0.2065	0.3819	0.0018	-0.2065	0.1012
Left putamen	1.7518	0.0000	1.8920	0.0000	-0.1402	0.5964	0.5976	0.0000	0.6517	0.0000	-0.0541	0.7738
Left pallidum	1.6214	0.0000	1.7593	0.0000	-0.1379	0.5964	0.3529	0.0106	0.3754	0.0014	-0.0225	0.8902
Left hippocampus	1.9512	0.0000	1.8557	0.0000	0.0954	0.7351	0.5301	0.0000	0.3354	0.0018	0.1947	0.0856
Left amygdala	1.8055	0.0000	1.7880	0.0000	0.0175	0.9346	0.3496	0.0246	0.2359	0.0766	0.1137	0.5403
Left accumbens	1.7193	0.0000	1.8516	0.0000	-0.1322	0.8090	0.3969	0.0912	0.4396	0.0257	-0.0427	0.8902
Right thalamus	1.7551	0.0000	1.7013	0.0000	0.0538	0.8594	-0.2556	0.0481	-0.4448	0.0002	0.1892	0.0877
Right caudate	1.7047	0.0000	1.9171	0.0000	-0.2123	0.1438	0.2591	0.0559	0.3735	0.0019	-0.1145	0.4299
Right putamen	1.8331	0.0000	1.9081	0.0000	-0.0750	0.7477	0.6552	0.0000	0.6385	0.0000	0.0166	0.8902
Right pallidum	1.6239	0.0000	1.7796	0.0000	-0.1557	0.5964	0.1337	0.3915	0.1705	0.1955	-0.0368	0.8576
Right hippocampus	2.0042	0.0000	1.8913	0.0000	0.1129	0.5964	0.7694	0.0000	0.5653	0.0000	0.2041	0.0856
Right amygdala	1.7935	0.0000	1.8856	0.0000	-0.0921	0.7351	0.4337	0.0007	0.4284	0.0001	0.0053	0.9739
Right accumbens	1.7937	0.0000	1.8799	0.0000	-0.0862	0.8090	0.4253	0.0143	0.4193	0.0048	0.0060	0.9739
Left banks superior temporal sulcus	1.9018	0.0000	1.8993	0.0000	0.0025	0.9846	0.3947	0.0355	0.2966	0.0635	0.0982	0.7179
Left caudal anterior cingulate	1.4603	0.0000	1.4783	0.0000	-0.0180	0.9346	-0.6552	0.0260	-0.7858	0.0019	0.1306	0.7514
Left caudal middle frontal	1.9236	0.0000	1.8492	0.0000	0.0744	0.8436	0.8484	0.0000	0.7112	0.0000	0.1372	0.5002
Left cuneus	1.9740	0.0000	1.7996	0.0000	0.1744	0.7477	0.3882	0.1259	0.1013	0.6555	0.2869	0.3095
Left entorhinal	1.8378	0.0000	1.7701	0.0000	0.0678	0.9266	1.1837	0.0000	1.0774	0.0000	0.1063	0.8054
Left fusiform	1.7638	0.0000	1.9458	0.0000	-0.1820	0.2393	0.4415	0.0001	0.5395	0.0000	-0.0980	0.4299
Left inferior parietal	1.9768	0.0000	1.9377	0.0000	0.0390	0.9052	0.3429	0.0136	0.1963	0.1017	0.1466	0.3095
Left inferior temporal	1.8428	0.0000	1.9627	0.0000	-0.1199	0.6928	0.4911	0.0001	0.5251	0.0000	-0.0340	0.8483

Left isthmus cingulate	1.8552	0.0000	1.8964	0.0000	-0.0412	0.9266	0.0950	0.6007	0.0118	0.9434	0.0832	0.7444
Left lateral occipital	2.0332	0.0000	1.9843	0.0000	0.0489	0.8609	0.3517	0.0146	0.1945	0.1163	0.1572	0.3095
Left lateral orbital frontal	1.9082	0.0000	1.8679	0.0000	0.0402	0.8609	0.1637	0.1153	0.0054	0.9497	0.1583	0.1242
Left lingual	2.0072	0.0000	1.8599	0.0000	0.1473	0.6839	0.9433	0.0000	0.7208	0.0000	0.2225	0.2258
Left medial orbital frontal	1.9356	0.0000	1.9570	0.0000	-0.0214	0.9346	0.4786	0.0000	0.3987	0.0000	0.0799	0.5403
Left middle temporal	1.8490	0.0000	1.8947	0.0000	-0.0457	0.8609	0.3647	0.0005	0.3102	0.0006	0.0544	0.7152
Left parahippocampal	1.9127	0.0000	1.8470	0.0000	0.0658	0.8609	0.7746	0.0003	0.6349	0.0005	0.1396	0.6058
Left paracentral	2.0044	0.0000	1.9366	0.0000	0.0678	0.8594	0.8220	0.0000	0.6670	0.0000	0.1550	0.4299
Left pars opercularis	1.8196	0.0000	1.8690	0.0000	-0.0495	0.8609	0.7256	0.0000	0.7159	0.0000	0.0096	0.9733
Left pars orbitalis	1.9239	0.0000	1.9394	0.0000	-0.0155	0.9346	0.7586	0.0000	0.6958	0.0000	0.0628	0.7738
Left pars triangularis	1.7369	0.0000	1.8909	0.0000	-0.1540	0.5964	0.7701	0.0000	0.8615	0.0000	-0.0914	0.6058
Left pericalcarine	0.9448	0.0002	0.4710	0.0000	0.4738	0.5964	-1.3462	0.0055	-1.9918	0.0000	0.6455	0.1358
Left postcentral	1.8029	0.0000	1.8241	0.0000	-0.0212	0.9346	-0.4018	0.0214	-0.5557	0.0002	0.1539	0.4384
Left posterior cingulate	1.7900	0.0000	1.8457	0.0000	-0.0557	0.8609	-0.0427	0.8016	-0.1062	0.4669	0.0635	0.7738
Left precentral	1.8229	0.0000	1.8940	0.0000	-0.0711	0.8090	0.4145	0.0003	0.3988	0.0001	0.0157	0.9082
Left precuneus	1.9271	0.0000	2.0115	0.0000	-0.0844	0.7552	0.5475	0.0000	0.5384	0.0000	0.0091	0.9565
Left rostral anterior cingulate	1.7252	0.0000	1.7503	0.0000	-0.0251	0.9346	-0.0379	0.8338	-0.1331	0.3845	0.0951	0.6996
Left rostral middle frontal	1.9224	0.0000	1.9410	0.0000	-0.0187	0.9346	0.4904	0.0000	0.4100	0.0000	0.0804	0.5752
Left superior frontal	1.9293	0.0000	1.9382	0.0000	-0.0088	0.9346	0.6143	0.0000	0.5326	0.0000	0.0817	0.5265
Left superior parietal	1.8742	0.0000	1.9961	0.0000	-0.1218	0.7351	0.3931	0.0071	0.4094	0.0007	-0.0164	0.9450
Left superior temporal	1.6991	0.0000	1.9823	0.0000	-0.2832	0.0288	0.1756	0.1162	0.3517	0.0002	-0.1761	0.1144

Left supramarginal	1.6176	0.0000	1.9137	0.0000	-0.2961	0.0913	0.1393	0.3850	0.3344	0.0122	-0.1951	0.2633
Left frontal pole	2.0863	0.0000	2.1208	0.0000	-0.0346	0.9346	1.4203	0.0000	1.4153	0.0000	0.0051	0.9739
Left temporal pole	2.0783	0.0000	1.9801	0.0000	0.0982	0.8226	0.9912	0.0000	0.8186	0.0000	0.1726	0.4827
Left transverse temporal	1.4008	0.0000	1.8123	0.0000	-0.4115	0.0634	-0.6536	0.0069	-0.3877	0.0752	-0.2659	0.2862
Left insula	1.8711	0.0000	1.9298	0.0000	-0.0587	0.8090	0.4265	0.0000	0.3833	0.0000	0.0432	0.7738
Right banks superior temporal sulcus	1.8812	0.0000	1.9102	0.0000	-0.0290	0.9346	0.7246	0.0001	0.6790	0.0000	0.0456	0.8483
Right caudal anterior cingulate	1.6096	0.0000	1.4455	0.0000	0.1641	0.8090	-0.6802	0.0355	-0.9849	0.0005	0.3047	0.4034
Right caudal middle frontal	1.6266	0.0000	1.8372	0.0000	-0.2106	0.5881	0.5236	0.0051	0.6642	0.0000	-0.1406	0.5265
Right cuneus	1.7010	0.0000	1.8802	0.0000	-0.1791	0.7351	-0.1007	0.6853	-0.0541	0.7997	-0.0466	0.8902
Right entorhinal	1.9089	0.0000	1.7623	0.0000	0.1466	0.8090	0.8448	0.0028	0.6284	0.0079	0.2163	0.5403
Right fusiform	1.8102	0.0000	1.9164	0.0000	-0.1062	0.7351	0.3725	0.0024	0.3818	0.0003	-0.0093	0.9565
Right inferior parietal	1.8844	0.0000	1.9482	0.0000	-0.0638	0.8575	0.5598	0.0001	0.5377	0.0000	0.0221	0.8902
Right inferior temporal	1.9434	0.0000	1.9317	0.0000	0.0118	0.9346	0.5036	0.0004	0.3970	0.0010	0.1067	0.5403
Right isthmus cingulate	1.8328	0.0000	1.8758	0.0000	-0.0430	0.9346	-0.0390	0.8421	-0.1209	0.4947	0.0819	0.7738
Right lateral occipital	2.1475	0.0000	1.9791	0.0000	0.1683	0.5964	0.5880	0.0002	0.3155	0.0185	0.2726	0.0856
Right lateral orbital frontal	1.8678	0.0000	1.9265	0.0000	-0.0587	0.8436	0.1280	0.2478	0.0589	0.5421	0.0690	0.6244
Right lingual	1.8871	0.0000	1.9409	0.0000	-0.0538	0.8609	0.5003	0.0064	0.4613	0.0032	0.0390	0.8774
Right medial orbital frontal	1.9137	0.0000	1.9916	0.0000	-0.0779	0.7552	0.4800	0.0000	0.4494	0.0000	0.0306	0.8261
Right middle temporal	1.9108	0.0000	1.8789	0.0000	0.0319	0.9052	0.5548	0.0000	0.4280	0.0000	0.1268	0.2965
Right parahippocampal	1.8309	0.0000	1.8641	0.0000	-0.0331	0.9346	0.8944	0.0000	0.8607	0.0000	0.0338	0.8902

Right paracentral	1.9315	0.0000	1.9449	0.0000	-0.0133	0.9346	0.8735	0.0000	0.8125	0.0000	0.0610	0.7738
Right pars opercularis	1.8165	0.0000	1.8374	0.0000	-0.0209	0.9346	0.4862	0.0049	0.4268	0.0032	0.0594	0.8054
Right pars orbitalis	1.8962	0.0000	1.9688	0.0000	-0.0726	0.8090	0.8055	0.0000	0.8015	0.0000	0.0040	0.9739
Right pars triangularis	1.7988	0.0000	1.8979	0.0000	-0.0991	0.8090	0.7034	0.0000	0.7336	0.0000	-0.0302	0.8902
Right pericalcarine	0.8159	0.0006	0.6572	0.0000	0.1587	0.8594	-2.0126	0.0000	-2.3805	0.0000	0.3679	0.4299
Right postcentral	1.6952	0.0000	1.8571	0.0000	-0.1619	0.7351	-0.4693	0.0106	-0.4657	0.0027	-0.0036	0.9739
Right posterior cingulate	1.9335	0.0000	1.7803	0.0000	0.1532	0.6928	0.3478	0.0466	0.0961	0.5421	0.2517	0.1358
Right precentral	1.7593	0.0000	1.8859	0.0000	-0.1266	0.5997	0.4440	0.0002	0.4789	0.0000	-0.0350	0.8410
Right precuneus	1.9982	0.0000	2.0194	0.0000	-0.0212	0.9346	0.5052	0.0000	0.4270	0.0000	0.0782	0.5669
Right rostral anterior cingulate	1.7450	0.0000	1.6772	0.0000	0.0678	0.8609	0.3915	0.0787	0.2350	0.2327	0.1565	0.5403
Right rostral middle frontal	2.0419	0.0000	1.9313	0.0000	0.1106	0.7351	0.6559	0.0000	0.4369	0.0000	0.2190	0.0856
Right superior frontal	1.8510	0.0000	1.9208	0.0000	-0.0698	0.8090	0.4847	0.0000	0.4467	0.0000	0.0380	0.8090
Right superior parietal	1.8856	0.0000	1.9892	0.0000	-0.1037	0.8090	0.4387	0.0014	0.4501	0.0001	-0.0114	0.9565
Right superior temporal	1.6923	0.0000	1.9879	0.0000	-0.2957	0.0177	0.3356	0.0049	0.5394	0.0000	-0.2038	0.0856
Right supramarginal	1.6082	0.0000	1.9074	0.0000	-0.2992	0.0483	0.0825	0.5652	0.2813	0.0161	-0.1988	0.1358
Right frontal pole	2.3509	0.0000	2.1214	0.0000	0.2295	0.5964	1.8560	0.0000	1.6021	0.0000	0.2538	0.2981
Right temporal pole	2.1213	0.0000	1.9809	0.0000	0.1403	0.7351	1.0165	0.0000	0.8081	0.0000	0.2084	0.3095
Right transverse temporal	1.5373	0.0000	1.7628	0.0000	-0.2255	0.5964	-0.4511	0.0713	-0.3626	0.1054	-0.0884	0.7738
Right insula	1.7500	0.0000	1.8948	0.0000	-0.1447	0.5694	0.2999	0.0024	0.3479	0.0000	-0.0480	0.7444

Note: Red text denotes $p<0.05$ (false discovery rate corrected); Regression coefficients (β) and *p*-values are derived from linear mixed effects models.

Supplementary Table 5b. Brain growth from 7 to 13 years in very preterm children born small (SGA) or appropriate for gestational age (AGA) and the difference in trajectories.

**Region**	Unadjusted						Adjusted for total brain tissue volume					
	SGA β	SGA *p*	AGA β	AGA *p*	Growth restriction-by-time β	Growth restriction-by-time *p*	SGA β	SGA *p*	AGA β	AGA *p*	Growth restriction-by-time β	Growth restriction-by-time *p*
Intracranial	0.2294	0.0005	0.1974	0.0000	0.0320	0.9628	0.1072	0.0002	0.1043	0.0000	0.0029	0.9610
Total brain tissue	0.1335	0.0999	0.1025	0.0000	0.0310	0.9628						
Extra-axial cerebrospinal fluid	0.8869	0.0010	0.8831	0.0000	0.0038	0.9867	0.8490	0.0007	0.8654	0.0000	-0.0164	0.9645
Cerebrospinal fluid	0.8315	0.0005	0.8079	0.0000	0.0236	0.9660	0.8234	0.0002	0.8014	0.0000	0.0220	0.9610
Left cortical grey matter	-0.0611	0.6072	-0.0323	0.1103	-0.0288	0.9628	-0.1774	0.0000	-0.1153	0.0000	-0.0621	0.3900
Right cortical grey matter	-0.0465	0.7200	-0.0325	0.1083	-0.0140	0.9628	-0.1615	0.0000	-0.1138	0.0000	-0.0478	0.4703
Left lateral ventricle	0.1246	0.8759	0.2445	0.0265	-0.1199	0.9628	0.0840	0.8402	0.2210	0.0415	-0.1370	0.8937
Right lateral ventricle	0.0235	0.9605	0.2754	0.0128	-0.2519	0.9628	-0.0140	0.9716	0.2531	0.0179	-0.2671	0.8423
Third ventricle	0.2829	0.6283	0.0822	0.4038	0.2007	0.9628	0.2873	0.4106	0.0784	0.4139	0.2089	0.8423
Fourth ventricle	0.3061	0.3842	0.2034	0.0009	0.1026	0.9628	0.2803	0.2086	0.1882	0.0017	0.0921	0.8937
Left white matter	-0.0004	0.9957	-0.0062	0.8460	0.0058	0.9721	-0.1842	0.0018	-0.1450	0.0000	-0.0391	0.8423
Right white matter	0.0162	0.9601	-0.0122	0.7018	0.0284	0.9628	-0.1663	0.0045	-0.1469	0.0000	-0.0194	0.9075
Corpus callosum	0.0682	0.8101	0.0898	0.0217	-0.0215	0.9628	-0.0746	0.5627	0.0153	0.6791	-0.0899	0.8396
Brainstem	0.2599	0.0011	0.3110	0.0000	-0.0510	0.9628	0.1385	0.0139	0.2505	0.0000	-0.1119	0.2863
Left cerebellum	0.0067	0.9601	-0.0278	0.1657	0.0344	0.9628	-0.0928	0.0856	-0.0787	0.0000	-0.0141	0.9160
Right cerebellum	0.0336	0.8115	0.0078	0.7185	0.0258	0.9628	-0.0673	0.2424	-0.0442	0.0042	-0.0230	0.8937
Left thalamus	0.2954	0.0196	0.2569	0.0000	0.0386	0.9628	0.1033	0.2896	0.1608	0.0000	-0.0575	0.8642

Left caudate	-0.0938	0.6072	-0.0456	0.1133	-0.0482	0.9628	-0.2445	0.0050	-0.1185	0.0000	-0.1261	0.3900
Left putamen	-0.0658	0.7379	0.0722	0.0157	-0.1379	0.9628	-0.1812	0.0517	0.0195	0.4799	-0.2007	0.2609
Left pallidum	0.1103	0.5991	0.2608	0.0000	-0.1505	0.9628	-0.0291	0.7823	0.1987	0.0000	-0.2278	0.1733
Left hippocampus	0.0498	0.7957	0.0926	0.0003	-0.0428	0.9628	-0.0884	0.2877	0.0225	0.3342	-0.1110	0.3900
Left amygdala	0.3282	0.0140	0.2063	0.0000	0.1219	0.9628	0.1882	0.0856	0.1336	0.0000	0.0546	0.8742
Left accumbens	-0.1205	0.7261	-0.0429	0.4267	-0.0776	0.9628	-0.2509	0.1619	-0.1050	0.0323	-0.1460	0.7449
Right thalamus	0.1668	0.2774	0.1907	0.0000	-0.0239	0.9628	-0.0290	0.7357	0.0946	0.0000	-0.1236	0.3900
Right caudate	-0.1214	0.4754	-0.0181	0.5451	-0.1033	0.9628	-0.2726	0.0016	-0.0946	0.0001	-0.1780	0.2686
Right putamen	-0.0755	0.6072	0.0552	0.0192	-0.1307	0.9628	-0.1980	0.0045	-0.0002	0.9912	-0.1977	0.0877
Right pallidum	0.2253	0.1859	0.1891	0.0000	0.0362	0.9628	0.0746	0.5194	0.1176	0.0001	-0.0431	0.8937
Right hippocampus	-0.0248	0.8760	0.0401	0.1096	-0.0648	0.9628	-0.1541	0.0460	-0.0202	0.3765	-0.1339	0.3900
Right amygdala	0.2055	0.0999	0.1147	0.0000	0.0908	0.9628	0.0711	0.4325	0.0508	0.0378	0.0203	0.9229
Right accumbens	-0.0803	0.7379	0.0258	0.5090	-0.1060	0.9628	-0.2197	0.0642	-0.0462	0.1832	-0.1735	0.3900
Left banks superior temporal sulcus	-0.1839	0.5139	-0.1745	0.0000	-0.0094	0.9721	-0.3252	0.0150	-0.2486	0.0000	-0.0766	0.8742
Left caudal anterior cingulate	0.2502	0.5139	0.1730	0.0042	0.0772	0.9628	0.0576	0.7849	0.0737	0.2066	-0.0161	0.9645
Left caudal middle frontal	-0.0151	0.9601	0.0455	0.2215	-0.0606	0.9628	-0.1131	0.3744	-0.0055	0.8979	-0.1075	0.7091
Left cuneus	-0.2262	0.5162	-0.0772	0.1737	-0.1490	0.9628	-0.3781	0.0325	-0.1502	0.0028	-0.2280	0.4761
Left entorhinal	0.2258	0.6072	0.0144	0.8460	0.2115	0.9628	0.1643	0.4789	-0.0216	0.7488	0.1858	0.7433
Left fusiform	-0.0061	0.9605	-0.0235	0.4004	0.0174	0.9628	-0.1290	0.0938	-0.0842	0.0001	-0.0448	0.8742
Left inferior parietal	-0.2044	0.2274	-0.1517	0.0000	-0.0527	0.9628	-0.3632	0.0001	-0.2266	0.0000	-0.1365	0.3900
Left inferior temporal	-0.1087	0.6072	-0.0221	0.5019	-0.0866	0.9628	-0.2364	0.0060	-0.0879	0.0003	-0.1485	0.3900

Left isthmus cingulate	-0.0978	0.7261	-0.0650	0.1209	-0.0327	0.9628	-0.2644	0.0302	-0.1479	0.0000	-0.1165	0.6857
Left lateral occipital	-0.1843	0.3177	-0.1505	0.0000	-0.0338	0.9628	-0.3432	0.0006	-0.2303	0.0000	-0.1130	0.5865
Left lateral orbital frontal	-0.0494	0.8101	0.0349	0.2283	-0.0844	0.9628	-0.2231	0.0018	-0.0483	0.0193	-0.1749	0.1733
Left lingual	-0.0740	0.7588	-0.0223	0.5651	-0.0517	0.9628	-0.1762	0.1554	-0.0736	0.0293	-0.1026	0.7433
Left medial orbital frontal	-0.0362	0.8759	-0.0620	0.0235	0.0258	0.9628	-0.1823	0.0180	-0.1273	0.0000	-0.0550	0.8423
Left middle temporal	-0.0843	0.6283	-0.0119	0.7018	-0.0724	0.9628	-0.2285	0.0018	-0.0825	0.0001	-0.1460	0.2863
Left parahippocampal	0.0319	0.9474	0.0541	0.2682	-0.0222	0.9628	-0.0739	0.6451	-0.0005	0.9912	-0.0734	0.8742
Left paracentral	0.0147	0.9601	-0.0929	0.0124	0.1075	0.9628	-0.1044	0.4106	-0.1493	0.0000	0.0448	0.8937
Left pars opercularis	-0.0151	0.9601	0.0056	0.8884	-0.0207	0.9628	-0.1111	0.3831	-0.0475	0.1702	-0.0636	0.8742
Left pars orbitalis	-0.1996	0.2914	-0.1262	0.0003	-0.0734	0.9628	-0.3121	0.0055	-0.1800	0.0000	-0.1321	0.5729
Left pars triangularis	0.0220	0.9474	-0.0594	0.0569	0.0814	0.9628	-0.0695	0.5171	-0.1061	0.0001	0.0367	0.8937
Left pericalcarine	0.2190	0.7200	0.5264	0.0000	-0.3074	0.9628	-0.0157	0.9716	0.4154	0.0000	-0.4311	0.3900
Left postcentral	0.0862	0.7588	-0.0258	0.5670	0.1120	0.9628	-0.1360	0.2918	-0.1297	0.0002	-0.0063	0.9645
Left posterior cingulate	0.0796	0.7588	0.0052	0.8957	0.0744	0.9628	-0.0935	0.4139	-0.0818	0.0076	-0.0117	0.9610
Left precentral	0.0867	0.6283	0.0816	0.0040	0.0051	0.9721	-0.0442	0.5894	0.0139	0.5480	-0.0581	0.8396
Left precuneus	-0.1504	0.3062	-0.1406	0.0000	-0.0098	0.9660	-0.2860	0.0004	-0.2055	0.0000	-0.0805	0.6837
Left rostral anterior cingulate	0.1844	0.4454	0.1388	0.0005	0.0456	0.9628	0.0248	0.8493	0.0601	0.0901	-0.0353	0.9160
Left rostral middle frontal	-0.0991	0.6072	0.0006	0.9815	-0.0997	0.9628	-0.2353	0.0045	-0.0621	0.0088	-0.1732	0.2686
Left superior frontal	-0.0476	0.7975	0.0095	0.7349	-0.0570	0.9628	-0.1731	0.0180	-0.0513	0.0156	-0.1218	0.3900
Left superior parietal	-0.1215	0.6072	-0.1803	0.0000	0.0588	0.9628	-0.2658	0.0124	-0.2504	0.0000	-0.0153	0.9610
Left superior temporal	-0.0092	0.9601	-0.0706	0.0157	0.0614	0.9628	-0.1540	0.0517	-0.1418	0.0000	-0.0123	0.9610

Left supramarginal	0.0055	0.9682	-0.0863	0.0182	0.0918	0.9628	-0.1367	0.2351	-0.1574	0.0000	0.0207	0.9479
Left frontal pole	-0.4194	0.0139	-0.3702	0.0000	-0.0491	0.9628	-0.4794	0.0008	-0.4034	0.0000	-0.0760	0.8742
Left temporal pole	-0.1763	0.5913	-0.1290	0.0055	-0.0473	0.9628	-0.2751	0.0623	-0.1745	0.0000	-0.1007	0.8423
Left transverse temporal	-0.0183	0.9601	-0.1038	0.0258	0.0855	0.9628	-0.2154	0.1702	-0.1970	0.0000	-0.0184	0.9610
Left insula	-0.1919	0.0999	-0.0262	0.3399	-0.1658	0.9628	-0.3324	0.0000	-0.0949	0.0000	-0.2375	0.0271
Right banks superior temporal sulcus	-0.2369	0.2444	-0.1652	0.0000	-0.0717	0.9628	-0.3458	0.0057	-0.2236	0.0000	-0.1222	0.6857
Right caudal anterior cingulate	0.2183	0.6072	0.1861	0.0059	0.0322	0.9628	0.0067	0.9716	0.0729	0.2529	-0.0663	0.9160
Right caudal middle frontal	0.1869	0.3957	0.0559	0.1638	0.1309	0.9628	0.0865	0.5222	0.0019	0.9758	0.0846	0.8423
Right cuneus	0.1201	0.7376	-0.1520	0.0042	0.2721	0.9628	-0.0626	0.7357	-0.2351	0.0000	0.1726	0.6837
Right entorhinal	-0.0990	0.8150	0.1376	0.0235	-0.2366	0.9628	-0.1986	0.3714	0.0878	0.1408	-0.2864	0.3900
Right fusiform	0.0357	0.8759	-0.0300	0.3399	0.0656	0.9628	-0.0997	0.2541	-0.0946	0.0001	-0.0051	0.9645
Right inferior parietal	-0.1742	0.3116	-0.0839	0.0115	-0.0903	0.9628	-0.3001	0.0018	-0.1478	0.0000	-0.1523	0.3900
Right inferior temporal	-0.0918	0.7013	0.0012	0.9789	-0.0930	0.9628	-0.2287	0.0235	-0.0641	0.0278	-0.1646	0.3900
Right isthmus cingulate	-0.1705	0.5991	-0.1210	0.0101	-0.0495	0.9628	-0.3524	0.0108	-0.2141	0.0000	-0.1383	0.6837
Right lateral occipital	-0.2055	0.2965	-0.1784	0.0000	-0.0271	0.9628	-0.3542	0.0011	-0.2508	0.0000	-0.1034	0.6857
Right lateral orbital frontal	-0.0095	0.9601	-0.0607	0.0342	0.0512	0.9628	-0.1852	0.0157	-0.1457	0.0000	-0.0395	0.8742
Right lingual	-0.1012	0.7013	-0.1289	0.0007	0.0277	0.9628	-0.2342	0.0633	-0.2009	0.0000	-0.0334	0.9160
Right medial orbital frontal	-0.0936	0.5991	-0.0909	0.0003	-0.0027	0.9818	-0.2410	0.0006	-0.1592	0.0000	-0.0818	0.5729
Right middle temporal	-0.0663	0.7200	-0.0254	0.3699	-0.0409	0.9628	-0.1986	0.0066	-0.0910	0.0000	-0.1076	0.3900
Right parahippocampal	-0.0200	0.9601	0.0716	0.0555	-0.0916	0.9628	-0.1067	0.4139	0.0290	0.4249	-0.1358	0.6030

Right paracentral	0.0385	0.8759	-0.0611	0.0672	0.0996	0.9628	-0.0606	0.5894	-0.1108	0.0002	0.0502	0.8742
Right pars opercularis	0.0504	0.8759	0.0221	0.5780	0.0283	0.9628	-0.0745	0.5627	-0.0405	0.2529	-0.0339	0.9160
Right pars orbitalis	-0.0318	0.8760	-0.0736	0.0182	0.0417	0.9628	-0.1357	0.1702	-0.1230	0.0000	-0.0127	0.9610
Right pars triangularis	-0.0541	0.8492	-0.0410	0.2843	-0.0131	0.9660	-0.1557	0.1925	-0.0950	0.0033	-0.0607	0.8742
Right pericalcarine	0.3901	0.4156	0.6320	0.0000	-0.2419	0.9628	0.0864	0.7823	0.4927	0.0000	-0.4063	0.3900
Right postcentral	0.0523	0.8759	-0.0607	0.1737	0.1131	0.9628	-0.1577	0.2424	-0.1607	0.0000	0.0030	0.9790
Right posterior cingulate	0.0438	0.8759	0.0520	0.1870	-0.0082	0.9721	-0.1032	0.4139	-0.0247	0.4855	-0.0785	0.8423
Right precentral	0.1586	0.3062	0.1231	0.0000	0.0355	0.9628	0.0420	0.6451	0.0625	0.0109	-0.0205	0.9229
Right precuneus	-0.2634	0.0196	-0.1481	0.0000	-0.1154	0.9628	-0.4116	0.0000	-0.2188	0.0000	-0.1928	0.1733
Right rostral anterior cingulate	0.1524	0.6072	0.1147	0.0142	0.0376	0.9628	0.0238	0.8819	0.0525	0.2384	-0.0286	0.9479
Right rostral middle frontal	-0.0798	0.7013	-0.0312	0.3096	-0.0487	0.9628	-0.2200	0.0110	-0.0953	0.0001	-0.1247	0.3900
Right superior frontal	0.0694	0.7013	0.0284	0.3096	0.0409	0.9628	-0.0592	0.4912	-0.0303	0.1981	-0.0289	0.8937
Right superior parietal	-0.1275	0.6072	-0.1561	0.0000	0.0286	0.9628	-0.2595	0.0124	-0.2224	0.0000	-0.0371	0.8937
Right superior temporal	0.0290	0.8760	-0.0748	0.0086	0.1038	0.9628	-0.0986	0.2470	-0.1387	0.0000	0.0401	0.8742
Right supramarginal	-0.0258	0.9378	-0.0518	0.1220	0.0260	0.9628	-0.1707	0.0807	-0.1250	0.0000	-0.0457	0.8742
Right frontal pole	-0.6010	0.0005	-0.3933	0.0000	-0.2077	0.9628	-0.6454	0.0000	-0.4227	0.0000	-0.2227	0.3900
Right temporal pole	-0.2909	0.1674	-0.1056	0.0160	-0.1853	0.9628	-0.3908	0.0042	-0.1585	0.0000	-0.2323	0.3900
Right transverse temporal	-0.0810	0.8150	-0.1144	0.0192	0.0334	0.9628	-0.2723	0.0906	-0.2092	0.0000	-0.0631	0.8937
Right insula	0.0315	0.8759	0.0604	0.0192	-0.0289	0.9628	-0.1027	0.1619	-0.0029	0.9070	-0.0999	0.3900

Note: Red text denotes $p<0.05$ (false discovery rate corrected); Regression coefficients (β) and *p*-values are derived from linear mixed effects models.

Supplementary Table 6a. Brain growth from term-equivalent to 7 years in very preterm children born with or without bronchopulmonary dysplasia (BPD) and the difference in trajectories.

Region	Unadjusted						Adjusted for total brain tissue volume					
	BPD β	BPD *p*	No BPD β	No BPD *p*	BPD-by-time β	BPD-by-time *p*	BPD β	BPD *p*	No BPD β	No BPD *p*	BPD-by-time β	BPD-by-time *p*
Intracranial	1.8767	0.0000	1.8332	0.0000	0.0435	0.7203	0.1145	0.0072	0.1037	0.0111	0.0108	0.8688
Total brain tissue	1.9257	0.0000	1.8901	0.0000	0.0356	0.8605						
Extra-axial cerebrospinal fluid	1.0254	0.0000	0.9373	0.0000	0.0881	0.8731	0.4862	0.1398	0.4067	0.1912	0.0795	0.8688
Cerebrospinal fluid	1.0914	0.0000	1.0044	0.0000	0.0870	0.8731	0.8792	0.0072	0.7965	0.0111	0.0826	0.8688
Left cortical grey matter	1.9800	0.0000	1.9545	0.0000	0.0254	0.8731	0.3927	0.0000	0.4022	0.0000	-0.0095	0.8688
Right cortical grey matter	1.9816	0.0000	1.9582	0.0000	0.0234	0.8731	0.4425	0.0000	0.4558	0.0000	-0.0133	0.8688
Left lateral ventricle	0.5249	0.0010	0.5445	0.0000	-0.0196	0.9672	-0.1501	0.7983	-0.1332	0.8079	-0.0169	0.9325
Right lateral ventricle	0.6877	0.0000	0.6560	0.0000	0.0317	0.9672	0.1030	0.8533	0.0666	0.9046	0.0364	0.9325
Third ventricle	1.2661	0.0000	1.2493	0.0000	0.0168	0.9672	1.1734	0.0117	1.1410	0.0114	0.0324	0.9325
Fourth ventricle	1.7030	0.0000	1.6362	0.0000	0.0669	0.8731	1.4894	0.0000	1.4183	0.0000	0.0710	0.8688
Left white matter	1.8997	0.0000	1.8521	0.0000	0.0476	0.8605	-0.7504	0.0000	-0.7356	0.0000	-0.0148	0.8688
Right white matter	1.9102	0.0000	1.8630	0.0000	0.0472	0.8605	-0.6350	0.0000	-0.6177	0.0000	-0.0172	0.8688
Corpus callosum	1.8032	0.0000	1.8544	0.0000	-0.0512	0.8731	0.1717	0.2729	0.2423	0.0962	-0.0706	0.8019
Brainstem	1.7218	0.0000	1.7671	0.0000	-0.0453	0.7548	0.3918	0.0000	0.4463	0.0000	-0.0545	0.4364
Left cerebellum	1.9459	0.0000	1.9896	0.0000	-0.0437	0.7203	0.8499	0.0000	0.9034	0.0000	-0.0535	0.3906
Right cerebellum	1.9441	0.0000	1.9606	0.0000	-0.0165	0.8956	0.8313	0.0000	0.8577	0.0000	-0.0264	0.8125
Left thalamus	1.6859	0.0000	1.6699	0.0000	0.0160	0.9345	-0.4278	0.0006	-0.4236	0.0004	-0.0042	0.9325
Left caudate	1.9251	0.0000	1.9007	0.0000	0.0243	0.8956	0.2669	0.0453	0.2466	0.0564	0.0202	0.8688

Left putamen	1.9319	0.0000	1.8559	0.0000	0.0760	0.7203	0.6515	0.0000	0.5924	0.0000	0.0591	0.8019
Left pallidum	1.7691	0.0000	1.7402	0.0000	0.0288	0.8956	0.3812	0.0021	0.3590	0.0023	0.0222	0.8688
Left hippocampus	1.9250	0.0000	1.8346	0.0000	0.0904	0.6054	0.4648	0.0000	0.3808	0.0004	0.0840	0.3585
Left amygdala	1.8834	0.0000	1.7466	0.0000	0.1368	0.3463	0.3877	0.0051	0.2538	0.0562	0.1339	0.2223
Left accumbens	1.9246	0.0000	1.7996	0.0000	0.1251	0.7203	0.4059	0.0504	0.2980	0.1309	0.1079	0.8019
Right thalamus	1.6858	0.0000	1.7161	0.0000	-0.0303	0.8731	-0.4310	0.0004	-0.3864	0.0010	-0.0447	0.8019
Right caudate	1.9048	0.0000	1.8973	0.0000	0.0075	0.9672	0.2907	0.0246	0.2801	0.0248	0.0106	0.9325
Right putamen	1.9511	0.0000	1.8786	0.0000	0.0726	0.7203	0.6721	0.0000	0.6149	0.0000	0.0572	0.6008
Right pallidum	1.7676	0.0000	1.7682	0.0000	-0.0006	0.9922	0.1772	0.1973	0.1923	0.1342	-0.0151	0.9325
Right hippocampus	1.9232	0.0000	1.8904	0.0000	0.0328	0.8731	0.6559	0.0000	0.6390	0.0000	0.0169	0.8688
Right amygdala	1.9437	0.0000	1.8481	0.0000	0.0956	0.6054	0.4872	0.0000	0.4095	0.0002	0.0778	0.4892
Right accumbens	1.9141	0.0000	1.8531	0.0000	0.0611	0.8605	0.3996	0.0106	0.3409	0.0235	0.0587	0.8125
Left banks superior temporal sulcus	1.8855	0.0000	1.9082	0.0000	-0.0227	0.9345	0.3356	0.0438	0.3761	0.0179	-0.0405	0.8688
Left caudal anterior cingulate	1.4160	0.0000	1.5085	0.0000	-0.0925	0.8731	-0.7639	0.0040	-0.6293	0.0133	-0.1346	0.8019
Left caudal middle frontal	1.8966	0.0000	1.8359	0.0000	0.0607	0.8605	0.7823	0.0000	0.7299	0.0000	0.0524	0.8340
Left cuneus	1.8444	0.0000	1.8031	0.0000	0.0413	0.8956	0.2409	0.3004	0.2308	0.2923	0.0101	0.9325
Left entorhinal	1.7816	0.0000	1.7742	0.0000	0.0074	0.9672	1.1673	0.0000	1.1574	0.0000	0.0099	0.9325
Left fusiform	1.8816	0.0000	1.9552	0.0000	-0.0736	0.7203	0.4847	0.0000	0.5781	0.0000	-0.0934	0.2223
Left inferior parietal	1.9864	0.0000	1.9195	0.0000	0.0669	0.7548	0.2599	0.0383	0.2191	0.0670	0.0408	0.8510
Left inferior temporal	1.9809	0.0000	1.9392	0.0000	0.0416	0.8731	0.4910	0.0000	0.4623	0.0000	0.0287	0.8688
Left isthmus cingulate	1.9600	0.0000	1.8617	0.0000	0.0983	0.7203	0.0722	0.6596	0.0011	0.9941	0.0711	0.8019

Left lateral occipital	2.0253	0.0000	1.9713	0.0000	0.0539	0.8731	0.2642	0.0421	0.2302	0.0639	0.0340	0.8688
Left lateral orbital frontal	1.8746	0.0000	1.8714	0.0000	0.0032	0.9672	0.0633	0.5159	0.0794	0.3798	-0.0161	0.8688
Left lingual	1.8376	0.0000	1.8895	0.0000	-0.0519	0.8731	0.8329	0.0000	0.8975	0.0000	-0.0647	0.8019
Left medial orbital frontal	1.9654	0.0000	1.9518	0.0000	0.0136	0.9345	0.4482	0.0000	0.4527	0.0000	-0.0045	0.9325
Left middle temporal	1.9090	0.0000	1.8828	0.0000	0.0262	0.8956	0.3244	0.0006	0.3196	0.0004	0.0048	0.9325
Left parahippocampal	1.9184	0.0000	1.8218	0.0000	0.0966	0.7548	0.7237	0.0001	0.6407	0.0004	0.0830	0.8019
Left paracentral	2.0209	0.0000	1.9051	0.0000	0.1158	0.6801	0.8305	0.0000	0.7301	0.0000	0.1004	0.5459
Left pars opercularis	1.8433	0.0000	1.8771	0.0000	-0.0338	0.8956	0.6838	0.0000	0.7290	0.0000	-0.0453	0.8688
Left pars orbitalis	1.9962	0.0000	1.9110	0.0000	0.0852	0.7203	0.7336	0.0000	0.6619	0.0000	0.0717	0.8019
Left pars triangularis	1.8708	0.0000	1.8829	0.0000	-0.0121	0.9672	0.8430	0.0000	0.8658	0.0000	-0.0228	0.8688
Left pericalcarine	0.7628	0.0000	0.3956	0.0000	0.3672	0.3463	-1.5102	0.0006	-1.8456	0.0000	0.3354	0.3585
Left postcentral	1.8189	0.0000	1.8277	0.0000	-0.0089	0.9672	-0.4273	0.0060	-0.3851	0.0100	-0.0423	0.8688
Left posterior cingulate	1.8533	0.0000	1.8361	0.0000	0.0172	0.9445	-0.0697	0.6537	-0.0590	0.6777	-0.0106	0.9325
Left precentral	1.8848	0.0000	1.8905	0.0000	-0.0057	0.9672	0.4217	0.0000	0.4429	0.0000	-0.0212	0.8688
Left precuneus	2.0267	0.0000	1.9947	0.0000	0.0319	0.8731	0.5664	0.0000	0.5562	0.0000	0.0102	0.9325
Left rostral anterior cingulate	1.7773	0.0000	1.7349	0.0000	0.0424	0.8731	-0.0703	0.6615	-0.0833	0.5859	0.0130	0.9325
Left rostral middle frontal	1.9267	0.0000	1.9468	0.0000	-0.0201	0.8966	0.3928	0.0001	0.4313	0.0000	-0.0385	0.8340
Left superior frontal	1.9880	0.0000	1.9135	0.0000	0.0745	0.7203	0.6055	0.0000	0.5434	0.0000	0.0621	0.5788
Left superior parietal	2.0299	0.0000	1.9661	0.0000	0.0639	0.8605	0.4435	0.0005	0.4112	0.0007	0.0323	0.8688
Left superior temporal	1.9250	0.0000	1.9774	0.0000	-0.0524	0.8605	0.2679	0.0077	0.3490	0.0003	-0.0811	0.3906
Left supramarginal	1.8354	0.0000	1.9186	0.0000	-0.0832	0.7203	0.2669	0.0532	0.3751	0.0046	-0.1082	0.3906

Left frontal pole	2.1414	0.0000	2.1058	0.0000	0.0356	0.8956	1.4059	0.0000	1.3754	0.0000	0.0305	0.8688
Left temporal pole	1.9581	0.0000	2.0052	0.0000	-0.0471	0.8731	0.8694	0.0000	0.9523	0.0000	-0.0828	0.8019
Left transverse temporal	1.7843	0.0000	1.7783	0.0000	0.0061	0.9672	-0.5132	0.0228	-0.4719	0.0299	-0.0412	0.8688
Left insula	1.9106	0.0000	1.9331	0.0000	-0.0225	0.8956	0.3434	0.0003	0.3835	0.0000	-0.0401	0.8019
Right banks superior temporal sulcus	1.8032	0.0000	1.9604	0.0000	-0.1573	0.3463	0.6562	0.0000	0.8227	0.0000	-0.1666	0.2209
Right caudal anterior cingulate	1.5089	0.0000	1.4364	0.0000	0.0725	0.8731	-0.8090	0.0060	-0.8640	0.0022	0.0550	0.8688
Right caudal middle frontal	1.8372	0.0000	1.8116	0.0000	0.0255	0.9057	0.6478	0.0001	0.6329	0.0001	0.0148	0.9325
Right cuneus	1.9565	0.0000	1.8241	0.0000	0.1324	0.7203	0.0085	0.9657	-0.0839	0.6777	0.0924	0.8019
Right entorhinal	1.8822	0.0000	1.7229	0.0000	0.1593	0.7203	0.6770	0.0060	0.5345	0.0235	0.1426	0.7658
Right fusiform	1.9156	0.0000	1.9051	0.0000	0.0106	0.9672	0.3860	0.0004	0.4062	0.0001	-0.0202	0.8688
Right inferior parietal	1.9570	0.0000	1.9371	0.0000	0.0200	0.9057	0.5351	0.0000	0.5309	0.0000	0.0042	0.9325
Right inferior temporal	2.0412	0.0000	1.8793	0.0000	0.1619	0.2062	0.4642	0.0002	0.3276	0.0056	0.1366	0.2209
Right isthmus cingulate	1.9170	0.0000	1.8514	0.0000	0.0655	0.8731	-0.0445	0.8086	-0.0950	0.5859	0.0505	0.8688
Right lateral occipital	2.0240	0.0000	1.9793	0.0000	0.0447	0.8731	0.4564	0.0014	0.4366	0.0014	0.0198	0.9126
Right lateral orbital frontal	1.9073	0.0000	1.9299	0.0000	-0.0226	0.8956	0.1019	0.3009	0.1373	0.1357	-0.0354	0.8340
Right lingual	1.8848	0.0000	1.9634	0.0000	-0.0786	0.7548	0.5116	0.0016	0.5998	0.0001	-0.0882	0.7658
Right medial orbital frontal	1.9737	0.0000	1.9918	0.0000	-0.0181	0.8966	0.4279	0.0000	0.4630	0.0000	-0.0350	0.8125
Right middle temporal	1.8877	0.0000	1.8792	0.0000	0.0085	0.9672	0.4980	0.0000	0.5076	0.0000	-0.0095	0.9325
Right parahippocampal	1.8464	0.0000	1.8695	0.0000	-0.0231	0.9057	0.8670	0.0000	0.9112	0.0000	-0.0441	0.8688
Right paracentral	1.9999	0.0000	1.9176	0.0000	0.0823	0.7203	0.9243	0.0000	0.8545	0.0000	0.0698	0.8019

Right pars opercularis	1.8355	0.0000	1.8369	0.0000	-0.0014	0.9922	0.4774	0.0017	0.4969	0.0006	-0.0195	0.9314
Right pars orbitalis	2.0108	0.0000	1.9403	0.0000	0.0705	0.7203	0.8302	0.0000	0.7815	0.0000	0.0488	0.8125
Right pars triangularis	1.8861	0.0000	1.8929	0.0000	-0.0067	0.9672	0.7591	0.0000	0.7760	0.0000	-0.0169	0.9325
Right pericalcarine	0.9666	0.0000	0.5336	0.0000	0.4330	0.1588	-2.0033	0.0000	-2.4059	0.0000	0.4026	0.2209
Right postcentral	1.7972	0.0000	1.8700	0.0000	-0.0729	0.8605	-0.3947	0.0120	-0.2855	0.0587	-0.1092	0.5459
Right posterior cingulate	1.7080	0.0000	1.8353	0.0000	-0.1273	0.6054	0.1188	0.4677	0.2614	0.0821	-0.1426	0.2484
Right precentral	1.8613	0.0000	1.8834	0.0000	-0.0221	0.8956	0.4798	0.0000	0.5233	0.0000	-0.0435	0.8125
Right precuneus	2.0042	0.0000	2.0256	0.0000	-0.0214	0.8956	0.4235	0.0000	0.4741	0.0000	-0.0506	0.8019
Right rostral anterior cingulate	1.7172	0.0000	1.6671	0.0000	0.0501	0.8731	0.3621	0.0727	0.3346	0.0825	0.0275	0.9126
Right rostral middle frontal	1.9807	0.0000	1.9213	0.0000	0.0594	0.7548	0.5385	0.0000	0.4961	0.0000	0.0424	0.8125
Right superior frontal	1.9612	0.0000	1.8932	0.0000	0.0680	0.7203	0.5125	0.0000	0.4646	0.0000	0.0478	0.8019
Right superior parietal	2.0008	0.0000	1.9733	0.0000	0.0274	0.8956	0.4522	0.0001	0.4581	0.0000	-0.0060	0.9325
Right superior temporal	1.8680	0.0000	2.0114	0.0000	-0.1434	0.1588	0.4104	0.0001	0.5785	0.0000	-0.1681	0.0034
Right supramarginal	1.8185	0.0000	1.9156	0.0000	-0.0971	0.7203	0.1408	0.2657	0.2611	0.0269	-0.1203	0.2223
Right frontal pole	2.1737	0.0000	2.1233	0.0000	0.0504	0.8731	1.7150	0.0000	1.6536	0.0000	0.0614	0.8688
Right temporal pole	1.9981	0.0000	1.9917	0.0000	0.0064	0.9672	0.9161	0.0000	0.9257	0.0000	-0.0096	0.9325
Right transverse temporal	1.7943	0.0000	1.7231	0.0000	0.0712	0.8731	-0.3502	0.1330	-0.3980	0.0735	0.0478	0.8688
Right insula	1.9233	0.0000	1.8636	0.0000	0.0597	0.7203	0.3530	0.0001	0.3216	0.0001	0.0313	0.8340

Note: Red text denotes $p<0.05$ (false discovery rate corrected); Regression coefficients (β) and *p*-values are derived from linear mixed effects models.

Supplementary Table 6b. Brain growth from 7 to 13 years in very preterm children born with or without bronchopulmonary dysplasia (BPD) and the difference in trajectories.

**Region**	Unadjusted						Adjusted for total brain tissue volume					
	BPD β	BPD *p*	No BPD β	No BPD *p*	BPD-by-time β	BPD-by-time *p*	BPD β	BPD *p*	No BPD β	No BPD *p*	BPD-by-time β	BPD-by-time *p*
Intracranial	0.1524	0.0000	0.2206	0.0000	-0.0682	0.7177	0.0886	0.0000	0.1104	0.0000	-0.0218	0.9320
Total brain tissue	0.0697	0.0559	0.1208	0.0000	-0.0511	0.7177						
Extra-axial cerebrospinal fluid	0.7663	0.0000	0.9291	0.0000	-0.1628	0.7177	0.7535	0.0000	0.8999	0.0000	-0.1463	0.9320
Cerebrospinal fluid	0.6876	0.0000	0.8611	0.0000	-0.1736	0.7177	0.6803	0.0000	0.8476	0.0000	-0.1673	0.9320
Left cortical grey matter	-0.0685	0.0655	-0.0195	0.4439	-0.0490	0.7177	-0.1182	0.0000	-0.1206	0.0000	0.0024	0.9789
Right cortical grey matter	-0.0661	0.0701	-0.0189	0.4439	-0.0472	0.7177	-0.1122	0.0000	-0.1186	0.0000	0.0063	0.9789
Left lateral ventricle	0.1160	0.5910	0.2850	0.0350	-0.1689	0.7718	0.0839	0.6933	0.2474	0.0559	-0.1635	0.9320
Right lateral ventricle	0.1437	0.4986	0.3026	0.0239	-0.1589	0.7718	0.1130	0.5782	0.2698	0.0339	-0.1567	0.9320
Third ventricle	0.0011	0.9940	0.1427	0.2346	-0.1417	0.7718	-0.0198	0.9182	0.1367	0.2167	-0.1565	0.9320
Fourth ventricle	0.1764	0.1225	0.2307	0.0015	-0.0543	0.8463	0.1626	0.1231	0.2184	0.0019	-0.0558	0.9650
Left white matter	-0.0430	0.4608	0.0112	0.7716	-0.0542	0.7235	-0.1375	0.0000	-0.1554	0.0000	0.0179	0.9650
Right white matter	-0.0483	0.4016	0.0081	0.8173	-0.0564	0.7177	-0.1370	0.0000	-0.1546	0.0000	0.0176	0.9650
Corpus callosum	0.0546	0.4955	0.0972	0.0514	-0.0426	0.8029	0.0012	0.9826	0.0055	0.8973	-0.0043	0.9802
Brainstem	0.2879	0.0000	0.3128	0.0000	-0.0249	0.8029	0.2417	0.0000	0.2408	0.0000	0.0008	0.9831
Left cerebellum	-0.0139	0.7195	-0.0316	0.2072	0.0176	0.8378	-0.0515	0.0484	-0.0925	0.0000	0.0410	0.9320
Right cerebellum	0.0011	0.9792	0.0129	0.6202	-0.0117	0.8878	-0.0373	0.1683	-0.0493	0.0064	0.0120	0.9679
Left thalamus	0.2507	0.0000	0.2636	0.0000	-0.0129	0.8995	0.1732	0.0001	0.1487	0.0000	0.0245	0.9650
Left caudate	-0.1031	0.0606	-0.0291	0.4439	-0.0739	0.7177	-0.1747	0.0001	-0.1195	0.0001	-0.0552	0.9320

Left putamen	0.0067	0.9015	0.0821	0.0250	-0.0754	0.7177	-0.0305	0.5456	0.0124	0.7200	-0.0428	0.9320
Left pallidum	0.1839	0.0007	0.2737	0.0000	-0.0898	0.7177	0.1312	0.0074	0.1985	0.0000	-0.0673	0.9320
Left hippocampus	0.0506	0.3231	0.1080	0.0004	-0.0573	0.7177	-0.0061	0.9171	0.0273	0.3125	-0.0334	0.9320
Left amygdala	0.1649	0.0070	0.2418	0.0000	-0.0769	0.7177	0.1071	0.0441	0.1583	0.0000	-0.0512	0.9320
Left accumbens	-0.0858	0.3906	-0.0347	0.6178	-0.0511	0.8029	-0.1314	0.1231	-0.1191	0.0389	-0.0124	0.9789
Right thalamus	0.2027	0.0000	0.1810	0.0000	0.0216	0.8638	0.1255	0.0010	0.0668	0.0103	0.0588	0.9320
Right caudate	-0.0684	0.2256	-0.0119	0.7652	-0.0566	0.7177	-0.1494	0.0008	-0.1005	0.0008	-0.0489	0.9320
Right putamen	0.0171	0.7195	0.0546	0.0643	-0.0374	0.7687	-0.0236	0.5394	-0.0160	0.5288	-0.0076	0.9789
Right pallidum	0.1376	0.0363	0.2149	0.0000	-0.0773	0.7177	0.0831	0.1240	0.1290	0.0004	-0.0459	0.9320
Right hippocampus	0.0123	0.7940	0.0451	0.1418	-0.0328	0.7864	-0.0295	0.4816	-0.0274	0.3089	-0.0021	0.9802
Right amygdala	0.1025	0.0507	0.1311	0.0000	-0.0287	0.8029	0.0594	0.1605	0.0497	0.0859	0.0097	0.9789
Right accumbens	-0.0304	0.6802	0.0367	0.4439	-0.0671	0.7235	-0.0969	0.1038	-0.0496	0.2250	-0.0473	0.9563
Left banks superior temporal sulcus	-0.1818	0.0256	-0.1752	0.0006	-0.0066	0.9641	-0.2296	0.0005	-0.2630	0.0000	0.0334	0.9650
Left caudal anterior cingulate	0.1809	0.0997	0.1759	0.0148	0.0049	0.9724	0.1264	0.2022	0.0542	0.4432	0.0721	0.9650
Left caudal middle frontal	0.0112	0.8625	0.0547	0.2346	-0.0435	0.8029	-0.0278	0.6726	-0.0054	0.8973	-0.0224	0.9723
Left cuneus	-0.1059	0.3231	-0.0859	0.2314	-0.0201	0.9063	-0.1417	0.1102	-0.1760	0.0028	0.0343	0.9723
Left entorhinal	0.0575	0.6599	0.0223	0.7936	0.0352	0.8878	0.0284	0.8368	-0.0137	0.8678	0.0421	0.9723
Left fusiform	-0.0494	0.3298	-0.0132	0.7059	-0.0362	0.7864	-0.0873	0.0193	-0.0894	0.0004	0.0020	0.9802
Left inferior parietal	-0.1924	0.0007	-0.1392	0.0003	-0.0532	0.7642	-0.2405	0.0000	-0.2345	0.0000	-0.0060	0.9789
Left inferior temporal	-0.0540	0.3708	-0.0203	0.6202	-0.0338	0.8029	-0.1017	0.0205	-0.1045	0.0004	0.0028	0.9802
Left isthmus cingulate	-0.0819	0.3088	-0.0620	0.2346	-0.0199	0.8916	-0.1375	0.0213	-0.1664	0.0000	0.0289	0.9650

Left lateral occipital	-0.2024	0.0008	-0.1307	0.0015	-0.0717	0.7177	-0.2557	0.0000	-0.2289	0.0000	-0.0268	0.9650
Left lateral orbital frontal	0.0128	0.7940	0.0326	0.3828	-0.0198	0.8638	-0.0433	0.2439	-0.0690	0.0045	0.0257	0.9650
Left lingual	-0.0810	0.2510	-0.0023	0.9522	-0.0787	0.7177	-0.1119	0.0549	-0.0581	0.1466	-0.0538	0.9320
Left medial orbital frontal	-0.0976	0.0559	-0.0434	0.2259	-0.0542	0.7177	-0.1379	0.0003	-0.1259	0.0000	-0.0120	0.9789
Left middle temporal	-0.0410	0.4522	-0.0091	0.7936	-0.0319	0.8029	-0.0869	0.0182	-0.0988	0.0001	0.0120	0.9789
Left parahippocampal	0.0886	0.3231	0.0355	0.5782	0.0531	0.8029	0.0514	0.5339	-0.0316	0.5527	0.0830	0.9320
Left paracentral	-0.1506	0.0256	-0.0500	0.2905	-0.1006	0.7177	-0.1861	0.0011	-0.1167	0.0026	-0.0694	0.9320
Left pars opercularis	0.0454	0.5033	-0.0189	0.6893	0.0643	0.7235	0.0074	0.9182	-0.0829	0.0358	0.0904	0.9320
Left pars orbitalis	-0.1439	0.0270	-0.1280	0.0019	-0.0159	0.8965	-0.1813	0.0011	-0.1969	0.0000	0.0156	0.9789
Left pars triangularis	-0.0783	0.1860	-0.0426	0.2905	-0.0357	0.8029	-0.1120	0.0219	-0.0995	0.0026	-0.0125	0.9789
Left pericalcarine	0.5162	0.0008	0.4929	0.0000	0.0233	0.9337	0.4515	0.0026	0.3586	0.0004	0.0929	0.9650
Left postcentral	-0.0463	0.5834	-0.0038	0.9412	-0.0425	0.8029	-0.1089	0.0746	-0.1293	0.0015	0.0204	0.9789
Left posterior cingulate	-0.0520	0.4893	0.0401	0.4299	-0.0921	0.7177	-0.1078	0.0437	-0.0675	0.0657	-0.0403	0.9650
Left precentral	0.0415	0.4522	0.0988	0.0023	-0.0572	0.7177	-0.0053	0.9182	0.0175	0.5288	-0.0228	0.9650
Left precuneus	-0.2052	0.0000	-0.1137	0.0005	-0.0915	0.7177	-0.2507	0.0000	-0.1936	0.0000	-0.0570	0.9320
Left rostral anterior cingulate	0.0855	0.2807	0.1693	0.0003	-0.0838	0.7177	0.0407	0.5394	0.0691	0.0954	-0.0283	0.9650
Left rostral middle frontal	0.0138	0.7940	-0.0210	0.5967	0.0347	0.8029	-0.0234	0.6069	-0.1025	0.0002	0.0792	0.9320
Left superior frontal	-0.0496	0.3231	0.0298	0.3828	-0.0794	0.7177	-0.0962	0.0093	-0.0429	0.0919	-0.0533	0.9320
Left superior parietal	-0.2461	0.0001	-0.1446	0.0006	-0.1015	0.7177	-0.2845	0.0000	-0.2353	0.0000	-0.0492	0.9320
Left superior temporal	-0.0902	0.0980	-0.0592	0.1160	-0.0309	0.8029	-0.1312	0.0008	-0.1523	0.0000	0.0211	0.9650
Left supramarginal	-0.1328	0.0555	-0.0606	0.2059	-0.0722	0.7177	-0.1749	0.0010	-0.1490	0.0000	-0.0260	0.9650

Left frontal pole	-0.4136	0.0000	-0.3556	0.0000	-0.0581	0.7864	-0.4392	0.0000	-0.3971	0.0000	-0.0421	0.9650
Left temporal pole	-0.0610	0.4957	-0.1691	0.0015	0.1081	0.7177	-0.0691	0.3601	-0.2301	0.0000	0.1609	0.9320
Left transverse temporal	-0.0899	0.3298	-0.1058	0.0732	0.0159	0.9110	-0.1449	0.0553	-0.2338	0.0000	0.0889	0.9320
Left insula	-0.0277	0.5910	-0.0502	0.1364	0.0225	0.8491	-0.0781	0.0336	-0.1361	0.0000	0.0580	0.9320
Right banks superior temporal sulcus	-0.1606	0.0256	-0.1822	0.0000	0.0216	0.8878	-0.2002	0.0010	-0.2467	0.0000	0.0466	0.9650
Right caudal anterior cingulate	0.0872	0.4986	0.2379	0.0023	-0.1507	0.7177	0.0033	0.9826	0.1084	0.1455	-0.1051	0.9320
Right caudal middle frontal	0.0196	0.7940	0.0879	0.0706	-0.0684	0.7464	-0.0222	0.7691	0.0226	0.6221	-0.0448	0.9650
Right cuneus	-0.2037	0.0384	-0.0950	0.1577	-0.1087	0.7177	-0.2467	0.0029	-0.2074	0.0002	-0.0393	0.9650
Right entorhinal	0.1960	0.0722	0.0808	0.2956	0.1152	0.7177	0.1621	0.1038	0.0134	0.8676	0.1487	0.9320
Right fusiform	-0.0144	0.7940	-0.0312	0.4382	0.0168	0.8878	-0.0474	0.2686	-0.1161	0.0000	0.0688	0.9320
Right inferior parietal	-0.1041	0.0816	-0.0883	0.0266	-0.0158	0.8916	-0.1490	0.0015	-0.1679	0.0000	0.0189	0.9723
Right inferior temporal	-0.0740	0.2697	0.0261	0.5782	-0.1001	0.7177	-0.1130	0.0221	-0.0619	0.0701	-0.0511	0.9320
Right isthmus cingulate	-0.2008	0.0198	-0.0914	0.1180	-0.1094	0.7177	-0.2704	0.0001	-0.2020	0.0000	-0.0684	0.9320
Right lateral occipital	-0.2205	0.0006	-0.1610	0.0002	-0.0595	0.7642	-0.2612	0.0000	-0.2490	0.0000	-0.0122	0.9789
Right lateral orbital frontal	-0.0895	0.0868	-0.0429	0.2401	-0.0466	0.7642	-0.1496	0.0001	-0.1446	0.0000	-0.0049	0.9789
Right lingual	-0.1906	0.0052	-0.1013	0.0291	-0.0893	0.7177	-0.2414	0.0001	-0.1797	0.0000	-0.0617	0.9320
Right medial orbital frontal	-0.0893	0.0600	-0.0945	0.0015	0.0051	0.9507	-0.1355	0.0001	-0.1812	0.0000	0.0456	0.9320
Right middle temporal	-0.0772	0.1187	-0.0074	0.8173	-0.0698	0.7177	-0.1194	0.0010	-0.0856	0.0005	-0.0338	0.9320
Right parahippocampal	0.0798	0.2697	0.0533	0.2654	0.0265	0.8638	0.0592	0.3550	-0.0013	0.9742	0.0605	0.9320
Right paracentral	-0.1171	0.0559	-0.0212	0.6202	-0.0958	0.7177	-0.1504	0.0038	-0.0803	0.0252	-0.0701	0.9320

Right pars opercularis	-0.0181	0.7940	0.0431	0.3828	-0.0612	0.7642	-0.0595	0.3471	-0.0316	0.4664	-0.0279	0.9650
Right pars orbitalis	-0.1019	0.0713	-0.0557	0.1577	-0.0462	0.7718	-0.1297	0.0053	-0.1215	0.0001	-0.0082	0.9789
Right pars triangularis	-0.0964	0.1596	-0.0197	0.6893	-0.0767	0.7177	-0.1326	0.0189	-0.0832	0.0311	-0.0494	0.9320
Right pericalcarine	0.5796	0.0000	0.6271	0.0000	-0.0475	0.8878	0.4802	0.0005	0.4527	0.0000	0.0275	0.9789
Right postcentral	-0.1475	0.0655	-0.0101	0.8417	-0.1374	0.7177	-0.2060	0.0009	-0.1298	0.0019	-0.0762	0.9320
Right posterior cingulate	0.0640	0.3906	0.0437	0.3828	0.0203	0.8878	0.0134	0.8634	-0.0448	0.2827	0.0582	0.9320
Right precentral	0.1231	0.0253	0.1251	0.0003	-0.0019	0.9724	0.0869	0.0422	0.0498	0.0884	0.0371	0.9320
Right precuneus	-0.1835	0.0002	-0.1496	0.0000	-0.0339	0.8029	-0.2276	0.0000	-0.2388	0.0000	0.0112	0.9789
Right rostral anterior cingulate	0.0310	0.7361	0.1590	0.0030	-0.1279	0.7177	-0.0038	0.9744	0.0833	0.1031	-0.0871	0.9320
Right rostral middle frontal	-0.0637	0.2690	-0.0210	0.5967	-0.0427	0.7718	-0.1037	0.0169	-0.1011	0.0005	-0.0026	0.9802
Right superior frontal	-0.0283	0.5910	0.0601	0.0684	-0.0884	0.7177	-0.0663	0.0971	-0.0144	0.6162	-0.0519	0.9320
Right superior parietal	-0.1549	0.0256	-0.1572	0.0003	0.0024	0.9724	-0.1847	0.0003	-0.2448	0.0000	0.0601	0.9320
Right superior temporal	-0.0623	0.2601	-0.0740	0.0293	0.0117	0.8995	-0.0987	0.0114	-0.1557	0.0000	0.0570	0.9320
Right supramarginal	-0.0288	0.6706	-0.0658	0.1203	0.0370	0.8029	-0.0787	0.1038	-0.1591	0.0000	0.0804	0.9320
Right frontal pole	-0.4714	0.0000	-0.3800	0.0000	-0.0915	0.7177	-0.5021	0.0000	-0.4068	0.0000	-0.0953	0.9320
Right temporal pole	-0.1461	0.0655	-0.1116	0.0338	-0.0345	0.8514	-0.1747	0.0098	-0.1731	0.0001	-0.0016	0.9831
Right transverse temporal	-0.1413	0.1201	-0.1015	0.1019	-0.0398	0.8514	-0.2088	0.0080	-0.2208	0.0000	0.0119	0.9789
Right insula	-0.0165	0.7499	0.0907	0.0027	-0.1071	0.7177	-0.0525	0.1363	0.0060	0.8329	-0.0584	0.9320

Note: Red text denotes p<0.05 (false discovery rate corrected); Regression coefficients (β) and p-values are derived from linear mixed effects models.

Supplementary Table 7a. Brain growth from term-equivalent to 7 years in very preterm children with higher or lower social risk and the difference in trajectories.

Region	Unadjusted						Adjusted for total brain tissue volume					
	High β	High *p*	Low β	Low *p*	Social risk-by-time β	Social risk-by-time *p*	High β	High *p*	Low β	Low *p*	Social risk-by-time β	Social risk-by-time *p*
Intracranial	1.8464	0.0000	1.8642	0.0000	-0.0178	0.8214	0.1138	0.0098	0.1192	0.0082	-0.0053	0.9777
Total brain tissue	1.9015	0.0000	1.9156	0.0000	-0.0141	0.8741						
Extra-axial cerebrospinal fluid	0.9811	0.0000	1.0016	0.0000	-0.0205	0.9204	0.5239	0.1167	0.5372	0.1181	-0.0133	0.9777
Cerebrospinal fluid	1.0267	0.0000	1.0688	0.0000	-0.0422	0.8874	0.8745	0.0098	0.9155	0.0082	-0.0411	0.9777
Left cortical grey matter	1.9321	0.0000	2.0137	0.0000	-0.0816	0.1575	0.3575	0.0000	0.4248	0.0000	-0.0673	0.0197
Right cortical grey matter	1.9348	0.0000	2.0168	0.0000	-0.0820	0.1575	0.4119	0.0000	0.4801	0.0000	-0.0682	0.0197
Left lateral ventricle	0.5562	0.0000	0.5625	0.0001	-0.0063	0.9747	-0.0368	0.9648	-0.0391	0.9431	0.0022	0.9910
Right lateral ventricle	0.6714	0.0000	0.7058	0.0000	-0.0344	0.9204	0.1275	0.8545	0.1543	0.8116	-0.0267	0.9777
Third ventricle	1.2424	0.0000	1.3645	0.0000	-0.1221	0.7969	1.2414	0.0118	1.3635	0.0071	-0.1221	0.9777
Fourth ventricle	1.6904	0.0000	1.6386	0.0000	0.0518	0.8474	1.5243	0.0000	1.4700	0.0000	0.0543	0.9777
Left white matter	1.8654	0.0000	1.8997	0.0000	-0.0342	0.7969	-0.6906	0.0000	-0.6854	0.0000	-0.0051	0.9777
Right white matter	1.8755	0.0000	1.9133	0.0000	-0.0378	0.7969	-0.5768	0.0000	-0.5644	0.0000	-0.0124	0.9777
Corpus callosum	1.8227	0.0000	1.8692	0.0000	-0.0465	0.7969	0.1965	0.2266	0.2202	0.1833	-0.0237	0.9777
Brainstem	1.7450	0.0000	1.7758	0.0000	-0.0308	0.7969	0.4300	0.0000	0.4395	0.0000	-0.0095	0.9777
Left cerebellum	1.9824	0.0000	1.9966	0.0000	-0.0142	0.8749	0.8757	0.0000	0.8746	0.0000	0.0011	0.9782
Right cerebellum	1.9615	0.0000	1.9728	0.0000	-0.0113	0.8874	0.8520	0.0000	0.8482	0.0000	0.0038	0.9777
Left thalamus	1.6790	0.0000	1.7089	0.0000	-0.0300	0.8214	-0.3346	0.0087	-0.3317	0.0109	-0.0029	0.9782
Left caudate	1.8815	0.0000	1.9262	0.0000	-0.0447	0.7969	0.2963	0.0234	0.3217	0.0157	-0.0254	0.9777

Left putamen	1.8462	0.0000	1.9016	0.0000	-0.0554	0.7171	0.6389	0.0000	0.6769	0.0000	-0.0380	0.9638
Left pallidum	1.7426	0.0000	1.7470	0.0000	-0.0045	0.9417	0.3977	0.0018	0.3789	0.0038	0.0188	0.9777
Left hippocampus	1.8479	0.0000	1.8805	0.0000	-0.0326	0.7969	0.4018	0.0005	0.4169	0.0004	-0.0151	0.9777
Left amygdala	1.7770	0.0000	1.8150	0.0000	-0.0380	0.7969	0.2389	0.0885	0.2527	0.0780	-0.0138	0.9777
Left accumbens	1.8379	0.0000	1.8084	0.0000	0.0296	0.8874	0.4578	0.0254	0.4103	0.0499	0.0476	0.9777
Right thalamus	1.7062	0.0000	1.7441	0.0000	-0.0380	0.7969	-0.2921	0.0161	-0.2809	0.0229	-0.0111	0.9777
Right caudate	1.8846	0.0000	1.9125	0.0000	-0.0279	0.8214	0.3500	0.0071	0.3587	0.0068	-0.0088	0.9777
Right putamen	1.8938	0.0000	1.9043	0.0000	-0.0105	0.9108	0.6956	0.0000	0.6889	0.0000	0.0067	0.9777
Right pallidum	1.7244	0.0000	1.7980	0.0000	-0.0735	0.6012	0.1354	0.3223	0.1861	0.1833	-0.0507	0.8462
Right hippocampus	1.8796	0.0000	1.9165	0.0000	-0.0368	0.7969	0.6470	0.0000	0.6710	0.0000	-0.0240	0.9777
Right amygdala	1.8770	0.0000	1.8878	0.0000	-0.0108	0.9161	0.4158	0.0004	0.4068	0.0006	0.0090	0.9777
Right accumbens	1.8814	0.0000	1.8347	0.0000	0.0467	0.7969	0.4739	0.0020	0.4066	0.0096	0.0673	0.6545
Left banks superior temporal sulcus	1.9233	0.0000	1.9339	0.0000	-0.0106	0.9262	0.4543	0.0087	0.4459	0.0114	0.0084	0.9777
Left caudal anterior cingulate	1.4407	0.0000	1.5123	0.0000	-0.0717	0.7969	-0.6024	0.0260	-0.5598	0.0432	-0.0425	0.9777
Left caudal middle frontal	1.8750	0.0000	1.8640	0.0000	0.0110	0.9204	0.7446	0.0000	0.7188	0.0000	0.0257	0.9777
Left cuneus	1.7200	0.0000	1.8969	0.0000	-0.1769	0.3478	0.0089	0.9780	0.1576	0.5159	-0.1486	0.4985
Left entorhinal	1.6868	0.0000	1.8308	0.0000	-0.1440	0.5233	1.1314	0.0000	1.2677	0.0000	-0.1363	0.5989
Left fusiform	1.9098	0.0000	1.9544	0.0000	-0.0446	0.7920	0.5123	0.0000	0.5396	0.0000	-0.0273	0.9777
Left inferior parietal	1.9050	0.0000	1.9744	0.0000	-0.0694	0.6321	0.2261	0.0777	0.2760	0.0348	-0.0499	0.7947
Left inferior temporal	1.9114	0.0000	2.0132	0.0000	-0.1018	0.3216	0.4696	0.0001	0.5508	0.0000	-0.0812	0.4020
Left isthmus cingulate	1.8951	0.0000	1.9434	0.0000	-0.0483	0.7969	-0.0028	0.9852	0.0207	0.9038	-0.0236	0.9777

Left lateral occipital	1.9243	0.0000	2.0749	0.0000	-0.1506	0.1575	0.2232	0.0968	0.3545	0.0102	-0.1313	0.1297
Left lateral orbital frontal	1.8697	0.0000	1.8970	0.0000	-0.0272	0.8214	0.0151	0.8989	0.0180	0.8674	-0.0030	0.9782
Left lingual	1.8679	0.0000	1.8905	0.0000	-0.0226	0.8874	0.8191	0.0000	0.8264	0.0000	-0.0073	0.9777
Left medial orbital frontal	1.9557	0.0000	1.9617	0.0000	-0.0060	0.9274	0.4102	0.0000	0.3942	0.0001	0.0160	0.9777
Left middle temporal	1.9312	0.0000	1.8744	0.0000	0.0568	0.6599	0.3223	0.0011	0.2411	0.0161	0.0812	0.2718
Left parahippocampal	1.7955	0.0000	1.9268	0.0000	-0.1313	0.4541	0.5274	0.0075	0.6420	0.0014	-0.1145	0.5559
Left paracentral	1.9653	0.0000	1.9226	0.0000	0.0427	0.7969	0.7917	0.0000	0.7309	0.0000	0.0608	0.8117
Left pars opercularis	1.8603	0.0000	1.8915	0.0000	-0.0312	0.8325	0.7300	0.0000	0.7498	0.0000	-0.0197	0.9777
Left pars orbitalis	1.9592	0.0000	1.9230	0.0000	0.0361	0.8214	0.6752	0.0000	0.6217	0.0001	0.0535	0.8531
Left pars triangularis	1.8187	0.0000	1.9703	0.0000	-0.1516	0.1452	0.8204	0.0000	0.9594	0.0000	-0.1389	0.1005
Left pericalcarine	0.5234	0.0000	0.4583	0.0001	0.0651	0.8749	-1.7823	0.0001	-1.8835	0.0001	0.1012	0.9777
Left postcentral	1.7583	0.0000	1.9055	0.0000	-0.1473	0.3216	-0.4596	0.0055	-0.3456	0.0395	-0.1141	0.4387
Left posterior cingulate	1.8233	0.0000	1.8802	0.0000	-0.0569	0.7969	0.1027	0.5012	0.1352	0.3817	-0.0324	0.9777
Left precentral	1.8841	0.0000	1.9011	0.0000	-0.0170	0.8874	0.3542	0.0011	0.3499	0.0016	0.0043	0.9777
Left precuneus	1.9481	0.0000	2.0748	0.0000	-0.1267	0.1575	0.5023	0.0000	0.6092	0.0000	-0.1068	0.1089
Left rostral anterior cingulate	1.7064	0.0000	1.8050	0.0000	-0.0986	0.4984	-0.1827	0.2447	-0.1094	0.5028	-0.0733	0.6519
Left rostral middle frontal	1.8868	0.0000	2.0272	0.0000	-0.1404	0.1452	0.3431	0.0007	0.4618	0.0000	-0.1188	0.1005
Left superior frontal	1.8875	0.0000	1.9994	0.0000	-0.1119	0.1575	0.4958	0.0000	0.5853	0.0000	-0.0895	0.1297
Left superior parietal	1.9304	0.0000	2.0507	0.0000	-0.1203	0.3338	0.3791	0.0039	0.4749	0.0004	-0.0958	0.4582
Left superior temporal	1.9447	0.0000	1.9947	0.0000	-0.0500	0.7920	0.2641	0.0099	0.2915	0.0059	-0.0274	0.9777
Left supramarginal	1.8521	0.0000	1.9258	0.0000	-0.0737	0.6599	0.3031	0.0347	0.3550	0.0157	-0.0519	0.8474

Left frontal pole	2.1317	0.0000	2.1197	0.0000	0.0120	0.9204	1.3487	0.0000	1.3268	0.0000	0.0220	0.9777
Left temporal pole	1.9166	0.0000	2.0436	0.0000	-0.1270	0.4588	0.7480	0.0000	0.8592	0.0000	-0.1113	0.5559
Left transverse temporal	1.7095	0.0000	1.8575	0.0000	-0.1480	0.3698	-0.6306	0.0055	-0.5072	0.0269	-0.1234	0.4985
Left insula	1.9211	0.0000	1.9523	0.0000	-0.0312	0.7969	0.3876	0.0001	0.3995	0.0001	-0.0119	0.9777
Right banks superior temporal sulcus	1.8787	0.0000	1.9811	0.0000	-0.1024	0.4786	0.7235	0.0000	0.8110	0.0000	-0.0875	0.5974
Right caudal anterior cingulate	1.3860	0.0000	1.5654	0.0000	-0.1794	0.4638	-0.9957	0.0010	-0.8437	0.0068	-0.1520	0.5920
Right caudal middle frontal	1.7512	0.0000	1.9255	0.0000	-0.1744	0.1575	0.5836	0.0009	0.7424	0.0000	-0.1588	0.1458
Right cuneus	1.8365	0.0000	1.8845	0.0000	-0.0480	0.8318	-0.2891	0.1704	-0.2741	0.2047	-0.0149	0.9777
Right entorhinal	1.6873	0.0000	1.8409	0.0000	-0.1536	0.4786	0.5184	0.0400	0.6564	0.0114	-0.1380	0.5920
Right fusiform	1.9098	0.0000	1.9267	0.0000	-0.0169	0.8874	0.4332	0.0002	0.4301	0.0002	0.0031	0.9782
Right inferior parietal	1.9224	0.0000	1.9671	0.0000	-0.0447	0.7969	0.4792	0.0002	0.5041	0.0001	-0.0249	0.9777
Right inferior temporal	1.8976	0.0000	1.9864	0.0000	-0.0888	0.4803	0.4180	0.0017	0.4884	0.0003	-0.0704	0.5988
Right isthmus cingulate	1.8668	0.0000	1.8859	0.0000	-0.0191	0.9108	-0.0684	0.7165	-0.0788	0.6693	0.0104	0.9777
Right lateral occipital	1.9554	0.0000	2.0444	0.0000	-0.0890	0.5341	0.4024	0.0078	0.4706	0.0023	-0.0682	0.6545
Right lateral orbital frontal	1.9372	0.0000	1.9485	0.0000	-0.0113	0.9161	0.0955	0.3512	0.0765	0.4770	0.0190	0.9777
Right lingual	1.9529	0.0000	1.9328	0.0000	0.0202	0.9061	0.4004	0.0208	0.3520	0.0460	0.0483	0.9777
Right medial orbital frontal	1.9449	0.0000	2.0354	0.0000	-0.0904	0.2646	0.4395	0.0000	0.5041	0.0000	-0.0646	0.3920
Right middle temporal	1.8813	0.0000	1.9067	0.0000	-0.0254	0.8214	0.5049	0.0000	0.5116	0.0000	-0.0067	0.9777
Right parahippocampal	1.8787	0.0000	1.8896	0.0000	-0.0110	0.9204	0.8534	0.0000	0.8505	0.0000	0.0030	0.9782
Right paracentral	1.8993	0.0000	1.9873	0.0000	-0.0880	0.4638	0.8616	0.0000	0.9347	0.0000	-0.0731	0.5920

Right pars opercularis	1.7839	0.0000	1.8955	0.0000	-0.1115	0.4020	0.4309	0.0066	0.5292	0.0010	-0.0983	0.4985
Right pars orbitalis	1.9907	0.0000	1.9403	0.0000	0.0504	0.7969	0.7424	0.0000	0.6748	0.0000	0.0675	0.5920
Right pars triangularis	1.8743	0.0000	1.9245	0.0000	-0.0502	0.7969	0.7524	0.0000	0.7880	0.0000	-0.0356	0.9777
Right pericalcarine	0.6015	0.0000	0.6898	0.0000	-0.0883	0.8214	-2.7117	0.0000	-2.6798	0.0000	-0.0319	0.9777
Right postcentral	1.7640	0.0000	1.9728	0.0000	-0.2088	0.1452	-0.4131	0.0148	-0.2360	0.1813	-0.1771	0.1005
Right posterior cingulate	1.7982	0.0000	1.7791	0.0000	0.0191	0.9061	0.1941	0.2447	0.1556	0.3710	0.0386	0.9777
Right precentral	1.8471	0.0000	1.9222	0.0000	-0.0751	0.4803	0.4456	0.0001	0.5003	0.0000	-0.0547	0.6519
Right precuneus	2.0016	0.0000	2.0521	0.0000	-0.0505	0.7845	0.4273	0.0000	0.4539	0.0000	-0.0266	0.9777
Right rostral anterior cingulate	1.6591	0.0000	1.7117	0.0000	-0.0526	0.7969	0.1210	0.5719	0.1535	0.4770	-0.0325	0.9777
Right rostral middle frontal	1.9083	0.0000	2.0007	0.0000	-0.0924	0.3698	0.4551	0.0001	0.5276	0.0000	-0.0726	0.4985
Right superior frontal	1.8555	0.0000	1.9911	0.0000	-0.1356	0.1452	0.4391	0.0000	0.5570	0.0000	-0.1180	0.0664
Right superior parietal	1.9763	0.0000	1.9827	0.0000	-0.0064	0.9409	0.5040	0.0000	0.4902	0.0001	0.0139	0.9777
Right superior temporal	1.9384	0.0000	1.9894	0.0000	-0.0510	0.7498	0.4930	0.0000	0.5229	0.0000	-0.0299	0.9777
Right supramarginal	1.8411	0.0000	1.9271	0.0000	-0.0859	0.4786	0.2624	0.0400	0.3259	0.0130	-0.0634	0.6086
Right frontal pole	2.0736	0.0000	2.2480	0.0000	-0.1744	0.2386	1.5920	0.0000	1.7601	0.0000	-0.1682	0.2718
Right temporal pole	1.9172	0.0000	2.0813	0.0000	-0.1641	0.2386	0.7102	0.0000	0.8565	0.0000	-0.1463	0.2809
Right transverse temporal	1.6787	0.0000	1.8351	0.0000	-0.1564	0.3698	-0.4328	0.0741	-0.3026	0.2229	-0.1302	0.5171
Right insula	1.8757	0.0000	1.9074	0.0000	-0.0316	0.7969	0.3173	0.0005	0.3286	0.0004	-0.0113	0.9777

Note: Red text denotes $p<0.05$ (false discovery rate corrected); Regression coefficients (β) and *p*-values are derived from linear mixed effects models.

Supplementary Table 7b. Brain growth from 7 to 13 years in very preterm children with higher or lower social risk and the difference in trajectories.

**Region**	Unadjusted						Adjusted for total brain tissue volume					
	High β	High *p*	Low β	Low *p*	Social risk-by-time β	Social risk-by-time *p*	High β	High *p*	Low β	Low *p*	Social risk-by-time β	Social risk-by-time *p*
Intracranial	0.1850	0.0000	0.2268	0.0000	-0.0418	0.9639	0.1082	0.0000	0.1060	0.0000	0.0022	0.9732
Total brain tissue	0.0846	0.0007	0.1318	0.0000	-0.0472	0.9639						
Extra-axial cerebrospinal fluid	0.8900	0.0000	0.9040	0.0000	-0.0141	0.9639	0.8728	0.0000	0.8789	0.0000	-0.0061	0.9732
Cerebrospinal fluid	0.8380	0.0000	0.8252	0.0000	0.0128	0.9639	0.8312	0.0000	0.8145	0.0000	0.0167	0.9732
Left cortical grey matter	-0.0420	0.1354	-0.0306	0.4805	-0.0113	0.9639	-0.1120	0.0000	-0.1304	0.0000	0.0183	0.9732
Right cortical grey matter	-0.0419	0.1346	-0.0271	0.5197	-0.0148	0.9639	-0.1104	0.0000	-0.1245	0.0000	0.0142	0.9732
Left lateral ventricle	0.2647	0.1055	0.1550	0.5197	0.1097	0.9639	0.2406	0.1143	0.1217	0.5061	0.1190	0.9732
Right lateral ventricle	0.2828	0.0863	0.2014	0.3973	0.0814	0.9639	0.2603	0.0891	0.1708	0.3465	0.0896	0.9732
Third ventricle	0.1808	0.1969	0.0008	0.9952	0.1800	0.9639	0.1807	0.1694	0.0007	0.9956	0.1800	0.9732
Fourth ventricle	0.2547	0.0032	0.1520	0.2059	0.1027	0.9639	0.2479	0.0026	0.1425	0.1394	0.1053	0.9732
Left white matter	-0.0142	0.7525	0.0013	0.9952	-0.0155	0.9639	-0.1329	0.0000	-0.1581	0.0000	0.0252	0.9732
Right white matter	-0.0184	0.6818	-0.0030	0.9938	-0.0153	0.9639	-0.1331	0.0000	-0.1586	0.0000	0.0255	0.9732
Corpus callosum	0.0785	0.1788	0.1088	0.1422	-0.0303	0.9639	0.0139	0.7781	0.0161	0.7963	-0.0023	0.9732
Brainstem	0.3095	0.0000	0.2991	0.0000	0.0103	0.9639	0.2567	0.0000	0.2288	0.0000	0.0279	0.9732
Left cerebellum	-0.0255	0.3688	-0.0304	0.4805	0.0049	0.9639	-0.0707	0.0007	-0.0928	0.0001	0.0222	0.9732
Right cerebellum	0.0148	0.6255	-0.0039	0.9938	0.0186	0.9639	-0.0305	0.1688	-0.0669	0.0066	0.0364	0.9732
Left thalamus	0.2088	0.0000	0.2993	0.0000	-0.0905	0.9639	0.1268	0.0004	0.1858	0.0000	-0.0590	0.9732
Left caudate	-0.0649	0.1148	-0.0221	0.7262	-0.0428	0.9639	-0.1320	0.0001	-0.1093	0.0048	-0.0226	0.9732

Left putamen	0.0860	0.0400	0.0034	0.9938	0.0826	0.9639	0.0388	0.2896	-0.0638	0.1222	0.1026	0.9732
Left pallidum	0.2274	0.0000	0.2826	0.0000	-0.0553	0.9639	0.1725	0.0000	0.2085	0.0000	-0.0360	0.9732
Left hippocampus	0.0855	0.0224	0.0816	0.0758	0.0039	0.9639	0.0278	0.3819	0.0019	0.9721	0.0259	0.9732
Left amygdala	0.2049	0.0000	0.2063	0.0001	-0.0015	0.9811	0.1437	0.0004	0.1184	0.0129	0.0253	0.9732
Left accumbens	-0.0538	0.4496	-0.0326	0.7888	-0.0211	0.9639	-0.1039	0.1102	-0.1105	0.1398	0.0066	0.9732
Right thalamus	0.1807	0.0000	0.1897	0.0000	-0.0090	0.9639	0.0974	0.0008	0.0805	0.0158	0.0168	0.9732
Right caudate	-0.0522	0.1936	-0.0071	0.9876	-0.0452	0.9639	-0.1192	0.0004	-0.0912	0.0180	-0.0281	0.9732
Right putamen	0.0180	0.6192	0.0562	0.2059	-0.0382	0.9639	-0.0310	0.2670	-0.0110	0.7544	-0.0201	0.9732
Right pallidum	0.2207	0.0000	0.1488	0.0075	0.0720	0.9639	0.1587	0.0001	0.0604	0.2050	0.0983	0.9732
Right hippocampus	0.0435	0.2089	0.0227	0.6671	0.0208	0.9639	-0.0081	0.7851	-0.0456	0.1831	0.0375	0.9732
Right amygdala	0.0988	0.0122	0.1274	0.0054	-0.0286	0.9639	0.0386	0.2478	0.0459	0.2318	-0.0074	0.9732
Right accumbens	0.0149	0.7855	0.0437	0.5342	-0.0288	0.9639	-0.0450	0.3134	-0.0382	0.4776	-0.0068	0.9732
Left banks superior temporal sulcus	-0.2015	0.0007	-0.1913	0.0085	-0.0102	0.9639	-0.2583	0.0000	-0.2750	0.0000	0.0167	0.9732
Left caudal anterior cingulate	0.1804	0.0318	0.1911	0.0622	-0.0107	0.9639	0.0953	0.2224	0.0755	0.4119	0.0198	0.9732
Left caudal middle frontal	0.0031	0.9460	0.0591	0.4658	-0.0560	0.9639	-0.0392	0.4228	-0.0040	0.9653	-0.0353	0.9732
Left cuneus	-0.0818	0.3014	-0.0698	0.5342	-0.0120	0.9639	-0.1509	0.0265	-0.1674	0.0319	0.0165	0.9732
Left entorhinal	0.0688	0.4354	0.0082	0.9938	0.0605	0.9639	0.0475	0.5659	-0.0233	0.8233	0.0709	0.9732
Left fusiform	-0.0502	0.1954	0.0026	0.9938	-0.0528	0.9639	-0.1042	0.0004	-0.0747	0.0296	-0.0295	0.9732
Left inferior parietal	-0.1865	0.0000	-0.1160	0.0400	-0.0705	0.9639	-0.2529	0.0000	-0.2099	0.0000	-0.0430	0.9732
Left inferior temporal	-0.0445	0.3014	-0.0256	0.7034	-0.0189	0.9639	-0.1018	0.0021	-0.1066	0.0052	0.0048	0.9732
Left isthmus cingulate	-0.0686	0.2487	-0.0777	0.3614	0.0091	0.9639	-0.1413	0.0026	-0.1841	0.0007	0.0428	0.9732

Left lateral occipital	-0.1359	0.0060	-0.1671	0.0046	0.0312	0.9639	-0.2013	0.0000	-0.2632	0.0000	0.0619	0.9732
Left lateral orbital frontal	0.0098	0.8275	0.0394	0.5197	-0.0296	0.9639	-0.0663	0.0220	-0.0620	0.0620	-0.0044	0.9732
Left lingual	-0.0476	0.3688	0.0007	0.9952	-0.0483	0.9639	-0.0903	0.0550	-0.0582	0.3035	-0.0320	0.9732
Left medial orbital frontal	-0.0820	0.0361	-0.0289	0.6306	-0.0531	0.9639	-0.1425	0.0000	-0.1104	0.0013	-0.0322	0.9732
Left middle temporal	-0.0561	0.1788	0.0112	0.9208	-0.0673	0.9639	-0.1221	0.0000	-0.0778	0.0208	-0.0443	0.9732
Left parahippocampal	0.0868	0.1954	0.0315	0.7877	0.0553	0.9639	0.0383	0.5298	-0.0400	0.5878	0.0783	0.9732
Left paracentral	-0.1067	0.0439	-0.0645	0.4130	-0.0422	0.9639	-0.1542	0.0008	-0.1310	0.0141	-0.0233	0.9732
Left pars opercularis	0.0185	0.7471	-0.0369	0.6361	0.0555	0.9639	-0.0238	0.6117	-0.1000	0.0561	0.0762	0.9732
Left pars orbitalis	-0.1529	0.0018	-0.1247	0.0420	-0.0282	0.9639	-0.2038	0.0000	-0.1947	0.0001	-0.0091	0.9732
Left pars triangularis	0.0065	0.8910	-0.1236	0.0177	0.1301	0.9639	-0.0319	0.4273	-0.1805	0.0000	0.1486	0.5886
Left pericalcarine	0.4317	0.0006	0.6053	0.0000	-0.1736	0.9639	0.3303	0.0061	0.4755	0.0005	-0.1452	0.9732
Left postcentral	-0.0181	0.7855	0.0064	0.9938	-0.0245	0.9639	-0.1089	0.0255	-0.1138	0.0420	0.0049	0.9732
Left posterior cingulate	-0.0121	0.8275	0.0171	0.8813	-0.0292	0.9639	-0.0823	0.0477	-0.0804	0.0918	-0.0019	0.9732
Left precentral	0.0740	0.0696	0.0855	0.0928	-0.0115	0.9639	0.0137	0.6633	-0.0007	0.9938	0.0144	0.9732
Left precuneus	-0.1385	0.0003	-0.1289	0.0046	-0.0095	0.9639	-0.1964	0.0000	-0.2094	0.0000	0.0129	0.9732
Left rostral anterior cingulate	0.1746	0.0015	0.0973	0.2059	0.0773	0.9639	0.1030	0.0263	-0.0075	0.9157	0.1105	0.9732
Left rostral middle frontal	-0.0084	0.8491	-0.0035	0.9938	-0.0049	0.9639	-0.0637	0.0483	-0.0850	0.0225	0.0213	0.9732
Left superior frontal	0.0021	0.9460	-0.0038	0.9938	0.0059	0.9639	-0.0525	0.0550	-0.0808	0.0103	0.0283	0.9732
Left superior parietal	-0.1923	0.0001	-0.1383	0.0227	-0.0540	0.9639	-0.2569	0.0000	-0.2260	0.0000	-0.0309	0.9732
Left superior temporal	-0.0826	0.0523	-0.0390	0.5342	-0.0436	0.9639	-0.1493	0.0000	-0.1343	0.0001	-0.0150	0.9732
Left supramarginal	-0.0875	0.0966	-0.0589	0.4658	-0.0286	0.9639	-0.1491	0.0004	-0.1466	0.0025	-0.0025	0.9732

Left frontal pole	-0.4185	0.0000	-0.3211	0.0000	-0.0974	0.9639	-0.4467	0.0000	-0.3645	0.0000	-0.0822	0.9732
Left temporal pole	-0.1334	0.0387	-0.1294	0.1318	-0.0041	0.9787	-0.1761	0.0017	-0.1957	0.0029	0.0196	0.9732
Left transverse temporal	-0.0934	0.1788	-0.1095	0.2239	0.0160	0.9639	-0.1911	0.0010	-0.2426	0.0002	0.0516	0.9732
Left insula	-0.0397	0.3014	-0.0712	0.1488	0.0315	0.9639	-0.1014	0.0004	-0.1568	0.0000	0.0554	0.9732
Right banks superior temporal sulcus	-0.1957	0.0003	-0.1534	0.0198	-0.0423	0.9639	-0.2410	0.0000	-0.2182	0.0001	-0.0228	0.9732
Right caudal anterior cingulate	0.2980	0.0010	0.0943	0.5167	0.2038	0.9639	0.2048	0.0144	-0.0432	0.6788	0.2480	0.9732
Right caudal middle frontal	0.1171	0.0374	0.0142	0.9321	0.1029	0.9639	0.0720	0.1623	-0.0519	0.3943	0.1238	0.9732
Right cuneus	-0.1318	0.0871	-0.1059	0.3353	-0.0259	0.9639	-0.2211	0.0006	-0.2281	0.0024	0.0070	0.9732
Right entorhinal	0.1522	0.0750	0.1479	0.2049	0.0043	0.9787	0.1076	0.1688	0.0819	0.3830	0.0257	0.9732
Right fusiform	-0.0466	0.2860	-0.0029	0.9938	-0.0437	0.9639	-0.1044	0.0013	-0.0859	0.0223	-0.0185	0.9732
Right inferior parietal	-0.1227	0.0073	-0.0601	0.3840	-0.0626	0.9639	-0.1791	0.0000	-0.1414	0.0009	-0.0377	0.9732
Right inferior temporal	0.0059	0.9089	-0.0214	0.8121	0.0273	0.9639	-0.0500	0.2258	-0.1049	0.0239	0.0549	0.9732
Right isthmus cingulate	-0.1508	0.0142	-0.0910	0.3008	-0.0598	0.9639	-0.2281	0.0000	-0.2003	0.0006	-0.0278	0.9732
Right lateral occipital	-0.1866	0.0004	-0.1560	0.0160	-0.0306	0.9639	-0.2474	0.0000	-0.2434	0.0000	-0.0039	0.9732
Right lateral orbital frontal	-0.0905	0.0307	-0.0386	0.5342	-0.0519	0.9639	-0.1678	0.0000	-0.1385	0.0001	-0.0293	0.9732
Right lingual	-0.1763	0.0012	-0.0800	0.3227	-0.0962	0.9639	-0.2446	0.0000	-0.1692	0.0028	-0.0754	0.9732
Right medial orbital frontal	-0.1159	0.0007	-0.0684	0.1413	-0.0476	0.9639	-0.1786	0.0000	-0.1501	0.0000	-0.0284	0.9732
Right middle temporal	-0.0656	0.0906	0.0224	0.7081	-0.0879	0.9639	-0.1211	0.0000	-0.0553	0.1043	-0.0658	0.9732
Right parahippocampal	0.0398	0.4610	0.0877	0.2239	-0.0479	0.9639	0.0001	0.9981	0.0307	0.6161	-0.0306	0.9732
Right paracentral	-0.0617	0.1936	-0.0413	0.5342	-0.0204	0.9639	-0.1032	0.0108	-0.0993	0.0314	-0.0039	0.9732

Right pars opercularis	0.0440	0.4244	-0.0320	0.7034	0.0760	0.9639	-0.0084	0.8553	-0.1066	0.0469	0.0982	0.9732
Right pars orbitalis	-0.1022	0.0235	-0.0317	0.6528	-0.0705	0.9639	-0.1505	0.0000	-0.1018	0.0181	-0.0487	0.9732
Right pars triangularis	-0.0605	0.2413	-0.0318	0.7033	-0.0287	0.9639	-0.1014	0.0193	-0.0954	0.0573	-0.0059	0.9732
Right pericalcarine	0.6926	0.0000	0.5103	0.0001	0.1823	0.9639	0.5359	0.0000	0.3268	0.0094	0.2091	0.9732
Right postcentral	-0.0374	0.5666	-0.0467	0.6249	0.0093	0.9639	-0.1239	0.0129	-0.1683	0.0032	0.0444	0.9732
Right posterior cingulate	0.0429	0.4496	0.0599	0.4805	-0.0170	0.9639	-0.0206	0.6692	-0.0296	0.6175	0.0090	0.9732
Right precentral	0.1376	0.0007	0.1176	0.0192	0.0200	0.9639	0.0850	0.0126	0.0409	0.3233	0.0441	0.9732
Right precuneus	-0.1919	0.0000	-0.1123	0.0222	-0.0796	0.9639	-0.2555	0.0000	-0.2021	0.0000	-0.0533	0.9732
Right rostral anterior cingulate	0.1804	0.0048	0.0575	0.5420	0.1229	0.9639	0.1196	0.0382	-0.0298	0.6788	0.1495	0.9732
Right rostral middle frontal	-0.0439	0.3064	-0.0330	0.6214	-0.0109	0.9639	-0.1006	0.0031	-0.1133	0.0042	0.0127	0.9732
Right superior frontal	0.0361	0.3337	0.0132	0.8662	0.0229	0.9639	-0.0171	0.5780	-0.0606	0.0788	0.0435	0.9732
Right superior parietal	-0.2052	0.0000	-0.0811	0.2793	-0.1241	0.9639	-0.2611	0.0000	-0.1643	0.0007	-0.0968	0.9732
Right superior temporal	-0.0913	0.0233	-0.0402	0.5167	-0.0512	0.9639	-0.1505	0.0000	-0.1207	0.0007	-0.0298	0.9732
Right supramarginal	-0.0863	0.0693	-0.0004	0.9952	-0.0859	0.9639	-0.1500	0.0001	-0.0880	0.0422	-0.0619	0.9732
Right frontal pole	-0.3567	0.0000	-0.4615	0.0000	0.1048	0.9639	-0.3742	0.0000	-0.4888	0.0000	0.1146	0.9732
Right temporal pole	-0.0733	0.2413	-0.1750	0.0177	0.1017	0.9639	-0.1181	0.0265	-0.2427	0.0001	0.1245	0.9732
Right transverse temporal	-0.1363	0.0637	-0.0984	0.3386	-0.0378	0.9639	-0.2252	0.0004	-0.2165	0.0028	-0.0087	0.9732
Right insula	0.0411	0.2860	0.0613	0.2239	-0.0202	0.9639	-0.0204	0.4831	-0.0240	0.4869	0.0036	0.9732

Note: Red text denotes $p<0.05$ (false discovery rate corrected); Regression coefficients (β) and *p*-values are derived from linear mixed effects models.

Supplementary Table 8a. Associations between intelligence quotient (IQ) scores at 13 years of age and brain volumetric growth from term-equivalent to 7 years.

**Region**	Adjusting for sex and age at IQ assessment							Adjusting for sex, age at IQ assessment & total brain volume						
	Interaction *p*	Combined β	Combined *p*	VP β	VP *p*	FT β	FT *p*	Interaction *p*	Combined β	Combined *p*	VP β	VP *p*	FT β	FT *p*
Intracranial	0.4475	2.0799	0.4714					0.9872	1.0067	0.8791				
Total brain tissue	0.4698	2.3105	0.4714											
Extra-axial cerebrospinal fluid	0.9161	0.4351	0.9086					0.9872	1.0364	0.8315				
Cerebrospinal fluid	0.9821	-0.2540	0.9775					0.9872	0.3295	0.8815				
Left cortical grey matter	0.9038	3.9356	0.2107					0.9872	2.5476	0.8125				
Right cortical grey matter	0.9038	3.4982	0.2903					0.9872	1.7879	0.8315				
Left lateral ventricle	0.9038	0.5095	0.9399					0.9872	1.6445	0.8125				
Right lateral ventricle	0.9038	0.2617	0.9775					0.9872	0.8901	0.8414				
Third ventricle	0.5707	-0.1576	0.9775					0.5346	0.8172	0.8373				
Fourth ventricle	0.9646	0.3099	0.9775					0.9872	0.9438	0.8315				
Left white matter	0.9038	3.3801	0.3312					0.9872	2.7258	0.8125				
Right white matter	0.9038	3.0607	0.4224					0.9872	2.2980	0.8315				
Corpus callosum	0.3892	-0.1241	0.9775					0.9872	-0.4086	0.8791				
Brainstem	0.9821	1.7677	0.5208					0.9872	1.8019	0.8125				
Left cerebellum	0.9038	2.4802	0.3417					0.9872	2.6674	0.8125				
Right cerebellum	0.9038	3.4107	0.2107					0.9872	2.7244	0.8125				
Left thalamus	0.9821	1.9548	0.4950					0.9872	-0.5537	0.8791				
Left caudate	0.9038	4.5469	0.0662					0.9872	2.2961	0.8125				

Left putamen	0.9821	3.0991	0.2107					0.9872	1.3604	0.8292				
Left pallidum	0.9821	-0.0375	0.9891					0.9872	-0.2306	0.9203				
Left hippocampus	0.3357	0.1526	0.9775					0.1033	-0.9461	0.8414				
Left amygdala	0.9161	2.0531	0.4561					0.9872	0.4663	0.8791				
Left accumbens	0.9038	4.6513	0.0662					0.9872	3.8726	0.1909				
Right thalamus	0.9038	4.4091	0.2107					0.9872	1.5359	0.8315				
Right caudate	0.9038	4.8816	0.0662					0.9872	3.2488	0.8125				
Right putamen	0.9821	2.6133	0.4453					0.9872	0.9508	0.8414				
Right pallidum	0.9038	1.4709	0.5258					0.9872	0.8529	0.8315				
Right hippocampus	0.3892	0.5467	0.8440					0.3416	-0.9168	0.8315				
Right amygdala	0.9821	1.3555	0.5993					0.9872	0.4255	0.8815				
Right accumbens	0.9161	3.2055	0.2486					0.9918	1.5820	0.8125				
Left banks superior temporal sulcus	0.9406	1.8845	0.4835					0.9872	0.5578	0.8752				
Left caudal anterior cingulate	0.9821	2.0259	0.4835					0.9872	2.5780	0.8125				
Left caudal middle frontal	0.0503	2.0224	0.4835					0.3162	0.6065	0.8752				
Left cuneus	0.9161	-1.9034	0.4832					0.9872	-1.7721	0.8125				
Left entorhinal	0.3892	-2.0984	0.4832					0.3228	-1.1504	0.8315				
Left fusiform	0.9038	2.3139	0.4714					0.9872	1.2309	0.8315				
Left inferior parietal	0.9821	1.2427	0.6199					0.9872	-0.4107	0.9099				
Left inferior temporal	0.9038	1.2551	0.6539					0.9872	1.4738	0.8315				
Left isthmus cingulate	0.9038	3.2761	0.2107					0.9872	1.6672	0.8125				

Left lateral occipital	0.9038	1.4639	0.5208					0.9872	1.2122	0.8315				
Left lateral orbital frontal	0.9038	1.2048	0.6199					0.9872	0.0770	0.9880				
Left lingual	0.9821	-1.3424	0.5208					0.9918	-1.2747	0.8125				
Left medial orbital frontal	0.9821	1.5370	0.6187					0.9872	0.7747	0.8634				
Left middle temporal	0.9038	-0.0006	0.9996					0.9872	-1.4975	0.8292				
Left parahippocampal	0.9038	1.1671	0.6199					0.9872	0.6402	0.8634				
Left paracentral	0.4475	0.8218	0.7274					0.9872	0.3963	0.8791				
Left pars opercularis	0.9038	2.8970	0.2486					0.9872	1.4655	0.8125				
Left pars orbitalis	0.9038	-1.8456	0.4832					0.9872	-2.9053	0.4009				
Left pars triangularis	0.9646	1.7605	0.5555					0.9872	-0.2015	0.9351				
Left pericalcarine	0.6309	-1.8741	0.5993					0.9872	-0.7273	0.8634				
Left postcentral	0.7325	1.5484	0.5858					0.9872	0.8109	0.8414				
Left posterior cingulate	0.9038	3.1708	0.3198					0.9872	2.4683	0.8125				
Left precentral	0.0001			4.8303	0.0486	-5.4395	0.0071	0.0022			3.6487	0.6894	-5.0620	0.1170
Left precuneus	0.9821	2.8860	0.3463					0.9872	3.2108	0.8125				
Left rostral anterior cingulate	0.3892	4.6650	0.0662					0.9872	4.1115	0.0628				
Left rostral middle frontal	0.9038	2.6671	0.3198					0.9872	1.9054	0.8125				
Left superior frontal	0.9038	2.0616	0.4534					0.9872	1.7700	0.8125				
Left superior parietal	0.9038	0.0802	0.9798					0.9872	-0.4568	0.8752				
Left superior temporal	0.3892	3.3634	0.2107					0.9872	1.4040	0.8292				
Left supramarginal	0.7196	1.3640	0.6187					0.9293	-0.8990	0.8414				

Left frontal pole	0.5669	0.8695	0.6812					0.1445	-0.2180	0.9203				
Left temporal pole	0.4475	1.2130	0.5993					0.9872	1.3742	0.8125				
Left transverse temporal	0.9038	1.6008	0.5208					0.9872	0.5385	0.8752				
Left insula	0.3892	4.3240	0.0845					0.9872	2.8951	0.8125				
Right banks superior temporal sulcus	0.9891	1.7604	0.4950					0.9872	1.1904	0.8315				
Right caudal anterior cingulate	0.4475	1.6546	0.5131					0.9872	1.4527	0.8125				
Right caudal middle frontal	0.4475	2.5034	0.3198					0.9872	2.0605	0.8125				
Right cuneus	0.9646	-1.2788	0.5993					0.9872	-0.7999	0.8414				
Right entorhinal	0.9038	0.4914	0.8938					0.9872	0.7570	0.8414				
Right fusiform	0.4475	3.0063	0.3198					0.9872	1.9977	0.8125				
Right inferior parietal	0.5940	0.2223	0.9775					0.9872	-0.9387	0.8414				
Right inferior temporal	0.9723	1.1287	0.6199					0.9872	-0.0230	0.9880				
Right isthmus cingulate	0.7121	0.6304	0.8440					0.3228	-1.7570	0.8125				
Right lateral occipital	0.9318	0.8833	0.7509					0.9872	0.0309	0.9880				
Right lateral orbital frontal	0.9821	1.9537	0.4838					0.9872	1.2768	0.8315				
Right lingual	0.9161	0.6257	0.8020					0.9872	0.8858	0.8315				
Right medial orbital frontal	0.9038	1.6906	0.5385					0.9872	0.9850	0.8414				
Right middle temporal	0.9038	-0.0829	0.9834					0.9872	-1.7084	0.8315				
Right parahippocampal	0.9038	-0.1729	0.9775					0.9872	-0.0852	0.9880				
Right paracentral	0.9038	0.2303	0.9775					0.9872	-0.5369	0.8752				

Right pars opercularis	0.3357	0.1712	0.9775					0.5346	-0.7847	0.8414				
Right pars orbitalis	0.9038	0.7296	0.7985					0.9872	-0.4266	0.8791				
Right pars triangularis	0.4475	1.1583	0.6187					0.9872	-1.2875	0.8125				
Right pericalcarine	0.9406	-0.9269	0.6199					0.9872	-0.0179	0.9880				
Right postcentral	0.9038	2.3392	0.4534					0.9872	1.7394	0.8125				
Right posterior cingulate	0.9821	2.6579	0.3258					0.9872	1.4783	0.8125				
Right precentral	0.0184			3.7242	0.1217	-4.7448	0.1800	0.7216	1.4916	0.8125				
Right precuneus	0.9161	3.2141	0.3708					0.9918	2.6647	0.8125				
Right rostral anterior cingulate	0.0221			4.6004	0.0486	-3.1486	0.4399	0.7216	1.9172	0.8125				
Right rostral middle frontal	0.9492	2.0953	0.4835					0.9872	0.9745	0.8414				
Right superior frontal	0.9821	2.2245	0.4714					0.9872	1.1409	0.8315				
Right superior parietal	0.9038	-0.1235	0.9775					0.9872	-0.7065	0.8634				
Right superior temporal	0.9161	3.5456	0.2107					0.9872	2.7258	0.8125				
Right supramarginal	0.9038	2.1408	0.4950					0.9872	1.0353	0.8414				
Right frontal pole	0.9038	0.6577	0.8400					0.9872	-1.7567	0.8125				
Right temporal pole	0.9038	1.1739	0.6187					0.9872	1.6483	0.8125				
Right transverse temporal	0.9161	-0.7439	0.7560					0.9872	-1.2598	0.8292				
Right insula	0.9038	1.3081	0.6187					0.9872	0.3015	0.9203				

VP= very preterm; FT= full-term. **Note:** Red text denotes $p<0.05$; Regression coefficients (β) and *p*-values (false discovery rate corrected) are derived from linear regression models.

Supplementary Table 8b. Associations between intelligence quotient (IQ) scores at 13 years of age and brain volumetric growth from 7 to 13 years.

Region	Adjusting for sex and age at IQ assessment							Adjusting for sex, age at IQ assessment & total brain volume						
	Interaction *p*	Combined β	Combined *p*	VP β	VP *p*	FT β	FT *p*	Interaction *p*	Combined β	Combined *p*	VP β	VP *p*	FT β	FT *p*
Intracranial	0.9292	2.2137	0.5519					0.8969	-1.0274	0.9375				
Total brain tissue	0.7803	2.9020	0.3733											
Extra-axial cerebrospinal fluid	0.7145	0.0093	0.9969					0.9801	-1.1648	0.9375				
Cerebrospinal fluid	0.6903	0.0644	0.9969					0.8765	-0.6384	0.9375				
Left cortical grey matter	0.0523	1.2369	0.7806					0.0590	0.2192	0.9920				
Right cortical grey matter	0.0689	1.2462	0.7806					0.0622	0.0693	0.9923				
Left lateral ventricle	0.7465	-0.6810	0.7806					0.4483	0.0175	0.9923				
Right lateral ventricle	0.5771	-1.0865	0.7651					0.3957	-0.2732	0.9920				
Third ventricle	0.9542	-1.6322	0.6056					0.7479	-1.3410	0.7414				
Fourth ventricle	0.9542	1.6688	0.6405					0.9706	1.5979	0.7414				
Left white matter	0.7630	1.8696	0.6354					0.8765	0.7788	0.9375				
Right white matter	0.7145	1.4797	0.7768					0.8419	0.8500	0.9375				
Corpus callosum	0.7291	3.2926	0.2642					0.6500	1.2156	0.9375				
Brainstem	0.8821	2.2122	0.6354					0.9842	0.7753	0.9375				
Left cerebellum	0.9542	3.2240	0.2665					0.8419	1.8897	0.7414				
Right cerebellum	0.7994	4.2325	0.1393					0.9342	2.9939	0.3827				
Left thalamus	0.9292	1.4761	0.7651					0.7464	-0.0042	0.9972				
Left caudate	0.8821	-2.5362	0.6354					0.7464	-1.6649	0.9144				

Left putamen	0.3623	-1.5764	0.6354					0.2095	-0.4665	0.9375				
Left pallidum	0.9542	0.2124	0.9969					0.9801	-0.2375	0.9920				
Left hippocampus	0.7465	-0.2731	0.9969					0.6438	-0.7047	0.9375				
Left amygdala	0.7145	-2.0848	0.5519					0.9291	-1.6955	0.7414				
Left accumbens	0.9542	-4.1002	0.0940					0.9801	-3.7611	0.2210				
Right thalamus	0.2977	1.1987	0.7806					0.3957	0.7642	0.9375				
Right caudate	0.1144	-2.8060	0.2501					0.2776	-2.9757	0.2693				
Right putamen	0.7291	-1.4368	0.7806					0.2276	-0.1074	0.9923				
Right pallidum	0.9292	0.0128	0.9969					0.9801	-1.2399	0.9375				
Right hippocampus	0.0097			-1.6340	0.8303	-7.9800	0.0000	0.0622	-2.3373	0.3516				
Right amygdala	0.9658	0.5765	0.9495					0.8274	-0.7162	0.9375				
Right accumbens	0.7145	-1.6448	0.7418					0.7479	-1.2889	0.9375				
Left banks superior temporal sulcus	0.7338	-0.0063	0.9969					0.4174	-0.2788	0.9920				
Left caudal anterior cingulate	0.6365	1.9154	0.5519					0.7464	1.4595	0.7414				
Left caudal middle frontal	0.0038			-2.4637	0.7081	6.9502	0.0090	0.0364			-2.2566	0.4438	4.5735	0.1148
Left cuneus	0.5997	1.1663	0.7806					0.6438	-0.0350	0.9923				
Left entorhinal	0.6903	0.3969	0.9969					0.8007	0.2624	0.9920				
Left fusiform	0.7803	1.8826	0.6056					0.8419	1.6965	0.7414				
Left inferior parietal	0.2489	-0.3359	0.9969					0.1850	-0.2716	0.9920				
Left inferior temporal	0.9658	2.2138	0.5519					0.9812	1.0624	0.9375				
Left isthmus cingulate	0.0086			-3.7628	0.7081	5.8833	0.0696	0.1850	-2.5396	0.5367				

Left lateral occipital	0.2977	3.5581	0.0940					0.3773	3.0145	0.2254				
Left lateral orbital frontal	0.1768	-1.2090	0.7806					0.0959	-2.6166	0.6051				
Left lingual	0.7803	0.2378	0.9969					0.7464	-0.5861	0.9819				
Left medial orbital frontal	0.0220			1.1849	0.9840	-5.5750	0.0023	0.4064	-1.2662	0.9375				
Left middle temporal	0.6365	0.7404	0.8295					0.1021	-0.2043	0.9920				
Left parahippocampal	0.7803	2.4120	0.5519					0.8419	1.5936	0.9144				
Left paracentral	0.7338	0.2314	0.9969					0.8419	-0.7234	0.9375				
Left pars opercularis	0.4491	0.4789	0.9969					0.5025	0.6419	0.9375				
Left pars orbitalis	0.5771	1.3805	0.7806					0.7464	0.7391	0.9375				
Left pars triangularis	0.0057			0.8138	0.9840	7.2147	0.0000	0.0507	1.3966	0.7414				
Left pericalcarine	0.9658	2.0572	0.6036					0.8627	1.9256	0.7414				
Left postcentral	0.0079			1.1338	0.9840	13.6697	0.0005	0.0622	2.0162	0.7414				
Left posterior cingulate	0.7630	-1.5783	0.6354					0.8419	-1.2883	0.7414				
Left precentral	0.9542	-0.8672	0.7806					0.7479	-2.0695	0.5367				
Left precuneus	0.4700	1.1508	0.7806					0.6302	-0.5781	0.9819				
Left rostral anterior cingulate	0.1768	0.0709	0.9969					0.3298	-0.4895	0.9920				
Left rostral middle frontal	0.1674	1.0030	0.7806					0.2878	0.1018	0.9923				
Left superior frontal	0.0038			-0.9064	0.9840	5.6051	0.0000	0.0697	-0.3243	0.9920				
Left superior parietal	0.0209			1.3349	0.8843	7.2309	0.0000	0.0590	1.3314	0.7414				
Left superior temporal	0.2974	1.6801	0.5519					0.3386	-0.1232	0.9923				
Left supramarginal	0.9292	0.8753	0.7806					0.8094	-0.1099	0.9923				

Left frontal pole	0.7291	0.2425	0.9969					0.6302	1.0452	0.9375				
Left temporal pole	0.5921	-0.5206	0.9351					0.1916	0.3039	0.9920				
Left transverse temporal	0.0556	-0.6296	0.9343					0.1850	-0.3801	0.9920				
Left insula	0.0079			-2.2241	0.7795	5.2966	0.0084	0.0622	-0.7941	0.9375				
Right banks superior temporal sulcus	0.9542	1.2192	0.7806					0.7479	-0.8890	0.9375				
Right caudal anterior cingulate	0.6723	-1.7759	0.7598					0.8007	-2.0312	0.7657				
Right caudal middle frontal	0.9761	0.0354	0.9969					0.8765	-0.3842	0.9920				
Right cuneus	0.7803	0.5158	0.9443					0.8765	-0.7399	0.9375				
Right entorhinal	0.6365	0.0063	0.9969					0.6438	-0.8255	0.9375				
Right fusiform	0.5862	-1.3687	0.7768					0.3957	-1.7758	0.7657				
Right inferior parietal	0.0086			-0.5849	0.9840	6.0141	0.0004	0.0008			-1.8249	0.8529	5.5294	0.0009
Right inferior temporal	0.9658	3.1157	0.2501	3.0779	0.7208	3.2877	0.3046	0.8420	2.4439	0.7168				
Right isthmus cingulate	0.0038			-1.1172	0.9840	5.7260	0.0000	0.0002			-1.6610	0.8529	5.4413	0.0000
Right lateral occipital	0.7145	1.4444	0.7768					0.8007	0.7857	0.9375				
Right lateral orbital frontal	0.7037	-0.4185	0.9969					0.3957	-0.6709	0.9375				
Right lingual	0.7338	0.0642	0.9969					0.8419	-1.0528	0.9375				
Right medial orbital frontal	0.9658	-1.1854	0.7806					0.9801	-1.5344	0.9375				
Right middle temporal	0.7145	0.9766	0.7806					0.6678	0.5221	0.9375				
Right parahippocampal	0.7803	1.3337	0.7768					0.8627	-0.0650	0.9923				
Right paracentral	0.7803	0.4764	0.9969					0.6004	-0.6815	0.9819				

Right pars opercularis	0.0078			0.0198	0.9842	-5.7455	0.0002	0.0003			0.1719	0.9386	-5.9076	0.0000
Right pars orbitalis	0.7803	-0.1401	0.9969					0.9801	-0.4682	0.9819				
Right pars triangularis	0.9542	0.9144	0.7806					0.7464	0.1565	0.9920				
Right pericalcarine	0.9293	0.7042	0.8541					0.5840	-0.2014	0.9920				
Right postcentral	0.7338	1.5618	0.7768					0.9801	0.8418	0.9375				
Right posterior cingulate	0.7803	2.7578	0.5519					0.9801	3.2548	0.2693				
Right precentral	0.0000			-1.4263	0.8097	6.1997	0.0000	0.0000			-2.1079	0.3336	5.3368	0.0000
Right precuneus	0.2121	0.9971	0.8155					0.0622	0.2590	0.9920				
Right rostral anterior cingulate	0.9569	-0.2686	0.9969					0.9842	-0.6474	0.9375				
Right rostral middle frontal	0.9542	0.6322	0.8541					0.8516	-0.2504	0.9920				
Right superior frontal	0.9292	1.2268	0.7806					0.6302	0.9562	0.9375				
Right superior parietal	0.0090			0.8591	0.9840	8.3196	0.0000	0.0172			-0.2088	0.9686	7.0087	0.0010
Right superior temporal	0.7803	-0.2499	0.9969					0.6302	-0.8981	0.9375				
Right supramarginal	0.0001			-2.0118	0.8097	6.2424	0.0000	0.0000			-2.8788	0.4438	5.5864	0.0000
Right frontal pole	0.9542	-1.3807	0.7768					0.8765	-1.2567	0.9375				
Right temporal pole	0.7803	1.2108	0.7806					0.8420	0.0456	0.9923				
Right transverse temporal	0.7338	0.0272	0.9969					0.7605	-0.9099	0.9375				
Right insula	0.7291	0.6579	0.8681					0.9291	0.0848	0.9923				

VP= very preterm; FT= full-term. **Note:** Red text denotes $p<0.05$; Regression coefficients (β) and *p*-values (false discovery rate corrected) are derived from linear regression models.
