# Supplementary material for: Tracking regional brain growth up to age 13 in children born term and very preterm
Source: Nat Commun. 2020 Feb 4;11:696. doi: 10.1038/s41467-020-14334-9 (PMC7000691; doi:10.1038/s41467-020-14334-9)
Supplement: Supplementary file 2 — Reporting Summary [file 41467_2020_14334_MOESM2_ESM.pdf]

nature research

Corresponding author(s): Deanne K. Thompson

Last updated by author(s): Dec 14, 2019

Reporting Summary

Nature Research wishes to improve the reproducibility of the work that we publish. This form provides structure for consistency and transparency in reporting. For further information on Nature Research policies, see Authors & Referees and the Editorial Policy Checklist .

Statistics

For all statistical analyses, confirm that the following items are present in the figure legend, table legend, main text, or Methods section.

n/a	Confirmed	
☐	☒	The exact sample size (*n*) for each experimental group/condition, given as a discrete number and unit of measurement
☐	☒	A statement on whether measurements were taken from distinct samples or whether the same sample was measured repeatedly
☐	☒	The statistical test(s) used AND whether they are one- or two-sided<br>*Only common tests should be described solely by name; describe more complex techniques in the Methods section.*
☐	☒	A description of all covariates tested
☐	☒	A description of any assumptions or corrections, such as tests of normality and adjustment for multiple comparisons
☐	☒	A full description of the statistical parameters including central tendency (e.g. means) or other basic estimates (e.g. regression coefficient) AND variation (e.g. standard deviation) or associated estimates of uncertainty (e.g. confidence intervals)
☒	☐	For null hypothesis testing, the test statistic (e.g. *F*, *t*, *r*) with confidence intervals, effect sizes, degrees of freedom and *P* value noted<br>*Give P values as exact values whenever suitable.*
☒	☐	For Bayesian analysis, information on the choice of priors and Markov chain Monte Carlo settings
☒	☐	For hierarchical and complex designs, identification of the appropriate level for tests and full reporting of outcomes
☒	☐	Estimates of effect sizes (e.g. Cohen's *d*, Pearson's *r*), indicating how they were calculated

Our web collection on statistics for biologists contains articles on many of the points above.

Software and code

Policy information about availability of computer code

Data collection	Figures were made using Matlab (MathWorks, MA).
Data analysis	Analyses were conducted using Stata 15.0 (StataCorp, TX).

For manuscripts utilizing custom algorithms or software that are central to the research but not yet described in published literature, software must be made available to editors/reviewers. We strongly encourage code deposition in a community repository (e.g. GitHub). See the Nature Research guidelines for submitting code & software for further information.

Data

Policy information about availability of data

All manuscripts must include a data availability statement. This statement should provide the following information, where applicable:

- Accession codes, unique identifiers, or web links for publicly available datasets
- A list of figures that have associated raw data
- A description of any restrictions on data availability

The datasets generated during and/or analysed during the current study are available from the corresponding author on reasonable request. The MRI data are not publicly available due to ethical restrictions.

Field-specific reporting

Please select the one below that is the best fit for your research. If you are not sure, read the appropriate sections before making your selection.

☐ Life sciences ☒ Behavioural & social sciences ☐ Ecological, evolutionary & environmental sciences

Behavioural & social sciences study design

All studies must disclose on these points even when the disclosure is negative.

Study description	Quantitative longitudinal cohort study
Research sample	Control group of very preterm and full-term infants studied at term equivalent age, 7 years of age and 13 years of age. Approximately equal numbers of male and female participants.
Sampling strategy	224 very preterm children and 45 full-term children were recruited around birth from hospital.
Data collection	Perinatal data was collected from medical records. Imaging data was collected from an MRI scanner with post-processing techniques to obtain brain region volumes. IQ was measured at 13 years of age using the Kaufman Brief Intelligence Test, Second Edition
Timing	Data collection began in 2001 and concluded 2019.
Data exclusions	Subjects were included if they did not have genetic or congenital abnormalities and survived to 13 years of age. By 13 years of age, those successfully followed up included 140 very preterm and 48 full-term children.
Non-participation	193 VP (born <30 weeks' gestation or very low birthweight, <1250 g) and 34 FT (born >37 and ≤41 weeks' gestation) infants had usable volumetric data at term, 152 VP and 34 FT children had usable data at 7 years, and 140 VP and 26 FT children had usable data at 13 years. 216 VP and 45 FT children had usable data at any time-point, and all these children were included in the analyses.
Randomization	Groups consisted very preterm (born <30 weeks' gestation or very low birthweight, <1250 g) and full-term (born >37 and ≤41 weeks' gestation) participants.

Reporting for specific materials, systems and methods

We require information from authors about some types of materials, experimental systems and methods used in many studies. Here, indicate whether each material, system or method listed is relevant to your study. If you are not sure if a list item applies to your research, read the appropriate section before selecting a response.

Materials & experimental systems

n/a	Involved in the study	
x		Antibodies
x		Eukaryotic cell lines
x		Palaeontology
x		Animals and other organisms
	x	Human research participants
x		Clinical data

Methods

n/a	Involved in the study	
x		ChIP-seq
x		Flow cytometry
	x	MRI-based neuroimaging

Human research participants

Policy information about studies involving human research participants

Population characteristics	As above
Recruitment	Participants were recruited from the Royal Women's Hospital in Melbourne, Australia, as part of the Victorian Infant Brain Study (VIBeS) prospective longitudinal cohort with no likely biases.
Ethics oversight	The study was approved by the Human Research and Ethics Committees of the Royal Women's Hospital and the Royal Children's Hospital, Melbourne. Parents gave written informed consent for their child to participate.

Note that full information on the approval of the study protocol must also be provided in the manuscript.

Magnetic resonance imaging

Experimental design

Design type	Structural volumetric MRI study
Design specifications	Volumetric measures taken at 3 time-points, term-equivalent, 7 years and 13 years of age.

Behavioral performance measures | N/A

Acquisition

Imaging type(s)	Structural
Field strength	1.5 Tesla (term-equivalent), 3 Tesla (7 and 13 years)
Sequence & imaging parameters	At term-equivalent age (38 to 42 weeks' postmenstrual age) 223 VP and 45 FT infants were scanned without sedation. Structural T2-weighted (1.7-3.0 mm coronal slices; repetition time 4000 ms; echo time 60/160 ms; flip angle 90°; field of view 220x160 mm; matrix 256x192, interpolated 512x512) images were acquired on a 1.5 Tesla General Electric MRI scanner (Signa LX Echospeed System; General Electric, Milwaukee, WI).<br><br>At 7 years' corrected age, 159 VP and 36 FT children were scanned without sedation using a 3 Tesla Trio Siemens MRI scanner (Siemens, Erlangen, Germany). T1-weighted (0.85 mm sagittal slices, repetition time 1900 ms, echo time 2.27 ms, flip angle 9°, field of view 210 x 210 mm, matrix 256 x 256) images were obtained.<br><br>At 13 years' corrected age, 140 VP and 48 FT children were similarly scanned on the 3 Tesla Trio Siemens MRI scanner. T1-weighted sequence (0.9 mm3 sagittal slices, repetition time 2530 ms, echo times 1.77, 3.51, 5.32, 7.2 ms, flip angle 7°, field of view 230 x 209 mm, matrix 256 x 230, interpolated 256 x 256) were obtained.
Area of acquisition	Whole brain scan

Diffusion MRI ☐ Used ☒ Not used

Preprocessing

Preprocessing software	At term-equivalent age, images were bias-corrected (Avants, B.B., et al. Neuroimage 54, 2033-2044 (2011)) and brain extracted (Smith, S.M. Hum Brain Mapp 17, 143-155 (2002)). For labelling, each of the T2-weighted and segmentation images from the M-CRIB atlas (Alexander, B., et al. Neuroimage 147, 841-851 (2017)) were registered to each T2-weighted images in the current sample using ANTS (Avants, B.B., et al. Neuroimage 54, 2033-2044 (2011)). pSTAPLE (Akhondi-Asl, A. & Warfield, S.K. IEEE Trans Med Imaging 32, 1840-1852 (2013)) was then used to apply the M-CRIB labels to each brain.<br><br>At 7 and 13 years of age, surface-based brain parcellation was performed on T1 images using FreeSurfer 6.0 (Fischl, B. Neuroimage 62, 774-781 (2012)), with manual editing according to FreeSurfer guidelines. ICV, total brain volume and CSF volume were obtained using Statistical Parametric Mapping version 12 (www.fil.ion.ucl.ac.uk/spm/).
Normalization	No normalization used, absolute brain volumes were used for each individual in subject space.
Normalization template	M-CRIB atlas and Freesurfer's Desikan-Killiany atlas (as above)
Noise and artifact removal	N/A
Volume censoring	Volumes were manually inspected and the subject was excluded if poor quality

Statistical modeling & inference

Model type and settings	Separate linear mixed effects or regression models fitted for each brain region.
Effect(s) tested	Group comparisons, associations with perinatal factors and IQ.

Specify type of analysis: ☐ Whole brain ☒ ROI-based ☐ Both

Anatomical location(s)	M-CRIB atlas and Freesurfer's Desikan-Killiany atlas (as above). There were 68 cortical and 14 subcortical brain regional volumes that were comparable across the 3 time-points which make up the outcomes of interest.
Statistic type for inference (See Eklund et al. 2016)	Disccete regional brain volumes.
Correction	FDR correction.

Models & analysis

n/a	Involved in the study
☒	☐ Functional and/or effective connectivity
☒	☐ Graph analysis
☒	☐ Multivariate modeling or predictive analysis
